# Terpenoids from Myrrh and Their Cytotoxic Activity against HeLa Cells

**DOI:** 10.3390/molecules28041637

**Published:** 2023-02-08

**Authors:** Katrin Kuck, Anna Unterholzner, Bartosz Lipowicz, Sebastian Schwindl, Guido Jürgenliemk, Thomas J. Schmidt, Jörg Heilmann

**Affiliations:** 1 Lehrstuhl Pharmazeutische Biologie, Universitätsstraße 31, D-93053 Regensburg, Germany; 2 Repha GmbH Biologische Arzneimittel, Alt-Godshorn 87, D-30855 Langenhagen, Germany; 3 Institut für Pharmazeutische Biologie und Phytochemie, Corrensstr. 48, D-48149 Münster, Germany

**Keywords:** myrrh, *Commiphora*, sesquiterpene, disesquiterpenes, triterpenes, HeLa

## Abstract

The oleo–gum resin of *Commiphora myrrha* (Nees) Engl. has a long history of medicinal use, although many of its constituents are still unknown. In the present investigation, 34 secondary metabolites were isolated from myrrh resin using different chromatographic techniques (silica flash chromatography, CPC, and preparative HPLC) and their structures were elucidated with NMR spectroscopy, HRESIMS, CD spectroscopy, and ECD calculations. Among the isolated substances are seven sesquiterpenes (**1**–**7**), one disesquiterpene (**8**), and two triterpenes (**23**, **24**), which were hitherto unknown, and numerous substances are described here for the first time for *C. myrrha* or the genus *Commiphora*. Furthermore, the effects of selected terpenes on cervix cancer cells (HeLa) were studied in an MTT-based in vitro assay. Three triterpenes were observed to be the most toxic with moderate IC_50_ values of 60.3 (**29**), 74.5 (**33**), and 78.9 µM (**26**). Due to the different activity of the structurally similar triterpenoids, the impact of different structural elements on the cytotoxic effect could be discussed and linked to the presence of a 1,2,3-trihydroxy substructure in the A ring. The influence on TNF-α dependent expression of the intercellular adhesion molecule 1 (ICAM-1) in human microvascular endothelial cells (HMEC-1) was also tested for **4**–**6**, **9**–**11**, **17**, **18**, **20**, and **27** in vitro, but revealed less than 20% ICAM-1 reduction and, therefore, no significant anti-inflammatory activity.

## 1. Introduction

Myrrh is the aromatic oleo–gum resin obtained from trees or shrubs of the genus *Commiphora* (Burseraceae), mainly *Commiphora myrrha*, and consists of water-soluble gum, alcohol-soluble resin, and volatile oil [1]. More than 300 molecules have already been described for the genus *Commiphora* whereby sesqui- and triterpenes are the predominating classes [2]. Several monoterpenes are also present in the volatile oil of some *Commiphora* species, such as *C. opobalsamum*, *C. mukul*, *C. tenuis*, and *C. cyclophylla* [3,4,5], but these were not detected by modern gas chromatography–mass spectrometry (GC-MS) analyses in *C. myrrha* [6,7,8,9,10,11]. Diterpenes of the abietane type occur only in some species, including *C. myrrha* [2,12].

Regarding sesquiterpenes, the most common types in the essential oil of *C. myrrha* are eudesmanes, elemanes, germacranes, and cadinanes, with furanoeudesma-1,3-diene, curzerene, and lindestrene representing the main components [6,7,9,10]. Myrrh resin also contains nonvolatile sesquiterpenoids that share structural similarities with the components of the essential oil, when excluding possible lactone structures [13,14,15,16,17]. Additionally, rather rare seco-eudesmanes and seco-cadinanes, which exhibit C-C cleavages, were isolated [18,19]. Moreover, 23 sesquiterpene dimers with varying monomers, connecting functions and, thus, new and varied skeletons, were discovered [20,21,22,23,24,25,26]. The identification of dimerized sesquiterpenes has become more feasible due to advances in technology in recent years, showing that they occur in the Asteraceae, Chloranthaceae, and Annonaceae (especially in the genus *Xylopia*) [27,28]. The dimerized sesquiterpenes are constituents of the resin, along with the nonvolatile monomeric sesquiterpenes and (mostly tetracyclic) triterpenes. Among the triterpenes from myrrh, multiple oxygenated dammaranes and cycloartanes are the most abundant in *C. myrrha* [12,15,16].

Myrrh shows both in vitro and in vivo anti-inflammatory, antiproliferative, analgesic, and antibacterial effects; however, the active compounds remain mostly unidentified [2,29]. In the clinic, Myrrhinil-intest^®^, a traditional herbal medicinal product containing a combination of myrrh, chamomile flower extract and coffee charcoal, is available in Germany for the treatment of gastrointestinal disorders with diarrhoea. This product also demonstrated efficacy in the maintenance therapy of remission in ulcerative colitis in a double-blind, double-dummy clinical trial, which provided evidence for its noninferior application against the gold-standard mesalazine [30].

Myrrh also shows antiproliferative effects against several cancer cell lines in vitro [31,32,33] and was able to show antitumour potential comparable to cyclophosphamide in a mouse study [34]. Although the antiproliferative activity of various triterpenes has been investigated many times, as well as its anti-inflammatory [35], antioxidative [36], antiviral [37], antibacterial [38], and antifungal [39] effects, data regarding triterpenes from myrrh are rare. For most triterpenes, their cytotoxic properties are limited in cancer cells, and just a low impact with normal cells is observed [40].

The aim of this study was to identify new compounds from myrrh and to complete the complex secondary metabolite profile of the plant resin. This will contribute to a better understanding of its composition and various pharmacological effects. Previous research has already shown that sesquiterpenes from myrrh can induce a reduction in ICAM-1 expression [18]. Therefore, selected and mainly sesquiterpenoid isolates were analysed in vitro in a tumour necrosis factor α (TNF-α)-dependent ICAM-1 expression assay in HMEC-1 cells to test anti-inflammatory activity. Moreover, mainly newly discovered triterpenoids were applied in an MTT assay to monitor their effects on the viability of a human tumour cell line (HeLa) and, therefore, broaden the knowledge on the relationship between the triterpenoid structure and cytotoxic activity.

## 2. Results

An ethanolic extract was gained by alternating maceration and percolation of myrrh resin and split into two parts by a liquid–liquid partition between methanol and n-heptane. Both fractions were processed using various chromatographic techniques including flash chromatography on silica gel, centrifugal partition chromatography (CPC), and preparative HPLC. Hereby, 22 sesquiterpenes and 12 triterpenes were isolated and their structures were elucidated with NMR, high resolution electrospray ionisation mass spectrometry (HRESIMS), circular dichroism (CD) spectra, and electronic circular dichroism (ECD) calculations. Among the isolated substances were ten compounds (**1**–**8**, **23**, and **24**) that were previously unknown, seven substances (**9**, **17**–**21**, and **34**) that are described here for the first time for the genus *Commiphora*, and five (**28** and **30**–**33**) that have not been detected in *C. myrrha* until now. For some known compounds, characterization was completed or revised (**10**, **11**, **13**, **30**, and **33**). The isolation of compounds **15** and **27** from a myrrh extract was recently published [41].

### 2.1. Sesquiterpenoids

**Figure 1:**
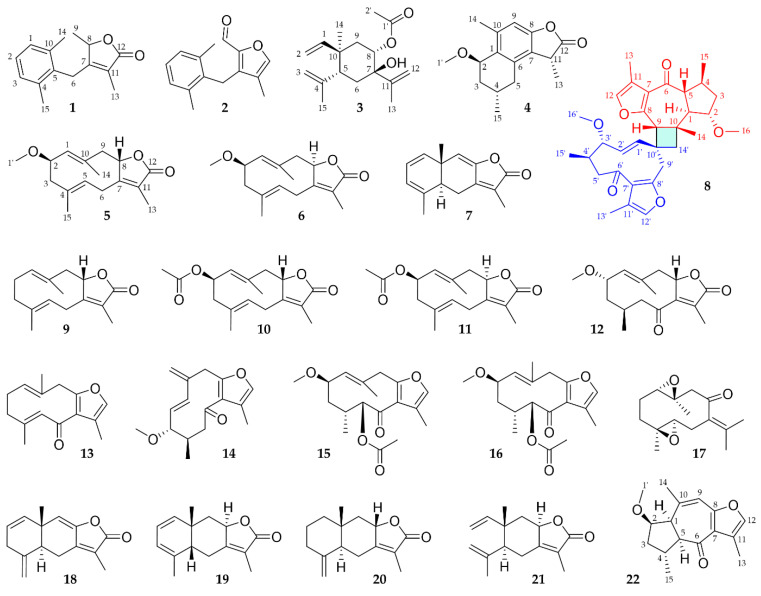
Chemical structures of isolated sesquiterpenes (**1**–**7** and **9**–**22**) and the sesquiterpene dimer (**8**) in their relative configuration. For compound **14**, absolute stereochemistry is depicted.

All isolated sesquiterpenoids are shown in Figure 1.

**Figure 2:**
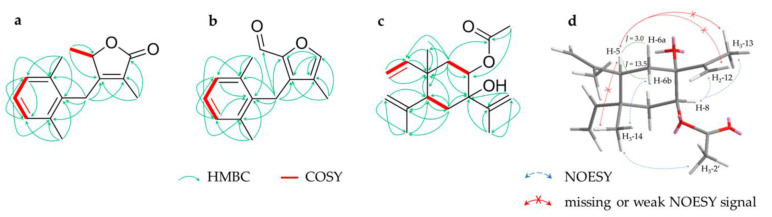
Key HMBC and COSY correlations for compounds **1** (**a**), **2** (**b**) and **3** (**c**) and relevant (or missing) NOESY signals for compound **3** (**d**); *J* in Hz.

Compound **1** (1.0 mg) was obtained as a colourless oil and its molecular formula C_15_H_18_O_2_ was determined with positive-mode HRESIMS analysis (*m*/*z* 231.1383 [M+H]^+^, calc. 231.1380). The ^1^H and ^13^C NMR spectra showed signals assignable to four methyls (*δ_H_* 1.29 (d, H_3_-9), 1.47 (brs, H_3_-13), 2x 2.24 (s, H_3_-14/15); *δ_C_* 18.7, 8.3, 2x 20.3, respectively), one sp^3^ methylene (*δ_H_* 3.64/3.72 (d/d, H_2_-6); *δ_C_* 28.3), one oxygenated sp^3^ methine (*δ_H_* 4.72 (q, H-8); *δ_C_* 79.1), three aromatic methines (*δ_H_* 2x 7.02 (d, H-1/3), 7.09 (dd, H-2); *δ_C_* 2x 128.5, 127.3, respectively), five quaternary carbons (*δ_C_* 123.4 (C-11), 132.7 (C-5), 2x 136.8 (C-4/10), 160.9 (C-7)) and one carbonyl carbon (*δ_C_* 174.4 (C-12)) (Table 1). The doubled integrals of the conjunct proton signals of pos. 1/3 and 14/15 together with the HMBC correlations of H-1/3 with C1/3 indicate a symmetric 2,6-dimethylphenyl moiety. Based on HMBC correlations, the moiety is linked via pos. 6 to a second five-membered ring, which was elucidated as an *α*,*β*-unsaturated *γ*-lactone with two methyl groups by COSY and HMBC correlations (Figure 2a). Thus, the molecule was identified as a novel seco-eudesmane-type sesquiterpene. This structural class is characterized by a cleaved C-C bond between pos. 9 and 10 and the scaffold has only been described once for two compounds of *C. myrrha* [18]. The proposed name for compound **1** is 9,10-seco-isolindestrenolide.

Compound **2** (4.1 mg) was isolated as a yellow oil with the molecular formula C_15_H_16_O_2_, deduced from positive-mode HRESIMS (*m*/*z* 229.1226 [M+H]^+^, calc. 229.1223). Its NMR data showed similarities to **1** and led to the elucidation of an identical 2,6-dimethylphenyl moiety. In **2**, this moiety is linked to a methyl-furancarboxaldehyde validated by an aromatic methine (*δ_H_* 7.37 (s, H-12); *δ_C_* 144.8), three quaternary carbons (*δ_C_* 135.9 (C-7), 149.2 (C-8), 123.5 (C-11)), a methyl (*δ_H_* 1.83 (s, H_3_-13); *δ_C_* 8.1) and an aldehyde function (*δ_H_* 8.84 (s, H-9); *δ_C_* 178.5) (Table 1, Figure 2b). The elucidated achiral structure, 9-oxo-9,10-seco-isolindestrene, is the second newly found representative of a seco-eudesmane-type sesquiterpene, after **1**.

Compound **3** (1.2 mg) was extracted as a colourless oil and its molecular formula C_17_H_26_O_3_ was determined with positive-mode HRESIMS (*m*/*z* 279.1950 [M+H]^+^, calc. 279.1955). According to NMR data, the structure contains an acetyl substituent (*δ_H_* 2.00 (s, H_3_-2′); *δ_C_* 21.2 (C-2′) and 169.7 (C-1′)), three methyls (*δ_H_* 1.79 (s, H_3_-13), 1.06 (s, H_3_-14), 1.76 (s, H_3_-15); *δ_C_* 18.5, 18.6, 24.9, respectively), two sp^3^ methylenes (*δ_H_* 1.54/2.35 (ddd/dd, H_2_-6), 1.56/2.06 (dd/dd, H_2_-9); *δ_C_* 33.3, 38.3, respectively), three sp^2^ methylenes (*δ_H_* 4.90/4.93 (brd/dd, H_2_-2), 4.69/4.90 (brs/brs, H_2_-3), 4.95/5.05 (brs/brs, H_2_-12); *δ_C_* 110.7, 112.8, 112.4, respectively), two sp^3^ methines (*δ_H_* 2.57 (dd, H-5), 4.97 (m, H-8); *δ_C_* 46.2, 72.3, respectively), one sp^2^ methine (*δ_H_* 5.83 (dd, H-1); *δ_C_* 149.6) and four quaternary carbons (*δ_C_* 146.8 (C-4), 73.9 (C-7), 39.0 (C-10), 148.5 (C-11)) (Table 1). Based on 2D NMR data, an elemene-type sesquiterpene was elucidated showing two typical isopropenyl substituents. Positions 7 and 8 are oxygenated, as deduced from characteristic ^13^C-NMR shifts and the acetyl group is located to pos. 8 due to HMBC signals (Figure 2c). Analysis of NOESY spectra of elemane-type sesquiterpenes is challenging because of the spatial flexibility of the substituents, resulting in signals to neighbours on both sides of the cyclohexane. Nonetheless, a signal between distant H_3_-14 and H_3_-2′ indicates these groups are located on the same side of the ring. The missing signal between H_3_-14 and H-5 together with the large coupling constant between H-5 and H-6b (*δ_H_* 2.35; ^3^*J*_H-5, H-6b_ = 13.5 Hz) suggests opposite axial positions of H_3_-14 and H-5. Missing signals between axial H-5 to the isopropenyl substituent H_2_-12/H_3_-13 indicate that its location is on the other side of the cyclohexane (Figure 2d). Thus, the molecule is *rel*-8*S*-acetyloxy-7*R*-hydroxy-5*R*,10*R*-β-elemene. Related structures, such as β-elemene itself, are known as constituents of myrrh [42], whereas the substitution pattern of **3** is described here for the first time.

**Figure 3:**
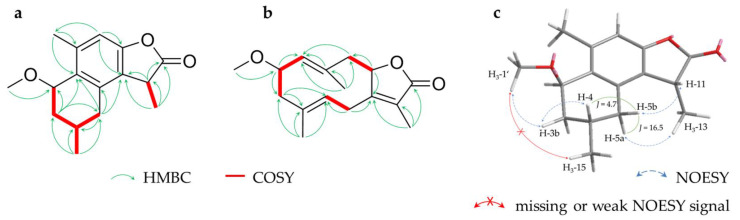
Key HMBC and COSY correlations for compounds **4** (**a**), the diastereomers **5** and **6** (**b**) as well as relevant (or missing) NOESY signals for compound **4** (**c**); *J* in Hz.

Compound **4** (0.8 mg), a colourless oil, was found to have a molecular formula of C_16_H_20_O_3_ using positive-mode HRESIMS (*m*/*z* 261.1490 [M+H]^+^, calc. 261.1485). NMR data revealed four methyls including one methoxy group (*δ_H_* 1.55 (d, H_3_-13), 2.37 (s, H_3_-14), 1.13 (d, H_3_-15), 3.44 (H_3_-1′); *δ_C_* 15.4, 19.3, 22.1, 56.2, respectively), two sp^3^ methylenes (*δ_H_* 1.28/2.28 (m/m, H_2_-3), 2.24/2.72 (m/ddd, H_2_-5); *δ_C_* 34.0, 36.2, respectively), three sp^3^ methines (*δ_H_* 4.33 (dd, H-1), 2.08 (m, H-4), 3.64 (m, H-11); *δ_C_* 73.9, 22.7, 38.2, respectively), one aromatic methine (*δ_H_* 6.84 (s, H-9); *δ_C_* 110.7), five aromatic quaternary carbons (*δ_C_* 130.1 (C-1), 134.8 (C-6), 123.3 (C-7), 152.6 (C-8), 140.1 (C-10)) and a carbonyl carbon (*δ_C_* 178.6 (C-12)) (Table 2). On the basis of 2D NMR spectra, the structure was identified as a cadinane-type sesquiterpene with an aromatic B-ring, a methoxy function in pos. 2 and a *γ*-lactone moiety, previously described as commiterpene D [18] (Figure 3a). In accordance with nomenclature established by Xu et al. [13], compound **4** was named commiterpene E.

NOESY experiments were carried out to determine the relative configuration of the three stereocenters in compound **4**. The substituents in pos. 2 and 4 must be on opposite sides of the ring due to the strong NOESY correlations between H_3_-1′ to H-3b (*δ_H_* 2.28) and H-3b to H-4. The coupling constants of H-2/H-3_a,b_ and H-5b (*δ_H_* 2.72)/H-4 being less than 5 Hz indicate their equatorial position, resulting in an opposing orientation that necessitates their location under and above the ring, respectively. This corroborates the position of H_3_-13 on the same side as H_3_-15, due to the strong NOESY signal to H-5a (*δ_H_* 2.24), whereas H-11 correlates with H-5b (Figure 3c). Thus, the configuration of compound **4** was determined to be *rel*-2*R*,4*S*,11*R*.

Compounds **5** and **6** (2.9 and 6.5 mg) were isolated as white crystals. The positive-mode HRESIMS suggested that they were isomers with the same molecular formula of C_16_H_22_O_3_ (*m*/*z* (**5**) 263.1646 [M+H]^+^, *m*/*z* (**6**) 263.1646 [M+H]^+^, calc. 263.1642). Further investigations of NMR data showed that **5** and **6** had similar resonances. Compound **5** has four methyls (*δ_H_* 1.88 (brs, H_3_-13), 1.44 (s, H_3_-14), 1.61 (s, H_3_-15), 3.35 (s, H_3_-1‘); *δ_C_* 9.3, 19.6, 18.0, 57.4, respectively), three sp^3^ methylenes (*δ_H_* 1.96/2.55 (dd/dd, H_2_-3), 3.14/3.34 (dd/dd, H_2_-6), 2.23/3.06 (dd/dd, H_2_-9); *δ_C_* 45.9, 27.0, 42.9, respectively), two sp^3^ methines (*δ_H_* 4.13 (ddd, H-2), 5.12 (dd, H-8); *δ_C_* 78.2, 83.3, respectively), six sp^2^ hybridized positions (*δ_H_* 4.99 (d, H-1), 4.96 (dd, H-5); *δ_C_* 132.1 (C-1), 134.8 (C-4), 124.9 (C-5), 162.0 (C-7), 135.4 (C-10), 125.9 (C-11)), and one carbonyl carbon (*δ_C_* 173.5 (C-12)) (Table 2). Compound **6** also has four methyls (*δ_H_* 1.87 (brs, H_3_-13), 1.57 (s, H_3_-14), 1.67 (brs, H_3_-15), 3.34 (s, H_3_-1‘); *δ_C_* 8.9, 17.4, 18.0, 57.4, respectively), three sp^3^ methylenes (*δ_H_* 1.94/2.54 (dd/dd, H_2_-3), 2.88/3.42 (dd/brd, H_2_-6), 2.12/3.09 (dd/dd, H-9); *δ_C_* 45.3, 27.7, 46.8, respectively), two sp^3^ methines (*δ_H_* 4.16 (ddd, H-2), 4.93 (dd, H-8); *δ_C_* 77.2, 82.6, respectively), six sp^2^ hybridized positions (*δ_H_* 4.89 (d, H-1), 4.56 (dd, H-5); *δ_C_* 133.3 (C-1), 133.3 (C-4), 126.1 (C-5), 134.5 (C-10), 126.3 (C-11), 162.2 (C-7)) and one carbonyl carbon (*δ_C_* 173.6 (C-12)) (Table 2), indicating that **5** and **6** are diastereomers.

**Figure 4:**
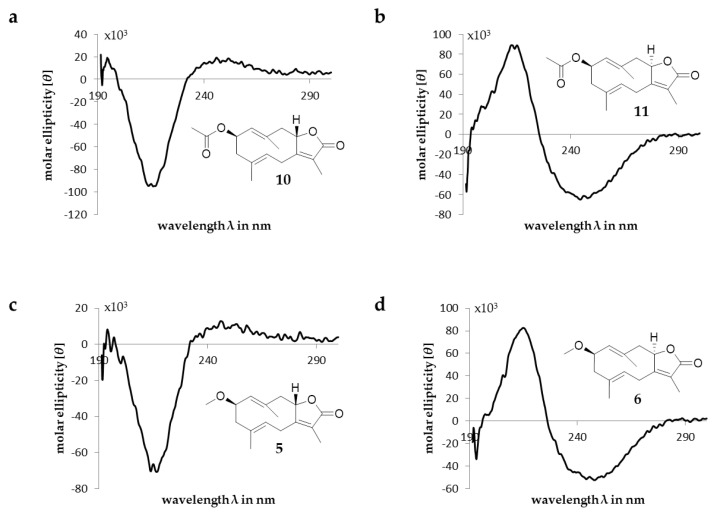
CD spectra and structures of compounds **10**, **11**, **5** and **6** (**a**–**d**). [*θ*] in °∙cm^2^∙dmol^−1^.

1D and 2D NMR data of compounds **5** and **6** revealed strong similarities to the already-known germacranolide glechomanolide (**9**, Table S1). Both compounds possess an additional methoxy group, indicated by methyl signals *(δ_H_* 3.35 ppm (**5**), 3.34 ppm (**6**); *δ_C_* 57.4) with a typical chemical shift and an oxygenation in pos. 2 (*δ_H_* 4.13 ppm (**5**), 4.16 ppm (**6**); *δ_C_* 78.2 (**5**), 77.2 (**6**)) and HMBC correlations between H_3_-1‘ and C-2 (Figure 3b). Nevertheless, ^1^H and ^13^C NMR data of **5** and **6** had different shift values and coupling constants of positions 5, 6, 8, 9 and 14, as do the two known acetyloxy-glechomanolide derivatives **10** and **11**, whose relative configurations have already been determined by Greve et al. [15]. This indicates that the two pairs of diastereomers correspond in their configurations. Furthermore, the strong similarity of **5** to **10** and **6** to **11** can be confirmed by their CD spectra (Figure 4). For a safe stereochemical determination of both stereocenters per molecule, the CD spectra of both possible diastereomeres would be necessary. Although, as the spectra are both so closely corresponding to one diastereomer just in the substituent-differing compounds, the authors are daring to propose the same relative configurations: a (*rel*-2*R*,8S) configuration can be postulated for **5**, whereas for compound **6**, a (*rel*-2*R*,8*R*) configuration can be assumed. In analogy to **10** and **11**, the semitrivial names 2β-methoxyglechomanolide and 8-*epi*-2β-methoxyglechomanolide are suggested for the previously unknown compounds **5** and **6**.

**Figure 5:**
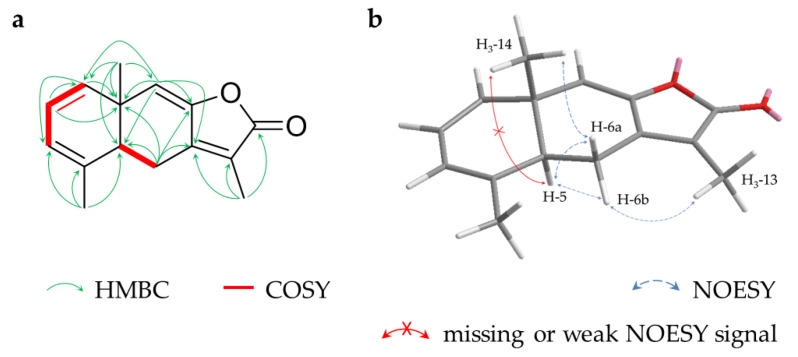
Key HMBC, COSY (**a**) and (missing) NOESY (**b**) correlations for compound **7**.

Compound **7** (1.8 mg) with a molecular formula of C_15_H_16_O_2_, as determined with positive-mode HRESIMS (*m*/*z* 229.1228 [M+H]^+^, calc. 229.1223), was isolated as a colourless oil. NMR spectra showed three methyls (*δ_H_* 1.93 (brs, H_3_-13), 1.02 (s, H_3_-14), 1.90 (brs, H_3_-15); *δ_C_* 8.6, 17.9, 20.0, respectively), one sp^3^ hybridized methylene (*δ_H_* 2.54/2.96 (ddd/dd, H_2_-6); *δ_C_* 21.3), one sp^3^ methine (*δ_H_* 2.87 (brd, H-5); *δ_C_* 44.3), four sp^2^ methines (*δ_H_* 5.72 (d, H-1), 5.86 (dd, H-2), 5.79 (m, H-3), 5.77 (s, H-9); *δ_C_* 135.0, 123.7, 120.9, 117.7, respectively), five quaternary carbons including four olefinic carbons (*δ_C_* 135.8 (C-4), 148.5 (C-7), 147.9 (C-8), 37.8 (C-10), 121.1 (C-11)) and a carbonyl carbon (*δ_C_* 171.2 (C-12)) (Table 3). The investigation of 2D NMR data of **7** led to a structure similar to isohydroxylindestrenolide [15]. The previously unknown dehydroisolindestrenolide was determined to have an analogue opposite position of C-14 and H-5, based on NOESY data (Figure 5a,b).

Compound **8** (0.9 mg) was obtained as a white solid, and its molecular formula was determined with positive-mode HRESIMS to be C_32_H_40_O_6_ (*m*/*z* 521.2904 [M+H]^+^, calc. 521.2898). Based on NMR spectra, a dimerized sesquiterpenoid was identified and the following signals were assigned: two methoxy groups (*δ_H_* 3.33 (s, H_3_-16), 3.29 (s, H_3_-16′); *δ_C_* 56.3, 56.6, respectively), five methyls (*δ_H_* 2.21 (s, H_3_-13), 1.18 (s, H_3_-14), 1.12 (d, H_3_-15), 2.01 (s, H_3_-13′), 1.15 (d, H_3_-15′); *δ_C_* 10.2, 25.6, 19.4, 9.4, 18.5, respectively), four sp^3^ methylenes (*δ_H_* 1.14/2.01 (m/m, H_2_-3), 2.39/2.46 (d/dd, H_2_-5′), 2.59/2.96 (d/d, H_2_-9′), 1.84/2.39 (d/m, H_2_-14′); *δ_C_* 39.8, 48.5, 35.1, 42.9, respectively), seven sp^3^ methines, of which two were oxygenated (*δ_H_* 2.71 (d, H-1), 3.48 (m, H-2), 2.70 (m, H-4), 2.29 (dd, H-5), 3.50 (s, H-9), 3.03 (dd, H-3′), 2.57 (m, H-4′); *δ_C_* 57.5, 83.5, 33.1, 59.2, 54.7, 88.9, 37.4, respectively), four sp^2^ methines, of which two were aromatic (*δ_H_* 7.16 (s, H-12), 5.49 (d, H-1′), 5.58 (d, H-2′), 6.92 (s, H-12′); *δ_C_* 139.2, 143.4, 133.6, 138.3, respectively) and ten quaternary carbons, of which two were aliphatic including the spiro carbon (*δ_C_* 36.1 (C-10), 42.0 (C-10′)), six were aromatic (*δ_C_* 122.5 (C-7), 159.0 (C-8), 121.1 (C-11), 117.6 (C-7′), 153.5 (C-8′), 128.6 (C-11′)) and two were carbonyl carbons (*δ_C_* 196.5 (C-6), 202.4 (C-6′)) (Table 3).

**Figure 6:**
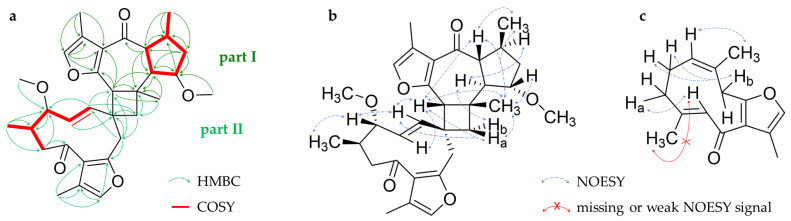
Key HMBC, COSY (**a**) and NOESY correlations for compound **8** (**b**) and key (missing) NOESY signals for compound **13** (**c**).

Analysis of 2D NMR data revealed a dimer composed of a guaiane and a germacrene-type sesquiterpene, connected by a four-membered spirocycle (Figure 6a). More precisely, the spectra revealed a structure resembling commiphorine A [21]. The guaiane moiety (part I) is the C-1 epimer of the known monomer guaiane myrrhterpenoid O (**22**) [15] and is, thus, the same as described for the dimers commiphorine A and commiphoratone B [22]. Part (II) varies from commiphorine A. First, the double bond between C-1′ and C-2′ is *E*-configurated, deduced from NOESY signals as well as the large coupling constant of ^3^*J*_H-1’, H-2’_ = 16.0 Hz. Second, an additional methoxy substituent is located at C-3′. Third, according to the NOESY signals, the stereochemistry at the spiro-C-10′ is reversed, resulting in contrary axial chirality. The germacrene moiety (part II) was also isolated as a monomer (**14**), which is already known for *C. myrrha* as 3*S*-methoxy-4*R*-furanogermacra-1*E*,10(14)-dien-6-one [43,44]. Key HMBC signals between H-9 to C-1′/C-10′ and H-9b’ to C-9 indicate a connection between the two sesquiterpene monomers (Figure 6a).

The relative configuration of compound **8** was partially elucidated by the NOESY experiments. Its spatial arrangement at C-10’ is deviating from that of commiphorine A indicated by signals between H-9 and H-1′/H-2′ on the one hand and the correlation between H-1 and H-9a’ on the other hand (Figure 6b). Due to the distance, the configuration of the stereocenters C-3′ and C-4′ was not determinable in relation to the rest of the molecule, resulting in four possible absolute configurations. Therefore, the CD spectrum was compared to those of related molecules, and the determination of the absolute configuration was then attempted via EDC calculations.

**Figure 7:**
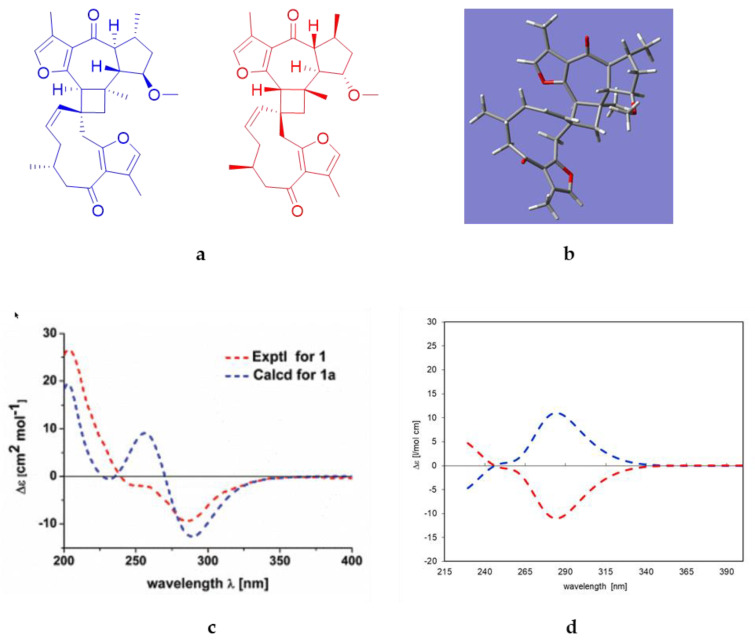
(**a**) Blue: Structure of commiphorine A according to [21], red: Enantiomeric structure; (**b**) minimum energy conformer of the blue structure depicted in (**a**) after a conformational search and full energy minimization (MMFF94x force field/low mode dynamics/energy minimization by MOPAC/PM3 followed by DFT B3LYP/6−31G(d,p)). (**c**) Experimental ECD spectrum of commiphorine A (red curve) and simulated ECD spectrum (blue curve) according to [21]. (**d**) Blue: ECD spectrum simulated by TDDFT (B3LYP/6−31G(d,p)) in the present study with the postulated [21] structure of commiphorine A (blue structure in **a**). Red: Simulated ECD spectrum of the enantiomer of the postulated [21] structure (red structure in **a**). Note the very close fit of the red curve with the experimental spectrum shown in (**c**).

As described above, compound **8** was found to be closely related to commiphorine A, reported by Dong et al. in 2019 [21] as a constituent of myrrh resin (*Resina Commiphora*). The authors also published the absolute configuration of their compound, which was based on the comparison of the measured electronic CD (ECD) spectrum with simulated quantum-mechanical computations (see Figure 7).

The ECD spectrum simulated in the present study for the postulated structure of commiphorine A [21] (Figure 7d, blue curve) is opposite to the experimental spectrum and simulation of the previous authors (Figure 7c, blue curve); i.e., the Cotton effect (CE) at the longest wavelength (about 290 nm) is positive, not negative. Due to this discrepancy, the structural models underlying the published ECD, shown in the Supplementary Materials of [21], were carefully inspected. There, the models used a compound with a 1,5-*cis*-guaianolide moiety, as opposed to the 1,5-*trans* structure postulated for commiphorine A, as determined from NMR spectroscopy (see Figure S1, Supplementary Materials). For comparison, we also simulated the ECD spectrum using the 1,5-*cis*-configured structures (see Figure S1, Supplementary Materials). These molecular models (from the original coordinates published in the Supplementary Materials of [21]) yield an ECD spectrum with a negative CE at their lowest energy transition, but they do not reflect the postulated 1,5-*trans*-configured chemical structure.

In conclusion, the experimental ECD spectrum of commiphorine A actually corresponds to the enantiomeric structure postulated in [21]. The ECD spectrum of the enantiomer (red line in Figure 7d) fits the compound’s experimental spectrum (red line in Figure 7c). On these grounds, we revised the structure of commiphorine A to be the enantiomer of the one previously published (red structure in Figure 7a).

**Figure 8:**
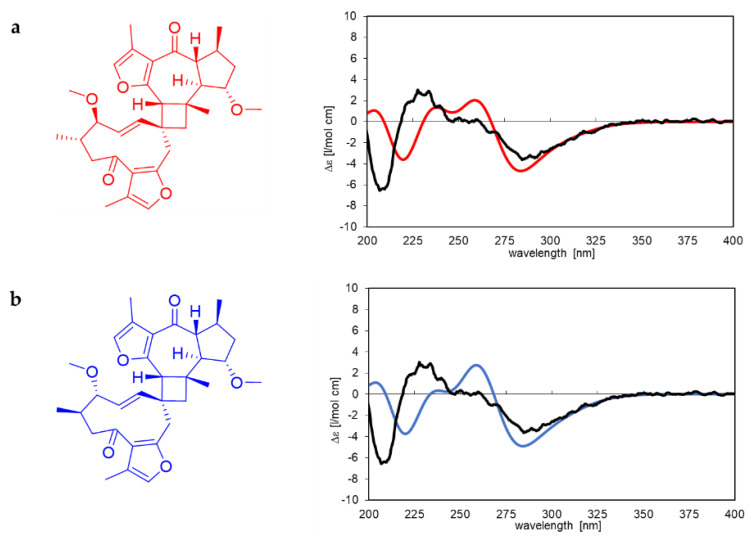
Structures and corresponding simulated ECD spectra of two possible absolute configurations of compound **8** are represented in (**a**,**b**), each shown in the same colour. The experimental ECD spectrum of commiphorine C (**8**) is depicted as a black line in both diagrams. The simulation was carried out with TDDFT (B3LYP/6−31G(d,p)).

The ECD spectrum of compound **8** also closely resembles commiphorine A. The spectrum, along with simulated spectra for two of the four possible absolute structures, is shown in Figure 8. A very prominent negative CE at about 290 nm is indicative of a guaiane moiety with the same absolute configuration as the revised structure of commiphorine A (compare Figure 8 with Figure 7). As the two calculated spectra are similar and neither fits the experimental one significantly better, the absolute configuration of commiphorine C (**8**) can be assumed using the proposed biosynthesis as presented in the discussion chapter (Figure 14). As the germacrane moiety was also isolated separately as a monomer (**14**), the absolute configuration of part II in the dimerized molecule is proposed to have the same: 3′*S*,4′*R* (determined previously with CD spectra). The structure of the new compound **8** is, thus, suggested to be (2R,2aR,6aR,6’R,7S,7’S,9S,9aR,9bS,E)- 7’,9-dimethoxy-3’,5,6’,7,9b-pentamethyl-6a,6’,7,7’,8,9,9a,9b-octahydro-1H,5’H-spiro[cyclo-buta[7,8]azuleno[6,5-b]furan-2,10’-cyclodeca[b]furan]-4’,6(2aH,11’H)-dione, as depicted in blue in Figure 8b. We propose the name commiphorine C for this new dimeric sesquiterpenoid.

Compounds **10** and **11** (10.9 and 11.3 mg) were identified as 2β-acetyloxyglechomanolide and 8-*epi*-2β-acetyloxyglechomanolide by matching their NMR data with the ones published by Greve et al. who isolated them in a 2:1 mixture from *C. myrrha* [15]. In the present work, these compounds were separated for the first time with preparative HPLC on a biphenyl column and could be characterized by UV, CD spectra and polarimetry.

Compounds **13** and **22** were isolated and elucidated as a mixture (1.6 mg in total). The ^1^H and ^13^C NMR data of compound **13** (Table S2) are consistent with a structure called 1(10)*Z*,4*Z*-furanodienone, which has been isolated from *C. myrrha* before [45]. NOESY signals between H-5 and H-3a (*δ_H_* 1.80), H-1 and H_3_-14, and H_2_-2 and H-9b (*δ_H_* 3.59) (Figure 6c) indicate another, not-yet-published configurational isomer: 1(10)*Z*,4*E*-isofuranodienone. This is also supported by the chemical shifts in the methyl carbons C-14 (*δ_C_* 22.7) and C-15 (*δ_C_* 19.2) above and below 20 ppm, which indicate a *cis*-configurated 1(10) double bond as well as a 4*E* configuration [46]. Therefore, the structure of 1(10)*Z*,4*Z*-furanodienone can be corrected to 1(10)*Z*,4*E*-isofuranodienone, in analogy to the *Curcuma zedoaria* molecule 1(10)*E*,4*Z*-isofuranodienone [47].

The other compounds were also elucidated from their NMR spectra and identified as described in the literature: glechomanolide (**9**) [48], 2α-methoxy-6-oxogermacra-1(10),7(11)-dien-8,12-olide (**12**) [49], and 3*S*-methoxy-4*R*-furanogermacra-1*E*,10(14)-dien-6-one (**14**) [43], which could be established in its absolute configuration by comparison with Santoro et al.’s calculated CD spectra [44], 2-methoxy-5-acetoxyfuranogermacr-1(10)-en-6-one (**15**) [50], *rel*-2*R*-methyl-5*S*-acetoxy-4*R*-furanogermacr-1(10)*Z*-en-6-one (**16**) [51], (1*S*,4*S*,5*S*,10*S*)-germacron-1(10),4-diepoxide (**17**) [52,53], dehydro-lindestrenolide (**18**) [54], tubipolide B (**19**) [55], atractylenolide II (**20**) [56], 8-*epi*-isogermafurenolide (**21**) [57] and myrrhterpenoid O (**22**). For myrrhterpenoid O, NOESY data were too weak for a confirmation of the configuration. This molecule is probably the correct structure of 2-methoxyfuranoguaia-9-ene-8-one, first published in 1983, as their NMR signals are consistent (Table S5) [15,45].

### 2.2. Triterpenoids

**Figure 9:**
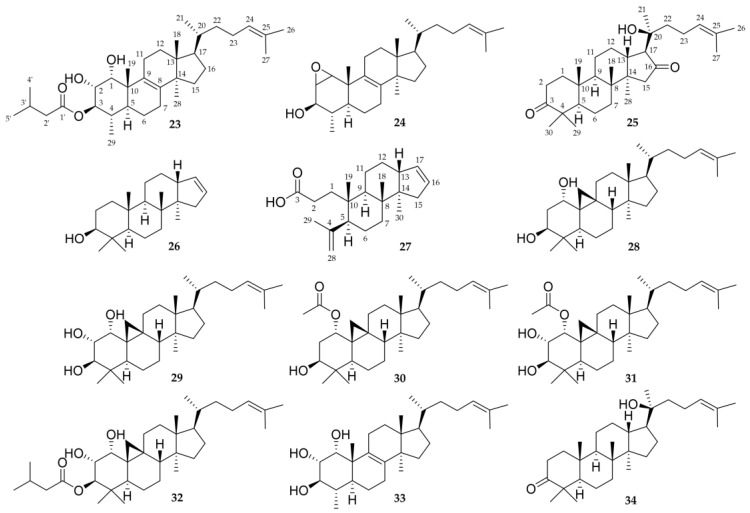
Chemical structures of isolated triterpenoids (**23**–**34**).

The structures of all isolated triterpenoids are presented in Figure 9.

Compound **23** (1.7 mg, white crystals) was assigned to a molecular formula of C_34_H_56_O_4_ based on negative-mode HRESIMS (*m*/*z* 573.4169 [M+HCOO]^−^, calc. for 573.4161). NMR data showed nine methyls (*δ_H_* 0.72 (s, H_3_-18), 1.00 (s, H_3_-19), 0.93 (m, H_3_-21), 1.61 (s, H_3_-26), 1.69 (s, H_3_-27), 0.93 (s, H_3_-28), 0.91 (d, H_3_-29), 0.99 (d, H_3_-4′/H_3_-5′); *δ_C_* 15.7, 18.2, 18.7, 17.6, 25.6, 24.9, 15.0, 22.4, 22.5, respectively), nine sp^3^ methylenes (*δ_H_* 1.32/1.80 (m/m, H_2_-6), 2.04/2.10 (m/m, H_2_-7), 2.12/2.22 (m/m, H_2_-11), 1.75/1.82 (m/m, H_2_-12), 1.21/1.60 (m/m, H_2_-15), 1.33/1.93 (m/m, H_2_-16), 1.05/1.45 (m/m, H_2_-22), 1.86/2.04 (m/m, H_2_-23), 2.27 (m, H_2_-2′); *δ_C_* 20.1, 25.8, 21.5, 30.8, 30.8, 28.1, 36.3, 25.0, 43.7, respectively), eight sp^3^ methines (*δ_H_* 3.94 (brs, H-1), 3.75 (dd, H-2), 4.77 (dd, H-3), 1.63 (m, H-4), 1.62 (m, H-5), 1.52 (m, H-17), 1.40 (m, H-20), 2.16 (m, H-3′); *δ_C_* 75.1, 72.3, 80.0, 34.8, 39.3, 50.3, 36.3, 25.7, respectively), three quaternary carbon (*δ_C_* 41.8 (C-10), 44.6 (C-13), 50.1 (C-14)), four sp^2^ hybridized positions (*δ_H_* 5.10 (dd, H-24); *δ_C_* 138.6 (C-8), 130.0 (C-9), 125.0 (C-24), 130.9 (C-25)) and one carbonyl carbon (*δ_C_* 175.2 (C-1′)) (Table 4).

The NMR data of compound **23** show many similarities to another isolated triterpene (**33**) with one major deviation; H-3 is shifted downfield to 4.77 ppm versus 3.32 ppm in **33**. This effect was also observed in the other *O*-substituted triterpenes **30**–**32**, which also show downfield protons at the substituted hydroxyl groups. The molecular formulas determined by HRESIMS and the additional NMR signals of **30**–**32** confirm a substitution with an isovalerate moiety. Additionally, the position of the substituent is verified by a HMBC signal between H-3 and C-1′ (see also Tables S7 and S8).

**Figure 10:**
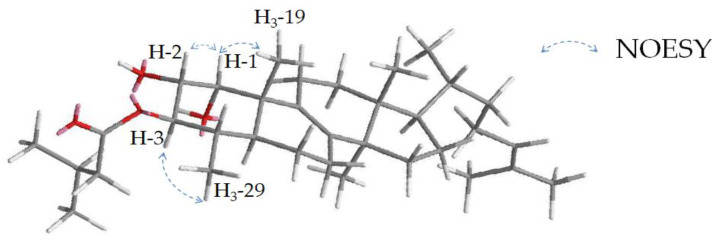
Key NOESY signals of **23** for determination of the relative configuration in the A ring.

The relative configuration of compound **23** in the A ring was determined based on coupling constants and NOESY correlations. A relatively high coupling constant of 9.9 Hz between H-3, -2 and -4 indicates an axial position of these three protons, while the lower coupling constant between H-1 and -2 (^3^*J*_H-1, H-2_ = 2.9 Hz) infers an axial-equatorial coupling and, thus, an equatorial position of H-1. This can be confirmed by NOESY signals between H-1 and -2 as well as -19 and between H-3 and -29 (Figure 10).

Compound **23** can, thus, be assigned a *rel*-(1α,2α,3β) configuration and, reflecting trivial names of known triterpenes, the name 3β-isovaleroyloxy-29-nor-lanost-8,24-diene-1α,2α-diol is suggested for the hitherto-unknown substance.

Compound **24** (2.0 mg) was isolated as white crystals. The positive-mode HRESIMS revealed a molecular formula of C_29_H_46_O_2_ (*m*/*z* 427.3581 [M+H]^+^, calc. 427.3571) and the NMR spectra also showed analogies to other isolated triterpenes: seven methyls (*δ_H_* 0.74 (s, H_3_-18), 1.06 (s, H_3_-19), 0.93 (d, H_3_-21), 1.61 (s, H_3_-26), 1.69 (s, H_3_-27), 0.91 (s, H_3_-28), 0.97 (d, H_3_-29); *δ_C_* 15.7, 17.9, 18.7, 17.6, 25.7, 24.3, 16.3, respectively), eight sp^3^ methylenes (*δ_H_* 1.33/1.74 (m/m, H_2_-6), 2.03 (m, H_2_-7), 2.23/2.29 (m/m, H_2_-11), 1.76/1.82 (m/m, H_2_-12), 1.18/1.57 (m/m, H_2_-15), 1.33/1.93 (m/m, H_2_-16), 1.05/1.45 (m/m, H_2_-22), 1.87/2.04 (m/m, H_2_-23); *δ_C_* 19.2, 24.5, 22.3, 30.7, 30.6, 28.1, 36.3, 24.9, respectively), seven sp^3^ methines (*δ_H_* 3.22 (d, H-1), 3.12 (d, H-2), 3.47 (m, H-3), 1.29 (m, H-4), 1.21 (m, H-5), 1.52 (m, H-17), 1.40 (m, H-20); *δ_C_* 59.6, 27.5, 72.8, 36.1, 35.6, 50.2, 36.3, respectively), three quaternary carbons (*δ_C_* 37.6 (C-10), 44.5 (C-13), 49.9 (C-14)) and four sp^2^ hybridized positions (*δ_H_* 5.10 (dd, H-24), *δ_C_* 136.5 (C-8), 130.3 (C-9), 125.2 (C-24), 131.0 (C-25)) (Table 4)

The NMR data of compound **24** show many similarities to **33**, such as the doublet for H-29 (0.97 ppm) and double bonds between C-8, -9, -24 and -25, indicating a 29-nor-lanost-8,24-diene backbone. In comparison with **33**, two protons and carbons in pos. 1 and 2 are shifted in high field (*δ_H_* 3.22 and 3.12 ppm (**24**) compared to 3.94 and 3.62 ppm (**33**); and *δ_C_* 59.6 and 57.5 ppm (**24**) versus 74.9 and 73.8 ppm (**33**)). These chemical shifts are typical for an epoxide and indicate an epoxidation of compound **24** at C-1/-2, which is confirmed by the HRESIMS formula.

**Figure 11:**
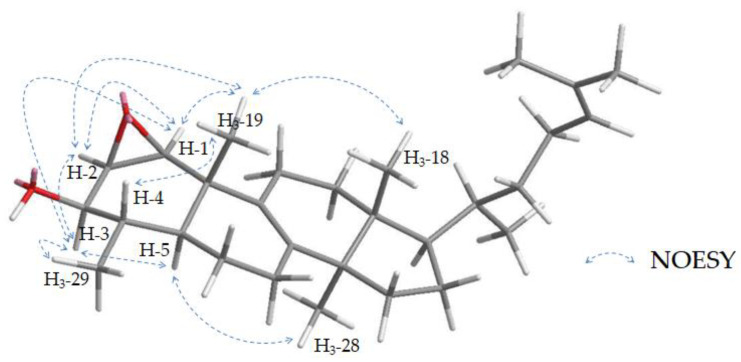
Key NOESY signals of **24** for determination of the relative configuration in the A ring.

Stereochemistry in the A ring was deduced from NOESY signals showing correlations between H_3_-18 and H-19 to confirm a β-configuration of the methyl group at pos. 19. Analogously, a correlation between H-5 and H_3_-28 indicates an α-configuration of H-5. Thus, the orientation at pos. 3 and 4 can be clarified by signals above (H_3_-19, H-4) and below (H-3, -5) the ring (Figure 11).

**Figure 12:**
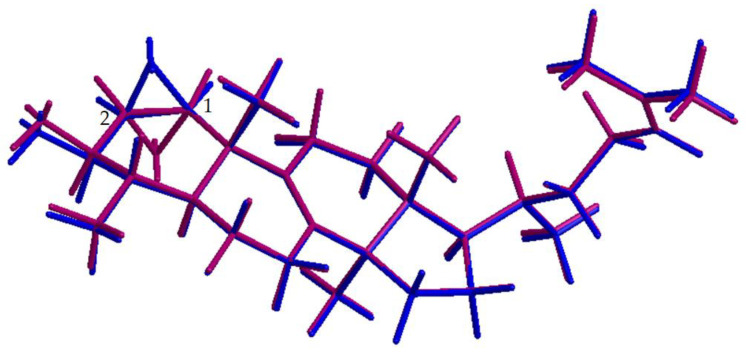
3D model of 29-nor-1β,2β-*cis*-epoxy-lanost-8,24-diene-3β-triol (blue) and 29-nor-1α2α-*cis*-epoxy-lanost-8,24-diene-3β-triol (violet).

Furthermore, the orientation of the epoxy moiety is of interest, because a *cis* or *trans* configuration is possible. The *cis* arrangement is characterized by an equatorial position of the protons H-1 and -2, while in the *trans* configuration, they are axially apart. H-1 and H-2 show a strong NOESY signal, which is atypical for axially located protons. Furthermore, correlations of both protons to groups above (H_3_-19) or below (H-3) the ring can be observed (Figure 12) also suggesting an equatorial orientation. Consequently, a *cis* configuration of the epoxy moiety is postulated. Our methods cannot determine whether the epoxide is in the α or β position, as this does not affect the orientation of the molecule in space, and thus the NMR data (Figure 12). Compound **24** has not been described in the literature, and, therefore, the name 29-nor-1,2-*cis*-epoxy-lanost-8,24-diene-3β-triol is suggested.

Compound **30** (2.0 mg) was obtained as white crystals and assigned a molecular formula of C_32_H_52_O_3_ by HRLIFDIMS (*m*/*z* 484.3900 M^+^, calc. for 484.3911). The NMR data elucidated that the structure and relative configuration of **30** are in accordance with a compound isolated in 1988 by Provan et al. [58]. The complete data set of α-acetoxy-9,19-cyclolanost-24-ene-3β-ol (**30**) is presented here for the first time (Table S7, Figure S3).

Compound **33** (32.7 mg, white crystals) was assigned a molecular formula of C_29_H_48_O_3_ using positive-mode HRESIMS (*m*/*z* 445.3675 [M+H]^+^, calc. for 445.3676) and identified as previously isolated 29-nor-lanost-8,24-diene-1α,2α,3β-triol [58]. The NMR data of this substance, published in 1988, are fragmentary and could now be completed. Additionally, the assignment of some carbons was modified, affecting pos. 1–3, 7–9, 11, 12, 16, 19 and 26–28 (Table S8).

Other triterpenoids were elucidated from their NMR spectra such as *rel*-20*S*-hydroxy-dammar-24-en-3,16-dione (**25**) [15], mansumbinol (**26**) [59], 3,4-seco-mansumbinoic acid (**27**) [59,60], cycloartan-24-ene-1α,3β-diol (**28**) [61], cycloartan-24-ene-1α,2α,3β-triol (**29**) [62,63], α-acetoxycycloartan-24-ene-2α,3β-diol (**31)** [61], 3β-isovaleroyloxycycloartan-24-ene-1α,2α-diol (**32**) [61] and hydroxydammarenone II (**34**) [64,65].

### 2.3. The Cytotoxicity of Selected Compounds against HeLa Cells

For investigation of their biological activity, selected and mainly sesquiterpenoid compounds, which were obtained in sufficient amount and HPLC-DAD purity (>90%) (**4**–**6**, **9**–**11**, **17**, **18**, **20** and **27**), were tested in an ICAM-1 in vitro assay, as described before [66]. In this assay, only a small reduction (less than 20%) of TNF-α dependent ICAM-1 expression in HMEC-1 cells was observed, and, hence, no relevant anti-inflammatory activity could be detected. The results are provided in the Supplementary Material in Figure S14.

Additionally, cytotoxic effects of selected isolates (**2**, **26**–**29**, **31**, **33**, and **34**) on human cervical cancer cells (HeLa) were studied with special attention to the triterpenoids. For this, an MTT viability assay was used and carried out with some modifications according to Mosman [67] monitoring the viability of the cells via their (mitochondrial) reductase activity. The results are presented in relation to the untreated control (u.c.). To exclude the possibility of solvent effects, cells were also treated with the highest used DMSO concentration (negative control).

**Figure 13:**
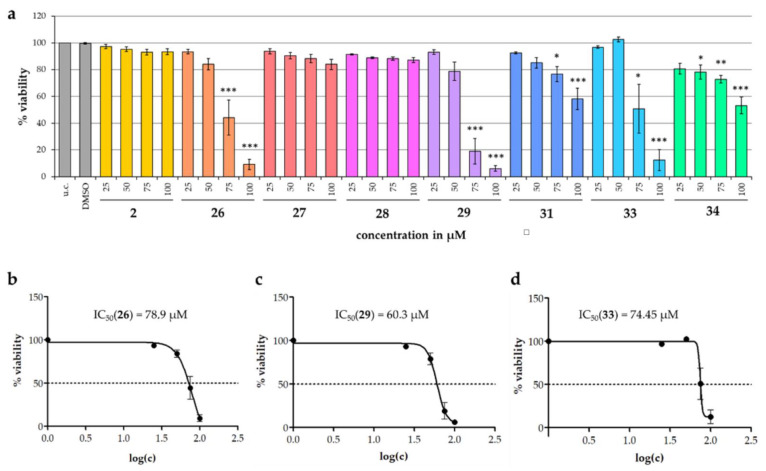
(**a**) Influence of compounds **2**, **26**–**29**, **31**, **33** and **34** on the viability of HeLa cells in the MTT assay. The test was performed with pure medium (u.c.), the highest used concentration of DMSO (0.2%, *v*/*v*) and substance concentrations between 25 and 100 μM. Data are presented as mean +/− SEM; * *p* < 0.05, ** *p* < 0.01, *** *p* < 0.001 vs. u.c. (Tukey-HSD, *n* = 3). Additionally, nonlinear regression curves of compounds **26** (**b**), **29** (**c**) and **33** (**d**) and their corresponding IC_50_ values are presented.

A significant reduction in the viability of HeLa cells could be observed for five of the tested substances: **26**, **29**, **31**, **33** and **34**. Two substances (**31**, **34**) showed weak but significant concentration-dependent effects. The three isolates with the most cytotoxic activity were compounds **26** (mansumbinol), **29** (cycloartan-24-ene-1α,2α,3β-triol) and **33** (29-nor-lanost-8,24-diene- 1α,2α,3β-triol) with IC_50_ values of 78.9, 60.3 and 74.5 µM, respectively (Figure 13).

## 3. Discussion and Conclusions

The presented 34 terpenoids from myrrh are only a small excerpt of its secondary metabolites and illustrate their broad heterogeneity. Sesquiterpenes of six different structural types (germacranes, eudesmanes, seco-eudesmanes, elemanes, cadinane, and guaiane) were found, in addition to one sesquiterpene dimer and triterpenoids of four different scaffolds (mansumbinanes, lanostanes, dammaranes, and cycloartanes). The analysis of the structural richness is crucial for the further development of the standardized quality analytics of the drug, which are still based on a TLC experiment detecting the volatile sesquiterpenes in the European Pharmacopoeia [68]. The examination of the composition is also the basis for finding the molecular targets of the traditionally used drug, which are still unknown.

**Figure 14:**
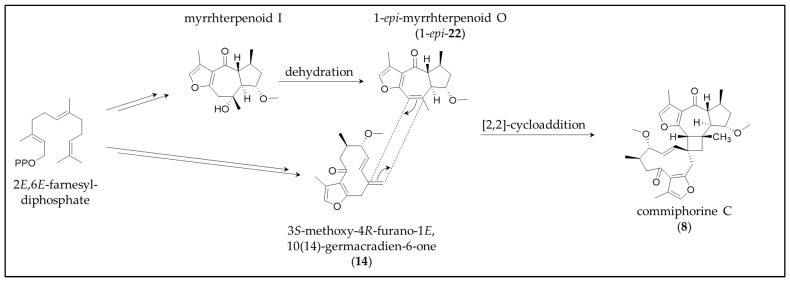
Plausible biosynthetic pathway for commiphorine C (**8**) by 2,2-cycloaddition of two monomer sesquiterpenes according to Dong et al. [21].

Concerning the sesquiterpene dimer (**8**), the accompanying isolation of its monomers with a double bond at the linking position strongly supports Dong et al.’s (2019) suggestion of a 2,2-cycloaddition as biosynthetic pathway (Figure 14) [21].

To test the anti-inflammatory activity of the isolated molecules, the ICAM-1 expression on human endothelial cells was chosen as a pathway. Interestingly, one of the active compounds found in earlier work [18] only differs in a missing double bond from as inactive analysed compounds **18** (position C-8,9) or an additional one from **20** (position C-1,2). The negative results of all the tested substances indicate that the proven anti-inflammatory activity of myrrh is either caused by other compounds [18] or triggered via another pathway. Investigation of the molecular mechanisms of action, therefore, remains an interesting field for further research.

The observed cytotoxic effect of compounds **26**, **29** and **33** on HeLa cells provide a first hint of cytotoxic activity of these myrrh triterpenoids against cancer cells. For a more comprehensive and robust investigation of cytotoxicity a broader set of triterpenoids as well as a second quantitative analytical method for purity control, must be included. Interestingly, an A ring cleavage of mansumbinol (**26**) resulting in a carboxylic acid and an isopropenyl partial structure led to a complete loss of activity for compound **27**. Compound **29**, with three hydroxyl groups at C-1-3, showed significant cytotoxicity, whereas similar substances with a different substitution pattern in the A ring, as in **28** or **31**, were less potent. This was also observed for **33**, which is a lanostane-type triterpene but corresponds to **29** with the A ring substitution. Thus, it can be hypothesized that the 1,2,3-trihydroxy substructure in the A ring contributes to a cytotoxic effect, whereas the type of triterpene skeleton has less influence on the activity.

To our knowledge, this is the first time that myrrh triterpenes are reported to have cytotoxic effects against HeLa cells. Previous investigations occasionally describe the cytotoxic activity of myrrh oil or single myrrh sesquiterpenes on both normal and cancer cells, but activity is often selective for the former [33,69]. Further cell viability experiments with other tumour and noncancer cell lines will be necessary to characterize their activity better, especially regarding possibly selective anticancer activity or general cytotoxicity.

## 4. Materials and Methods

### 4.1. Chemicals

Extraction was performed with ethanol of technical quality from CSC Jäcklechemie (Nuremberg, Germany) after purification by evaporation. Methanol, n-heptane, ethyl acetate, dichloromethane, diethyl ether and toluene (all per analysis quality; p.a.) were purchased from Fisher Scientific (Hampton, New Hampshire, USA). Anisaldehyde (4-methoxybenzaldehyde for synthesis), sulfuric acid (95–97%, p.a.) and acetonitrile (HPLC-grade) were obtained from Merck Chemicals (Darmstadt, Germany), and n-hexane (p.a.), chloroform-*d* (99.8%), Dulbecco’s Phosphate-Buffered Saline and Minimum Essential Medium Eagle were from Sigma-Aldrich (St. Louis, Missouri, USA). Formic acid (p.a.) and DMSO (p.a. ≥ 99.5% for molecular biology) were provided by Carl Roth (Karlsruhe, Germany). Foetal bovine serum (FBS) Superior, L-glutamine, nonessential amino acids and trypsin/EDTA for cell culturing were purchased from Biochrom (Berlin, Germany), and 3-(4,5-dimethylthiazol-2-yl)-2,5-diphenyltetrazoliumbromide (MTT) as well as sodiumdodecylsulfate (SDS) from Sigma-Aldrich (Taufkirchen, Germany).

### 4.2. Plant Material and Extraction

Powdered myrrh resin of *C. myrrha* (Myrrha, Ph. Eur. 2016) was obtained from Lomapharm^®^ (lot NM0160, Rudolf Lohmann GmbH KG, Emmerthal, Germany). 3 kg of powdered resin was extracted by maceration and percolation with ethanol 96% (*v*/*v*) as described before [18].

### 4.3. Isolation

The isolation process was carried out mostly as published [18] and is, thus, roughly summarized and complemented with the new procedures.

#### 4.3.1. Liquid-Liquid Partition

Portions of ethanolic extract were solved in methanol and partitioned with n-heptane in a separatory funnel to gain a methanol (MeOH, 328.13 g) and an n-heptane-soluble fraction (HEP, 69.99 g).

#### 4.3.2. Solid Phase Extraction: MeOH Fraction

To further divide the methanol fraction, solid-phase extraction by silica gel (Geduran Si 60 (0.063–0.200 mm), Merck Chemicals, Darmstadt, Germany) was performed. Therefore, 150 g of MeOH fraction was mortared with silica gel and submitted dryly in portions on wet-packed columns. The first elution step with ethyl acetate resulted in fraction M1 (107.0 g) and the second step with methanol in M2 (42.3 g).

#### 4.3.3. Silica Flash Chromatography 1

##### HEP Fraction

Using silica flash chromatography (Spot flash system (Armen Instrument, Paris, France), SVP D40 silica column (13 × 4 cm, SI60 15–40 μm, 90 g, Götec Labortechnik GmbH, Bickenbach, Germany) with an n-hexane (A)/ethyl acetate (B) gradient (30 mL/min; 0–30 min 5% B, 30-90 min 5–20% B, 90–110 min 20–100% B, 110–140 min 100% B), the HEP fraction was partitioned in ten fractions F1-10. F5 (1301-1875 mL, 2.5 g), F6 (1876–2450 mL, 1.1 g), F7 (2451–2950 mL, 1.6 g), and F9 (3201–3550 mL, 1.9 g) were further investigated (retention volume and weight).

##### MeOH Fraction M1

Using the same flash system, 51 g of M1 was separated into ten subfractions M1.1-1.10 using a silica column (100 × 3.6 cm, SI60 0.063–0.2 mm, approx. 520 g, Merck KGaA, Darmstadt, Germany) and an n-hexane (A)/ethyl acetate (B)/methanol (C) gradient (10 mL/min; 0–5 min 80% A/20% B, 5-245 min 20-100% B, 245-395 min 100% B, 395–491 min 0–100% C, 491–585 min 100% C). Fraction M1.2 (1150-1575 mL retention volume, 0.6 g weight) was further investigated.

#### 4.3.4. Centrifugal Partition Chromatography (CPC)

A Spot centrifugal partition chromatography (CPC) device with a 250 mL rotor (Armen Instrument, Paris, France), a 510 HPLC pump (Waters GmbH, Eschborn, Germany) and a 2111 Multirac Fraction Collector (LKB-Produkter AB, Bromma, Sweden) was used for the separation of fractions F5-7, F9 and M1.2. The separation was performed at a rotation speed of 1000 rpm and a flow rate of 5 mL/min, with a solvent system consisting of n-hexane, acetonitrile and methanol (40/25/10 *v*/*v*/*v*) [70], and was executed in two stages. First, the lower phase (LP) was used as a stationary phase in ascending mode (ASC) for 800 mL for F5-7/F9 or 925 mL for M1.2, respectively. Second, phases were switched, and the process was conducted in descending mode (DSC) for another 200 mL or 275 mL, respectively. Subsequently, subfractions (F5C1-6, F6C1-8, F7C1-8, F9C1-4, and M1.2C1-7) were formed, of which the following were processed further: fraction (mode, retention volume, and weight): F5C2 (ASC 181–280 mL, 70.6 mg), F5C5 (DSC 21–135 mL, 641.3 mg), F6C3 (ASC 191–275 mL, 20.6 mg), F6C4 (ASC 276–385 mL, 24.5 mg), F6C5 (ASC 386–520 mL, 46.6 mg), F6C7 (DSC 16–190 mL, 386.2 mg), F7C2 (ASC 196–480 mL, 43.4 mg), F7C3 (ASC 481–720 mL, 41.3 mg), F7C7 (DSC 11–50 mL, 508.4 mg), F9C2 (ASC 364–450 mL, 20.5 mg), F9C3 (ASC 451–585 mL, 118.1 mg), F9C4 (ASC 586–800 mL, 77.6 mg), M1.2C3 (ASC 311–620 mL, 107.0 mg), M1.2C6 (DSC 986–1060 mL, 129.7 mg) and M1.2C7 (DSC, 1061–1200 mL, 73.5 mg).

#### 4.3.5. Silica Flash Chromatography 2

The fractions F6C7 and F7C7 were subjected to a second flash separation. A SVF D26 silica column (9 × 2.8 cm, SI60 15–40 μm, 30 g, Götec Labortechnik GmbH, Bickenbach, Germany) was used with a flow rate of 15 mL/min and a dichloromethane (A)/ethyl acetate (B) gradient (0–60 min 1–6% B, 60–90 min 6–15% B, 90–92 min 15–100% B, 92–110 min 100% B). Subfractions (F6C7F1-5 and F7C7F1-5) were combined according to thin-layer chromatography (TLC) control, and some were used for further isolation: fraction (retention volume and weight): F6C7F3 (256–510 mL, 64.5 mg), F7C7F1 (1–390 mL, 90.2 mg), F7C7F2 (391–465 mL, 40.4 mg) and F7C7F3 (466–660 mL, 52.6 mg).

#### 4.3.6. Thin-Layer Chromatography (TLC)

For flash chromatography and CPC, fractions were checked and combined by TLC control on silica gel 60 F254 (Merck, Darmstadt, Germany) with a mobile phase consisting of toluene and ethyl acetate 95/5, 80/20 or 50/50 (*v*/*v*) for HEP fractions and M1.2C1-7 or hexane, ethyl acetate and methanol 15/80/5 (*v*/*v*/*v*) for M1.1-1.10. Derivatization was conducted with anisaldehyde reagent R (Ph. Eur.) and documented by a Camag TLC visualizer (Camag AG, Muttenz, Switzerland).

#### 4.3.7. Preparative HPLC

For the isolation of pure compounds, a preparative high-performance liquid chromatography (HPLC) device equipped with a 1260 Infinity binary pump, a 1260 Infinity manual injector, a 1260 Infinity fraction collector, a 1260 Infinity diode array detector (all Agilent Technologies, Santa Clara, USA) and a Kinetex^®^ column (Biphenyl, 100 Å, 5 μm, 21.2 × 250 mm, Phenomenex, Aschaffenburg, Germany) at a flow rate of 21 mL/min or—for fractions M1.2C6 and M1.2C7—a Nucleodur^TM^ C18 Isis column (RP18, 5 µm, 10 × 250 mm, Macherey-Nagel, Düren, Germany) at a flow rate of 5 mL/min was used. Separation was achieved by gradients consisting of acetonitrile (A)/water (B) and peaks were detected at 200 nm. After the elimination of acetonitrile via evaporation, the water fractions were partitioned four times with diethyl ether or ethyl acetate (M1.2C6 and M1.2C7) and organic phases were dried under a nitrogen stream. The gradients used for the separation can be found in Table 5.

### 4.4. NMR

NMR spectra (1D-^1^H, 1D-^13^C as well as 2D-^1^H,^13^C HSQC, ^1^H,^13^C HMBC, ^1^H,^1^H COSY, and ^1^H,^1^H NOESY) were recorded in CDCl_3_ in 507-HP-8 NMR tubes (Norell Inc, Morganton, USA) on an AVANCE III 600 NMR equipped with a 5 mm TBI CryoProbe (^1^H NMR 600.25 MHz, ^13^C NMR 150.95 MHz, 298 K) or an AVANCE III HD NMR (^1^H NMR 400.13 MHz, ^13^C NMR 100.63 MHz, 298 K) (Bruker Corporation, Billerica, MA, USA). For structure elucidation, the data were subsequently processed with TopSpin 3.1 or 3.5.b.91 pl 7 (Bruker Corporation).

### 4.5. UHPLC-MS

Isolates were analysed using ultra-high-performance liquid chromatography coupled to mass spectrometry (UHPLC-MS) (1290 Infinity UHPLC and Q-TOF 6540 UHD, Agilent Technologies, Santa Clara, USA) on a ZORBAX Eclipse column (XDB-C18 RRHD, 2.1 × 100 mm, 1.8 µm, Agilent Technologies) with the following gradient: solvent A (water with 0.1% formic acid); solvent B (acetonitrile with 0.1% formic acid); 0–10 min, 20-98% B; 10–12 min, 98% B; 12–12.1 min, 98-20% B; 12.1–13.5 min, 20% B; flow: 0.5 mL/min; column temperature: 50 °C; and injection volume: 1 µL. MS analysis was subsequently performed by electrospray ionization (ESI) in positive and negative mode or liquid injection field desorption ionisation (LIFDI) as an even softer ionisation method for compound **30** to avoid the cleavage of the ester.

### 4.6. Optical Methods

For further characterization of the isolates, optical data were gathered by using solutions in methanol. Specific optical rotation was measured by an UniPol L 1000 polarimeter (Schmidt + Haensch GmbH & Co., Berlin, Germany) using a microtube (50 mm, 550 μL) at 589 nm. UV-spectra were recorded by a Cary 50 Scan UV-spectrophotometer (Varian Deutschland GmbH, Darmstadt, Germany) in a quartz cuvette (QS, 1.0 cm, Hellma GmbH & Co. KG, Müllheim, Germany) in a range of 200–800 nm. Additionally, CD spectra were measured on a J-715 spectropolarimeter (JASCO Deutschland GmbH, Gross-Umstadt, Germany) with a 0.1 cm quartz cuvette (Type: 100-QSQ, Hellma GmbH & Co. KG). Thereby, ten scans were recorded at 22 °C from 190 to 300 or 400 nm with a scanning rate of 50, 100 or 200 nm/min in 0.5 nm steps and the Savitzky–Golay algorithm was used for spectra smoothing (convolution width: 15).

### 4.7. Simulation of ECD Spectra

Molecular models of commiphorine A and compound **8** (commiphorine C) were generated with the Molecular Operations Environment (MOE, v. 2020.09, Chemical Computing Group, Montreal, Canada) using the MMFF94x force field. After a conformational search with the low mode dynamics (LMD) method, the lowest energy conformers were energy-minimized with the semiempirical method PM3. In each case, the lowest energy conformer was used for the spectra simulation. The structure was exported to Gaussian (v. Gaussian03W, Gaussian Inc., Pittsburgh, Pa., U.S.A.) and fully minimized by density functional theory (DFT) with the B3LYP density functional and the 6−31G(d,p) basis set. The minimized structures were then subjected to a time-dependent DFT (TDDFT) calculation using the same functional and basis set, which yielded the electronic transition vectors determining the ECD spectra. Typically, the first (i.e., lowest in frequency) 12–18 electronic transitions were computed. The Gaussian output for electronic transition energies (E in eV) and rotator strengths (R, dipole length in cgs) was used to simulate ECD spectra by applying a Gaussian shape function with a band width at 1/e height σ = 0.15 eV. The resulting spectra shown in Figure 8 and Figure 9 were scaled by factor 0.5 and blue-shifted by 0.15 eV.

The structures of three conformers of 1,5-*cis*-configured “commiphorine A” were built directly from the atomic coordinates published by the authors of the previous study [21] in their supplementary file. The ECD spectra of these models were simulated as described above. There was no significant difference between the three spectra, and so only the spectrum of structure 1a1 [21] is shown in Figure S1.

### 4.8. Purity

The purity of isolates was determined through HPLC-DAD analysis (190-400 nm for the HEP fraction or 200–400 nm for the MeOH fraction) on an Elite LaChrom system consisting of an autosampler L-2200, a pump L-2130, a column oven L-2350, a diode array detector L-2455 (all Hitachi, Tokyo, Japan) and a Kinetex^®^ biphenyl column (100 Å, 5 µm, 4.6 × 250 mm, Phenomenex, Aschaffenburg, Germany) or a Nucleodur^TM^ C18 Isis column (RP18, 5 µm, 4.6 × 250 mm, Macherey-Nagel, Düren, Germany). For analyses, gradients described in 0 were conducted at a flow rate of 1 mL/min and an injection volume of 5 or 10 µL (acetonitrile, 1 mg/mL). Thus, the purity was calculated as the proportion of the integral of the main peak in the chromatogram using the maxplot adjusted by a blank (EZChrom Elite 3.1.7, Hitachi).

### 4.9. Isolated Compounds

As software for drawing the chemical structures, ChemDraw Professional 20.0 (PerkinElmer Informatics Inc., Waltham, MA, USA) was used. Three-dimensional models were generated with Chem3D Ultra 20.0 from the same company.

#### 4.9.1. 9,10-Seco-isolindestrenolide (**1**)

1.0 mg, colourless oil; ${\left[\alpha \right]}_{D}^{22}$ +238 (*c* 1.11, MeOH); UV (MeOH) *λ*_max_ (log *ε*): 203 nm (3.97); CD: Figure S2; ^1^H and ^13^C NMR data (CDCl_3_, 600 and 150 MHz, respectively) in Table 1 and Figure S4; HRESIMS *m*/*z* 231.1383 [M+H]^+^ (calc. for C_15_H_19_O_2_, 231.1380); purity (according 4.8.) 89.9%.

#### 4.9.2. 9-Oxo-9,10-seco-isolindestrene (**2**)

4.1 mg, yellow oil; UV (MeOH) *λ*_max_ (log *ε*): 286 nm (3.43); ^1^H and ^13^C NMR data (CDCl_3_, 600 and 150 MHz, respectively) in Table 1 and Figure S5; HRESIMS *m*/*z* 229.1226 [M+H]^+^ (calc. for C_15_H_17_O_2_, 229.1223); purity (according 4.8.) 97.6%.

#### 4.9.3. *rel*-8S-Acetyloxy-7R-hydroxy-5R,10R-β-elemene (**3**)

1.2 mg, colourless oil; ${\left[\alpha \right]}_{D}^{25}$ +36 (*c* 1.28, MeOH); UV (MeOH) λ_max_ (log *ε*): 202 nm (3.80), 276 nm (3.57); CD: Figure S2; ^1^H and ^13^C NMR data (CDCl_3_, 600 and 150 MHz, respectively) in Table 2 and Figure S6; HRESIMS *m*/*z* 279.1950 [M+H]^+^ (calc. for C_17_H_27_O_3_, 279.1955); purity (according 4.8.) 88.3%.

##### 4.9.4. Commiterpene E (**4**)

0.8 mg, colourless oil; ${\left[\alpha \right]}_{D}^{25}$ +18 (*c* 0.90, MeOH); UV (MeOH) *λ*_max_ (log *ε*): 203 nm (4.26); CD: Figure S2; ^1^H and ^13^C NMR data (CDCl_3_, 600 and 150 MHz, respectively) in Table 2 and Figure S7; HRESIMS *m*/*z* 261.1490 [M+H]^+^ (calc. for C_16_H_21_O_3_, 261.1485); purity (according 4.8.) 93.5%.

##### 4.9.5. 2β-Methoxyglechomanolide (**5**)

2.9 mg, white crystals; ${\left[\alpha \right]}_{D}^{25}$ +53; UV (*c* 1.60, MeOH); *λ*_max_ (log *ε*): 218 nm (3.98); CD: Figure S2; ^1^H and ^13^C NMR data (CDCl_3_, 600 and 150 MHz, respectively) in Table 2 and Figure S8; HRESIMS *m*/*z* 263.1646 [M+H]^+^ (calc. for C_16_H_23_O_3_, 263.1642); purity (according 4.8.) 90.9%.

##### 4.9.6. 8-*epi*-2β-Methoxyglechomanolide (**6**)

6.5 mg, white crystals; ${\left[\alpha \right]}_{D}^{25}$ −69; UV (*c* 2.23, MeOH); *λ*_max_ (log *ε*): 215 nm (4.17); CD: Figure S2; ^1^H and ^13^C NMR data (CDCl_3_, 400 and 100 MHz, respectively) in Table 2 and Figure S9; HRESIMS *m*/*z* 263.1646 [M+H]^+^ (calc. for C_16_H_23_O_3_, 263.1642); purity (according 4.8.) 96.9%.

##### 4.9.7. Dehydroisolindestrenolide (**7**)

1.8 mg, colourless oil; ${\left[\alpha \right]}_{D}^{24}$ +34 (*c* 1.94, MeOH); UV (MeOH) *λ*_max_ (log *ε*): 202 nm (3.94), 275 nm (3.79); CD: Figure S2; ^1^H and ^13^C NMR data (CDCl_3_, 600 and 150 MHz, respectively) in Table 3 and Figure S10; HRESIMS *m*/*z* 229.1228 [M+H]^+^ (calc. for C_15_H_17_O_2_, 229.1223); purity (according 4.8.) 89.5%.

##### 4.9.8. Commiphorine C (**8**)

0.9 mg, white solid; ${\left[\alpha \right]}_{D}^{27}$ -33 (*c* 0.97, MeOH); UV (MeOH) *λ*_max_ (log *ε*): 202 nm (4.06), 277 nm (3.48); CD: Figure S2; ^1^H and ^13^C NMR data (CDCl_3_, 600 and 150 MHz, respectively) in Table 3 and Figure S11; HRESIMS *m*/*z* 521.2904 [M+H]^+^ (calc. for C_32_H_41_O_6_, 521.2898); purity (according 4.8.) 85.3%.

##### 4.9.9. Glechomanolide (**9**)

5.7 mg, white crystals; ${\left[\alpha \right]}_{D}^{25}$ +6 (*c* 1.84, MeOH); UV (MeOH) *λ*_max_ (log *ε*): 214 nm (4.10); CD: Figure S2; ^1^H and ^13^C NMR data (CDCl_3_, 400 and 100 MHz, respectively) in Table S1; HRESIMS *m*/*z* 233.1540 [M+H]^+^ (calc. for C_15_H_21_O_2_, 233.1536); purity (according 4.8.) 97.2%.

##### 4.9.10. 2β-Acetyloxyglechomanolide (**10**)

10.9 mg, white crystals; ${\left[\alpha \right]}_{D}^{25}$ +26 (*c* 2.31, MeOH); UV (MeOH) *λ*_max_ (log *ε*): 216 nm (4.15), 285 nm (2.91); CD: Figure S2; ^1^H and ^13^C NMR data (CDCl_3_, 400 and 100 MHz, respectively) in Table S1; HRESIMS *m*/*z* 291.1595 [M+H]^+^ (calc. for C_17_H_23_O_4_, 291.1591); purity (according 4.8.) 95.4%.

##### 4.9.11. 8-*epi*-2β-Acetyloxyglechomanolide (**11**)

11.3 mg, white crystals; ${\left[\alpha \right]}_{D}^{25}$ +26 (*c* 2.27, MeOH); UV (MeOH) *λ*_max_ (log *ε*): 214 nm (4.20); CD: Figure S2; ^1^H and ^13^C NMR data (CDCl_3_, 400 and 100 MHz, respectively) in Table S1; HRESIMS *m*/*z* 291.1598 [M+H]^+^ (calc. for C_17_H_23_O_4_, 291.1591); purity (according 4.8.) 92.0%.

##### 4.9.12. 2α-Methoxy-6-oxogermacra-1(10),7(11)-dien-8,12-olide (**12**)

1.1 mg, white crystals; ${\left[\alpha \right]}_{D}^{25}$ +107 (*c* 1.12, MeOH); UV (MeOH) *λ*_max_ (log *ε*): 230 nm (3.76); CD: Figure S2; ^1^H and ^13^C NMR data (CDCl_3_, 600 and 150 MHz, respectively) in Table S2; HRESIMS *m*/*z* 279.1598 [M+H]^+^ (calc. for C_16_H_23_O_4_, 279.1591); purity (according 4.8.) 89.3%.

##### 4.9.13. 1(10)*Z*,4*E*-Isofuranodienone (**13**)

1.6 mg (including **22**), colourless oil; ^1^H and ^13^C NMR data (CDCl_3_, 600 and 150 MHz, respectively) in Table S2; HRESIMS *m*/*z* 231.1381 [M+H]^+^ (calc. for C_15_H_19_O_2_, 231.1380); purity (according 4.8.) 77.3% (including **22**).

##### 4.9.14. 3*S*-Methoxy-4*R*-furano-1*E*,10(14)-germacradien-6-one (**14**)

6.6 mg, colourless oil; ${\left[\alpha \right]}_{D}^{23}$ +43 (*c* 2.20, MeOH); UV (MeOH) *λ*_max_ (log *ε*): 216 nm (3.97); CD: Figure S2; ^1^H and ^13^C NMR data (CDCl_3_, 400 and 100 MHz, respectively) in Table S2; HRESIMS *m*/*z* 261.1486 [M+H]^+^ (calc. for C_16_H_21_O_3_, 261.1485); purity (according 4.8.) 92.7%.

##### 4.9.15. 2-Methoxy-5-acetoxyfuranogermacr-1(10)-en-6-one (**15**)

9.2 mg, white crystals; ${\left[\alpha \right]}_{D}^{25}$ −21 (*c* 2.22, MeOH); UV (MeOH) *λ*_max_ (log *ε*): 207 nm (4.19), 284 nm (3.27); CD: Figure S2; ^1^H and ^13^C NMR data (CDCl_3_, 400 and 100 MHz, respectively) in Table S3; HRESIMS *m*/*z* 321.1699 [M+H]^+^ (calc. for C_18_H_25_O_5_, 321.1697); purity (according 4.8.) 98.5%.

##### 4.9.16. *rel*-2*R*-Methoxy-5*S*-acetoxy-4*R*-furanogermacr-1(10)*Z*-en-6-one (**16**)

1.8 mg, colourless oil; ${\left[\alpha \right]}_{D}^{25}$ +56 (*c* 1.80, MeOH); UV (MeOH) *λ*_max_ (log *ε*): 275 nm (3.36); CD: Figure S2; ^1^H and ^13^C NMR data (CDCl_3_, 600 and 150 MHz, respectively) in Table S3; HRESIMS *m*/*z* 321.1702 [M+H]^+^ (calc. for C_18_H_25_O_5_, 321.1697); purity (according 4.8.) 92.2%.

##### 4.9.17. (1*S*,4*S*,5*S*,10*S*)-Germacron-1(10),4-diepoxide (**17**)

3.6 mg, white crystals; ${\left[\alpha \right]}_{D}^{25}$ +3 (*c* 1.80, MeOH); UV (MeOH) *λ*_max_ (log *ε*): 248 nm (3.50); CD: Figure S2; ^1^H and ^13^C NMR data (CDCl_3_, 600 and 150 MHz, respectively) in Table S3; HRESIMS *m*/*z* 251.1647 [M+H]^+^ (calc. for C_15_H_23_O_3_, 251.1642); purity (according 4.8.) 88.2%.

##### 4.9.18. Dehydrolindestrenolide (**18**)

1.9 mg, colourless oil; ${\left[\alpha \right]}_{D}^{23}$ +60 (*c* 1.86, MeOH); UV (MeOH) *λ*_max_ (log *ε*): 202 nm (3.96), 204 nm (3.91), 276 nm (3.74); CD: Figure S3; ^1^H and ^13^C NMR data (CDCl_3_, 600 and 150 MHz, respectively) in Table S4; HRESIMS *m*/*z* 229.1225 [M+H]^+^ (calc. for C_15_H_17_O_2_, 229.1223); purity (according 4.8.) 89.5%.

##### 4.9.19. Tubipolide B (**19**)

2.2 mg (including **21**), colourless oil; ^1^H and ^13^C NMR data (CDCl_3_, 600 and 150 MHz, respectively) in Table S4; HRESIMS *m*/*z* 231.1380 [M+H]^+^ (calc. for C_15_H_19_O_2_, 231.1380); purity (according 4.8.) 93.1% (including **21**).

##### 4.9.20. Atractylenolide II (**20**)

1.2 mg, colourless oil; ${\left[\alpha \right]}_{D}^{25}$ +16 (*c* 1.31, MeOH); UV (MeOH) *λ*_max_ (log *ε*): 202 nm (3.52), 205 nm (3.51), 221 nm (3.94); CD: Figure S3; ^1^H and ^13^C NMR data (CDCl_3_, 600 and 150 MHz, respectively) in Table S4; HRESIMS *m*/*z* 233.1545 [M+H]^+^ (calc. for C_15_H_21_O_2_, 233.1536); purity (according 4.8.) 96.5%.

##### 4.9.21. 8-*epi*-Isogermafurenolide (**21**)

2.2 mg (including **19**), colourless oil; ^1^H and ^13^C NMR data (CDCl_3_, 600 and 150 MHz, respectively) in Table S5; HRESIMS *m*/*z* 233.1536 [M+H]^+^ (calc. for C_15_H_21_O_2_, 233.1536); purity (according 4.8.) 93.1% (including **19**).

##### 4.9.22. Myrrhterpenoid O (**22**)

1.6 mg (including **13**), colourless oil; ${\left[\alpha \right]}_{D}^{23}$ +152 (*c* about 0.66, MeOH); CD: Figure S3; ^1^H and ^13^C NMR data (CDCl_3_, 600 and 150 MHz, respectively) in Table S5; HRESIMS *m*/*z* 261.1490 [M+H]^+^ (calc. for C_16_H_21_O_3_, 261.1485); purity (according 4.8.) 77.3% (including **13**).

##### 4.9.23. 3β-Isovaleroyloxy-29-nor-lanost-8,24-diene-1α,2α-diol (**23**)

1.7 mg, white crystals; ${\left[\alpha \right]}_{D}^{25}$ +28 (*c* 1.70, MeOH); CD: Figure S3; ^1^H and ^13^C NMR data (CDCl_3_, 600 and 150 MHz, respectively) in Table 4 and Figure S12; HRESIMS *m*/*z* 573.4169 [M+HCOO]^−^ (calc. for C_35_H_57_O_6_, 573.4161); purity (according 4.8.) 60.9%.

##### 4.9.24. 29-Nor-1,2-*cis*-epoxylanost-8,24-diene-3β-triol (**24**)

2.0 mg, white crystals; ${\left[\alpha \right]}_{D}^{25}$ +32 (*c* 2.00, MeOH); CD: Figure S3; ^1^H and ^13^C NMR data (CDCl_3_, 600 and 150 MHz, respectively) in Table 4 and Figure S13; HRESIMS *m*/*z* 427.3581 [M+H]^+^ (calc. for C_29_H_47_O_2_, 427.3571); purity (according 4.8.) 83.2%.

##### 4.9.25. *rel*-20*S*-Hydroxydammar-24-en-3,16-dione (**25**)

6.2 mg, white crystals; ${\left[\alpha \right]}_{D}^{25}$ +1 (*c* 2.05, MeOH); CD: Figure S3; ^1^H and ^13^C NMR data (CDCl_3_, 600 and 150 MHz, respectively) in Table S6; HRESIMS *m*/*z* 501.3592 [M+HCOO]^−^ (calc. for C_31_H_49_O_5_, 501.3585); purity (according 4.8.) 71.6%.

##### 4.9.26. Mansumbinol (**26**)

16.1 mg, white needles; ${\left[\alpha \right]}_{D}^{25}$ −19 (*c* 2.64, MeOH); CD: Figure S3; ^1^H and ^13^C NMR data (CDCl_3_, 400 and 100 MHz, respectively) in Table S6; HRESIMS *m*/*z* 334.3105 [M+NH_4_]^+^ (calc. for C_22_H_40_ON, 334.3104); purity (according 4.8.) 83.1%.

##### 4.9.27. 3,4-Seco-mansumbinoic acid (**27**)

9.4 mg, white crystals; ${\left[\alpha \right]}_{D}^{25}$ +1 (*c* 1.85, MeOH); CD: Figure S3; ^1^H and ^13^C NMR data (CDCl_3_, 400 and 100 MHz, respectively) in Table S6; HRESIMS *m*/*z* 329.2491 [M-H]^−^ (calc. for C_22_H_33_O_2_, 329.2486); purity (according 4.8) 93.4%.

##### 4.9.28. Cycloartan-24-ene-1α,3β-diol (**28**)

6.4 mg, white crystals; ${\left[\alpha \right]}_{D}^{25}$ +64 (*c* 2.02, MeOH); CD: Figure S3; ^1^H and ^13^C NMR data (CDCl_3_, 400 and 100 MHz, respectively) in Table S7; HRESIMS *m*/*z* 487.3797 [M+HCOO]^−^ (calc. for C_31_H_51_O_4_, 487.3793); purity (according 4.8.) 90.5%.

##### 4.9.29. Cycloartan-24-ene-1α,2α,3β-triol (**29**)

48.4 mg, white crystals; ${\left[\alpha \right]}_{D}^{25}$ +38 (*c* 1.74, MeOH); CD: Figure S3; ^1^H and ^13^C NMR data (CDCl_3_, 400 and 100 MHz, respectively) in Table S7; HRESIMS *m*/*z* 476.4100 [M+NH_4_]^+^ (calc. for C_30_H_54_O_3_N, 476.4098); purity (according 4.8.) 90.6%.

##### 4.9.30. 1α-Acetoxy-9,19-cyclolanost-24-ene-3β-ol (**30**)

2.0 mg, white crystals; ${\left[\alpha \right]}_{D}^{25}$ +46 (*c* 2.07, MeOH); CD: Figure S3; ^1^H and ^13^C NMR data (CDCl_3_, 600 and 150 MHz, respectively) in Table S7; HRLIFDIMS *m*/*z* 484.3900 M^●+^ (calc. for C_32_H_52_O_3_, 484.3911); purity (according 4.8.) 68.8%.

##### 4.9.31. 1α-Acetoxycycloartan-24-ene-2α,3β-diol (**31**)

6.0 mg, white crystals; ${\left[\alpha \right]}_{D}^{25}$ +30 (*c* 1.92, MeOH); CD: Figure S3; ^1^H and ^13^C NMR data (CDCl_3_, 400 and 100 MHz, respectively) in Table S8; HRESIMS *m*/*z* 523.3751 [M+Na]^+^ (calc. for C_32_H_52_O_4_Na, 523.3758); purity (according 4.8.) 89.2%.

##### 4.9.32. 3β-Isovaleroyloxycycloartan-24-ene-1α,2α-diol (**32**)

2.4 mg, white crystals; ${\left[\alpha \right]}_{D}^{25}$ +25 (*c* 2.58, MeOH); CD: Figure S3; ^1^H and ^13^C NMR data (CDCl_3_, 600 and 150 MHz, respectively) in Table S8; HRESIMS *m*/*z* 565.4233 [M+Na]^+^ (calc. for C_33_H_58_O_4_Na, 565.4227); purity (according 4.8.) 54.9%.

##### 4.9.33. 29-Nor-lanost-8,24-diene-lα,2α,3β-triol (**33**)

32.7 mg, white crystals; ${\left[\alpha \right]}_{D}^{25}$ +83 (*c* 1.08, MeOH); CD: Figure S3; ^1^H and ^13^C NMR data (CDCl_3_, 400 and 100 MHz, respectively) in Table S8; HRESIMS *m*/*z* 445.3675 [M+H]^+^ (calc. for C_29_H_49_O_3_, 445.3676); purity (according 4.8.) 93.9%.

##### 4.9.34. Hydroxydammarenone II (**34**)

4.1 mg, white crystals; ${\left[\alpha \right]}_{D}^{25}$ +42 (*c* 2.00, MeOH); CD: Figure S3; ^1^H and ^13^C NMR data (CDCl_3_, 600 and 150 MHz, respectively) in Table S9; HRESIMS *m*/*z* 443.3897 [M+H]^+^ (calc. for C_30_H_51_O_2_, 443.3884); purity (according 4.8.) 89.8%.

### 4.10. Cell Culture

#### 4.10.1. Cultivation

The human cervical cancer cell line HeLa (CCL-2^TM^) was obtained from the American Type Culture Collection (ATTC^®^, Manassas, VA, USA). The cells were cultured in Minimum Essential Medium Eagle supplemented with 10% FBS, 1% L-glutamine and 1% nonessential amino acids.

The human microvascular endothelial cell line (HMEC-1) was provided by Dr. E. Ades und F.-J. Candel (CDC, Atlanta, GA, USA), as well as Dr. T. Lawley (Emory University, Atlanta, GA, USA). These cells were cultured in EASY Endothelial Cell Growth Medium supplemented with 10% FBS, 50 ng/mL amphotericin B and 50 ng/mL gentamicin.

For both the used cell lines, mycoplasma contamination was excluded by PCR and culture from GATC Biotech AG (Konstanz, Germany) and they were cultured in an atmosphere of 5% CO_2_ and 90% relative humidity at 37 °C.

#### 4.10.2. MTT Assay

The MTT assay was conducted with both cell lines and differed (besides the different respective medium) only in the seeded cell density: HeLa experiments, 8 × 10^4^ cells/well, HMEC-1 experiments 9 × 10^4^ cells/well. They were seeded (in 100 μL/well) in 96-well plates and incubated at 37 °C in an atmosphere of 5% CO_2_ and 90% relative humidity. After 24 h, the medium was replaced by a sample solution in medium (25–100 µM, max. 0.2% DMSO, *v*/*v*) and kept for another 24 h at the same conditions. Subsequently, the supernatant was removed and 100 μL of an MTT solution in medium (0.4 mg/mL) was added and incubated for three hours. Thereafter, cells were treated with 10% sodium dodecyl sulfate (SDS) in water and stored at room temperature in the dark until the formazan crystals were dissolved and the absorbance at 560 nm could be determined using a Tecan microplate reader (Tecan Trading AG, Maennedorf, Switzerland). The cell viability was calculated as proportion compared to the average absorbance of the negative control group (only medium). To preclude the possibility of solvent effects, some cells were also treated with the highest used DMSO concentration. All tests were performed three times independently in hexaplicates.

#### 4.10.3. ICAM-1 Assay

This assay was performed as previously described [66]. Confluently grown HMEC-1 cells from a culture flask (150 cm^2^) were split (1:3), suspended in 13 mL of medium, and seeded in a 24-well plate (500 µL/well). After cultivation for 48 h at 37 °C in an atmosphere of 5% CO_2_ and 90% relative humidity and the formation of a monolayer, the supernatant was replaced by substance dilutions in medium (6–70 µM) containing a maximum of 0.15% DMSO (*v*/*v*) and incubated for 30 min before stimulation with TNFα (10 ng/mL). In each performance, an unstimulated negative control (0.15% DMSO, *v*/*v*), an untreated control (medium), and a positive control (parthenolide, 5 µM) were included. 24 h later, the cells were washed with phosphate-buffered saline (PBS), detached by trypsin/EDTA, fixed by formalin 10% for 15 min, and treated with a murine fluorescein-isothiocyanate (FITC)-marked IgG1 antibody against ICAM-1 (Bio-Rad, Kidlington, UK) for 30 min. The cell suspension in PBS was analysed by a FACSCanto II (BD, Lakes, NJ, USA) (Flow 60 µL/min, FSC: 1 V, SSC: 320 V; FITC: 320 V). The ICAM-1 expression was calculated as a proportion of the mean obtained for the untreated control. The test was performed three times independently in duplicate.

### 4.11. Statistics

Significance levels were calculated in a one-way ANOVA followed by Tukey-HSD in SPSS 26 (IBM, Armonk, NY, USA), whereas nonlinear regression curves were determined by GraphPad Prism 5.0.0 (GraphPad Software, San Diego, CA, USA).

## Figures and Tables

**Table 1:** ^1^H and ^13^C NMR data for compounds **1**–**3** (600 and 150 MHz, respectively) in CDCl_3_ (*δ* in ppm, *J* in Hz, s: singlet, d: doublet, br: broad, and m: multiplet).

No.	1		2		3	
	*δ_H_*	*δ_C_*	*δ_H_*	*δ_C_*	*δ_H_*	*δ_C_*
1	7.02 (1H, d, 7.1)	128.5	7.04 (1H, d, 7.7)	128.6	5.83 (1H, dd, 11.0, 17.6)	149.6
2	7.09 (1H, dd, 6.9, 8.0)	127.3	7.10 (1H, dd, 7.1, 8.2)	127.2	4.90 (1H, brd, 18.7) 4.93 (1H, dd, 1.4, 12.4)	110.7
3	7.02 (1H, d, 7.1)	128.5	7.04 (1H, d, 7.7)	128.6	4.69 (1H, brs) 4.90 (1H, brs)	112.8
4		136.8		136.8		146.8
5		132.7		134.8	2.57 (1H, dd, 3.0, 13.5)	46.2
6	3.64 (1H, d, 17.6) 3.72 (1H, d, 18.2)	28.3	4.09 (2H, s)	25.6	1.54 (1H, ddd, 1.8, 2.9, 14.2) 2.35 (1H, dd, 13.8, 13.8)	33.3
7		160.9		135.9		73.9
8	4.72 (1H, q, 7.0)	79.1		149.2	4.97 ^1^ (1H, m)	72.3
9	1.29 (3H, d, 6.6)	18.7	8.84 (1H, s)	178.5	1.56 (1H, dd, 3.0, 15.1) 2.06 (1H, dd, 3.3, 14.9)	38.3
10		136.8		136.8		39.0
11		123.4		123.5		148.5
12		174.4	7.37 (1H, s)	144.8	4.95 (1H, brs) 5.05 (1H, brs)	112.4
13	1.47 (3H, brs)	8.3	1.83 (3H, s)	8.1	1.79 (3H, s)	18.5
14	2.24 (3H, s)	20.3	2.26 (3H, s)	20.2	1.06 (3H, s)	18.6
15	2.24 (3H, s)	20.3	2.26 (3H, s)	20.2	1.76 (3H, s)	24.9
1′						169.7
2′					2.00 (3H, s)	21.2

^1^ overlapped signal.

**Table 2:** ^1^H and ^13^C NMR data for compounds **4**, **5** (600 and 150 MHz, respectively) and **6** (400 and 100 MHz, respectively) in CDCl_3_ (*δ* in ppm, *J* in Hz, s: singlet, d: doublet, br: broad, and m: multiplet).

No.	4		5		6	
	*δ_H_*	*δ_C_*	*δ_H_*	*δ_C_*	*δ_H_*	*δ_C_*
1		130.4	4.99 (1H, d, 10.8)	132.1	4.89 (1H, d, 10.0)	133.3
2	4.33 (1H, dd, 2.8, 2.8)	73.9	4.13 (1H, ddd, 5.0, 10.1, 10.5)	78.2	4.16 (1H, ddd, 5.0, 10.1, 10.1)	77.2
3	1.28 ^1^ (1H, m) 2.28 ^1^ (1H, m)	34.0	1.96 (1H, dd, 11.3, 11.3) 2.55 (1H, dd, 5.0, 11.3)	45.9	1.94 (1H, dd, 11.0, 11.0) 2.54 (1H, dd, 5.0, 10.1)	45.3
4	2.08 (1H, m)	22.7		134.8		133.3
5	2.24 ^1^ (1H, m) 2.72 (1H, ddd, 1.7, 4.7, 16.5)	36.2	4.96 (1H, dd, 4.4, 10.5)	124.9	4.56 (1H, brd, 11.2)	126.1
6		134.8	3.14 (1H, dd, 3.3, 18.2) 3.34 (1H, dd, 10.5, 17.9)	27.0	2.88 (1H, dd, 11.2, 14.9) 3.42 (1H, brd, 14.7)	27.7
7		123.3		162.0		162.2
8		152.6	5.12 (1H, dd, 6.6, 6.6)	83.3	4.93 (1H, dd, 4.3, 11.7)	82.6
9	6.84 (1H, s)	110.7	2.23 (1H, dd, 6.6, 14.5) 3.06 (1H, dd, 6.6, 14.5)	42.9	2.12 (1H, dd, 11.7, 12.4) 3.09 (1H, dd, 4.3, 12.8)	46.8
10		140.1		135.4		134.5
11	3.64 ^1^ (1H, m)	38.2		125.9		126.3
12		178.6		173.5		173.6
13	1.55 (3H, d, 7.7)	15.4	1.88 (3H, brs)	9.3	1.87 (3H, brs)	8.9
14	2.37 (3H, s)	19.3	1.44 (3H, s)	19.6	1.57 (3H, s)	17.4
15	1.13 (3H, d, 6.6)	22.1	1.61 (3H, s)	18.0	1.67 (3H, brs)	18.0
1′	3.44 (3H, s)	56.2	3.35 (3H, s)	57.4	3.34 (3H, s)	57.4

^1^ overlapped signal.

**Table 3:** ^1^H and ^13^C NMR data for compounds **7** and **8** (600 and 150 MHz, respectively) in CDCl_3_ (*δ* in ppm, *J* in Hz, s: singlet, d: doublet, br: broad, m: multiplet).

No.	7		8 (Part I)		8 (Part II)		
	*δ_H_*	*δ_C_*	*δ_H_*	*δ_C_*	No.	*δ_H_*	*δ_C_*
1	5.72 (1H, d, 8.8)	135.0	2.71 ^1^ (1H, d, 8.8)	57.5	1’	5.49 (1H, d, 16.0)	143.4
2	5.86 (1H, dd, 5.0, 9.4)	123.7	3.48 ^1^ (1H, m)	83.5	2’	5.58 (1H, dd, 9.1, 15.7)	133.6
3	5.79 (1H, m)	120.9	1.14 ^1^ (1H, m) 2.01 ^1^ (1H, m)	39.8	3’	3.03 (1H, dd, 9.1, 9.1)	88.9
4		135.8	2.70 ^1^ (1H, m)	33.1	4’	2.57 ^1^ (1H, m)	37.4
5	2.87 (1H, brd, 14.3)	44.3	2.29 (1H, dd, 9.6, 9.6)	59.2	5’	2.39 ^1^ (1H, m) 2.46 (1H, dd, 11.8, 14.6)	48.5
6	2.54 (1H, ddd, 2.0, 2.0, 16.2) 2.96 (1H, dd, 4.4, 17.1)	21.3		196.5	6’		202.4
7		148.5		122.5	7’		117.6
8		147.9		159.0	8’		153.5
9	5.77 (1H, s)	117.7	3.50 (1H, s)	54.7	9’	2.59 (1H, d, 14.3) 2.96 (1H, d, 14.3)	35.1
10		37.8		36.1	10’	-	42.0
11		121.1		121.1	11’	-	128.6
12		171.2	7.16 (1H, s)	139.2	12’	6.92 (1H, s)	138.3
13	1.93 (3H, brs)	8.6	2.21 (3H, s)	10.2	13’	2.01 (3H, s)	9.4
14	1.02 (3H, s)	17.9	1.18 (3H, s)	25.6	14’	1.84 (1H, d, 11.6) 2.39 ^1^ (1H, m)	42.9
15	1.90 (3H, brs)	20.0	1.12 (3H, d, 6.6)	19.4	15’	1.15 ^1^ (3H, d, 6.1)	18.5
16	3.44 (3H, s)	56.2	3.33 (3H, s)	56.3	16’	3.29 (3H, s)	56.6

^1^ overlapped signal.

**Table 4:** ^1^H and ^13^C NMR data for compounds **23** and **24** (600 and 150 MHz, respectively) in CDCl_3_ (*δ* in ppm, *J* in Hz, s: singlet, d: doublet, br: broad, and m: multiplet).

No.	23		24	
	*δ_H_*	*δ_C_*	*δ_H_*	*δ_C_*
1	3.94 (1H, brs)	75.1	3.22 (1H, d, 3.9)	59.6
2	3.75 (1H, dd, 2.9, 9.8)	72.3	3.12 (1H, d, 3.9)	57.5
3	4.77 (1H, dd, 9.9, 9.9)	80.0	3.47 ^1^ (1H, m)	72.8
4	1.63 ^1^ (1H, m)	34.8	1.29 ^1^ (1H, m)	36.1
5	1.62 ^1^ (1H, m)	39.3	1.21 ^1^ (1H, m)	35.6
6	1.32 ^1^ (1H, m) 1.80 ^1^ (1H, m)	20.1	1.33 ^1^ (1H, m) 1.74 ^1^ (1H, m)	19.2
7	2.04 ^1^ (1H, m) 2.10 ^1^ (1H, m)	25.8	2.03 ^1^ (2H, m)	24.5
8		138.6		136.5
9		130.0		130.3
10		41.8		37.6
11	2.12 ^1^ (1H, m) 2.22 ^1^ (1H, m)	21.5	2.23 (1H, m) 2.29 (1H, m)	22.3
12	1.75 ^1^ (1H, m) 1.82 ^1^ (1H, m)	30.8	1.76 ^1^ (1H, m) 1.82 ^1^(1H, m)	30.7
13		44.6		44.5
14		50.1		49.9
15	1.21 ^1^ (1H, m) 1.60 ^1^ (1H, m)	30.8	1.18 ^1^ (1H, m) 1.57 ^1^ (1H, m)	30.6
16	1.33 ^1^ (1H, m) 1.93 ^1^ (1H, m)	28.1	1.33 ^1^ (1H, m) 1.93 (1H, m)	28.1
17	1.52 ^1^ (1H, m)	50.3	1.52 ^1^ (1H, m)	50.2
18	0.72 (3H, s)	15.7	0.74 ^1^ (3H, s)	15.7
19	1.00 ^1^ (3H, s)	18.2	1.06 (3H, s)	17.9
20	1.40 ^1^ (1H, m)	36.3	1.40 ^1^ (1H, m)	36.3
21	0.93 ^1^ (3H, m)	18.7	0.93 (3H, d, 6.6)	18.7
22	1.05 ^1^ (1H, m) 1.45 ^1^ (1H, m)	36.3	1.05 ^1^ (1H, m) 1.45 ^1^ (1H, m)	36.3
23	1.86 ^1^ (1H, m) 2.04 ^1^ (1H, m)	25.0	1.87 ^1^ (1H, m) 2.04 ^1^ (1H, m)	24.9
24	5.10 (1H, dd, 7.2, 7.2)	125.0	5.10 (1H, dd, 7.2, 7.2)	125.2
25		130.9		131.0
26	1.61 (3H, s)	17.6	1.61 (3H, s)	17.6
27	1.69 (3H, s)	25.6	1.69 (3H, s)	25.7
28	0.93 (3H, s)	24.9	0.91 (3H, s)	24.3
29	0.91 (3H, d, 6.1)	15.0	0.97 (3H, d, 6.6)	16.3
30				
1′		175.2		
2′	2.27 ^1^ (2H, m)	43.7		
3′	2.16 ^1^ (1H, m)	25.7		
4′	0.99 (3H, d, 6.8)	22.4		
5′	0.99 (3H, d, 6.8)	22.5		

^1^ overlapped signal.

**Table 5:** The gradients consisting of water and ACN used to separate the respective fractions and the retention times of the isolates. A biphenyl column as specified in the text was used for the separation of all fractions except M1.2C6 and M1.2C7, for which the Nucleodur C18 was employed.

Fraction	Gradient		Retention Time [min], Isolate	Fraction	Gradient		Retention Time [min], Isolate
	Time [min]	ACN [%]			Time [min]	ACN [%]	
F5C2	0 6 10 11 16	65 79 88 100 100	8.8, **26**	F7C7F2	0 19 20 25	30 50 100 100	16.1, **6** 15.4, **12**
F5C5	0 16 17 22	45 53 100 100	16.0, **9** 11.9, **15**	F7C7F3	0 19 20 25	25 45 100 100	18.1, **5**
F6C3	0 15 20	70 100 100	11.6, **23**	F9C2	0 15 16 21	60 80 100 100	14.7, **31**
F6C4	0 20 21 26	60 80 100 100	17.3, **24**	F9C3	0 15 16 21	60 80 100 100	13.6, **28** 11.8, **29**
F6C5	0 13 14 19	60 68 100 100	11.1, **34**	F9C4	0 12 13 16	60 76 100 100	9.7, **33**
F6C7F3	0 19 23 30 35	30 50 80 100 100	21.6, **16** 11.6, **17** 26.8, **25**	M1.2C3	0 15 25 26 31	40 50 60 95 95	20.7, **13** and **22**
F7C2	0 26 36 41	60 70 100 100	30.2, **30** 33.8, **32**	M1.2C6	0 20 21 26	50 60 90 90	14.0, **1** 17.4, **19** and **21** 18.8, **20** 20.6, **4** 21.2, **3** 27.2, impure **8**
F7C3	0 10 11 17	60 80 100 100	9.9, **27**	27.2, from M1.2C6	0 16 17 26	57 68 90 90	15.2, **8**
F7C7F1	0 13 14 19	40 54 100 100	11.3, **10** 12.4, **11**	M1.2C7	0 20 21 31	55 65 100 100	11.8, **14** 13.1, **2** 16.2, **7** 16.9, **18**

## Data Availability

The data presented in this study are available on request from J.H., K.K., and A.U.

## Supplementary Materials

The following supporting information can be downloaded at: https://www.mdpi.com/article/10.3390/molecules28041637/s1, Figure S1. (a) Molecular structures of three conformers of commiphorine A underlying the ECD simulations published by previous authors [21]. All structures represent a compound with 1,5-*cis* ring fusion in the guaiane part (H-1 and H-5 are *cis*-oriented; the latter is marked by a dashed circle). This arrangement does not correspond to the structure of commiphorine A with a 1,5-*trans*-guaiane moiety postulated in the same work [21]. (b) Experimental ECD spectrum of commiphorine A (red curve) and simulated ECD spectrum (blue curve) obtained with the structures shown in (a) according to [21]. (c) ECD spectrum simulated for commiphorine A with the erroneous 1,5-*cis* structure (blue curve) and with the correct 1,5-*trans* structure (red curve). Table S1. ^1^H and ^13^C NMR data (400 and 100 MHz, respectively) for compounds **9**–**11** (CDCl_3_, δ in ppm, J in Hz, s: singlet, d: doublet, br: broad, and m: multiplet). Table S2. ^1^H and ^13^C NMR data (600 and 150 MHz, respectively) for compounds **12**, **13** and (400 and 100 MHz, respectively) for **14** (CDCl_3_, δ in ppm, J in Hz, s: singlet, d: doublet, br: broad, and m: multiplet). Table S3. ^1^H and ^13^C NMR data (400 and 100 MHz, respectively) for compound **15** and (600 and 150 MHz, respectively) for **16** and **17** (CDCl_3_, δ in ppm, J in Hz, s: singlet, d: doublet, br: broad, and m: multiplet). Table S4. ^1^H and ^13^C NMR data (600 and 150 MHz, respectively) for compounds **18**–**20** (CDCl_3_, δ in ppm, J in Hz, s: singlet, d: doublet, br: broad, and m: multiplet). Table S5. ^1^H and ^13^C NMR data (600 and 150 MHz, respectively) for compounds **21** and **22** (CDCl_3_, δ in ppm, J in Hz, s: singlet, d: doublet, br: broad, and m: multiplet). Table S6. ^1^H and ^13^C NMR data (600 and 150 MHz, respectively) for compound **25** and (400 and 100 MHz, respectively) for **26** and **27** (CDCl_3_, δ in ppm, J in Hz, s: singlet, d: doublet, br: broad, and m: multiplet). Table S7. ^1^H and ^13^C NMR data (600 and 150 MHz, respectively) for compounds **28**, **29** and (400 and 100 MHz, respectively) for **30** (CDCl_3_, δ in ppm, J in Hz, s: singlet, d: doublet, br: broad, and m: multiplet). Table S8. ^1^H and ^13^C NMR data (400 and 100 MHz, respectively) for compounds **31** and **32** and (600 and 150 MHz, respectively) for **33** (CDCl_3_, δ in ppm, J in Hz, s: singlet, d: doublet, br: broad, and m: multiplet). Table S9. ^1^H and ^13^C NMR data (600 and 150 MHz, respectively) for compound **34** (CDCl_3_, δ in ppm, J in Hz, s: singlet, d: doublet, br: broad, and m: multiplet). Figure S2. CD spectra of compounds **1**, **3**–**12** and **14**–**17** in methanol. [*θ*] in [(°cm^2^) · dmol^−1^]. Figure S3. CD spectra of compounds **18**, **20** and **22**–**34** in methanol. [*θ*] in [(°cm^2^) · dmol^−1^]. Figure S4. ^1^H (600 MHz, A) and ^13^C NMR spectra (150 MHz, B) of **1** (in CDCl_3_). Figure S5. ^1^H (600 MHz, A) and ^13^C NMR spectra (150 MHz, B) of **2** (in CDCl_3_). Figure S6. ^1^H (600 MHz, A) and ^13^C NMR spectra (150 MHz, B) of **3** (in CDCl_3_). Figure S7. ^1^H (600 MHz, A) and ^13^C NMR spectra (150 MHz, B) of **4** (in CDCl_3_). Figure S8. ^1^H (600 MHz, A) and ^13^C NMR spectra (150 MHz, B) of **5** (in CDCl_3_). Figure S9. ^1^H (400 MHz, A) and ^13^C NMR spectra (100 MHz, B) of **6** (in CDCl_3_). Figure S10. ^1^H (600 MHz, A) and ^13^C NMR spectra (150 MHz, B) of **7** (in CDCl_3_). Figure S11. ^1^H (600 MHz, A) and ^13^C NMR spectra (150 MHz, B) of **8** (in CDCl_3_). Figure S12. ^1^H (600 MHz, A) and ^13^C NMR spectra (150 MHz, B) of **23** (in CDCl_3_). Figure S13. ^1^H (600 MHz, A) and ^13^C NMR spectra (150 MHz, B) of **24** (in CDCl_3_). Figure S14. Influence of compounds **4**–**6**, **9**–**11**, **17**, **18**, **20** and **27** on the viability of HMEC-1 cells in the MTT assay (a) and on their TNFα-induced ICAM-1 expression (b). The test was performed with medium containing DMSO (0.15%, *v*/*v*) without stimulation (u.c.), with TNFα (10 ng/mL, n.c.), and with both TNFα and parthenolide (5 µM, Parth.) as positive control. Substances were applied in concentrations between 13 and 70 µM. Data are presented as mean ± SEM (n = 3).

## Sample Availability

Samples of the compounds **1**–**34** are available from the authors.

## References

[1] Tucker A.O. (1986). Frankincense and myrrh. Econ. Bot..

[2] Shen T., Li G.-H., Wang X.-N., Lou H.-X. (2012). The genus *Commiphora*: A review of its traditional uses, phytochemistry and pharmacology. J. Ethnopharmacol..

[3] Abegaz B., Dagne E., Bates C., Waterman P.G. (1989). Monoterpene-rich resins from two ethiopian species of *Commiphora*. Flavour Fragr. J..

[4] Asres K., Tei A., Moges G., Sporer F., Wink M. (1998). Terpenoid composition of the wound-induced bark exudate of *Commiphora tenuis* from Ethiopia. Planta Med..

[5] Hanuš L.O., Řezanka T., Dembitsky V.M., Moussaieff A. (2005). Myrrh-*Commiphora* chemistry. Biomed. Papers.

[6] Dekebo A., Dagne E., Sterner O. (2002). Furanosesquiterpenes from *Commiphora sphaerocarpa* and related adulterants of true myrrh. Fitoterapia.

[7] Morteza-Semnani K., Saeedi M. (2003). Constituents of the essential oil of *Commiphora myrrha* (Nees) Engl. var. molmol. J. Essent. Oil Res..

[8] Madia V.N., de Angelis M., de Vita D., Messore A., de Leo A., Ialongo D., Tudino V., Saccoliti F., de Chiara G., Garzoli S. (2021). Investigation of *Commiphora myrrha* (Nees) Engl. oil and its main components for antiviral activity. Pharmaceuticals.

[9] Marongiu B., Piras A., Porcedda S., Scorciapino A. (2005). Chemical composition of the essential oil and supercritical CO_2_ extract of *Commiphora myrrha* (Nees) Engl. and of *Acorus calamus* L.. J. Agric. Food Chem..

[10] Nikolic M., Smiljkovic M., Markovic T., Ciric A., Glamoclija J., Markovic D., Sokovic M. (2016). Sensitivity of clinical isolates of *Candida* to essential oils from Burseraceae family. EXCLI J..

[11] Hanuš L.O., Rosenthal D., Řezanka T., Dembitsky V.M., Moussaief A. (2008). Fast and easy GC/MS identification of myrrh resins. Pharm. Chem. J..

[12] Su S.L., Duan J.A., Tang Y.P., Zhang X., Yu L., Jiang F.R., Zhou W., Luo D., Ding A.W. (2009). Isolation and biological activities of neomyrrhaol and other terpenes from the resin of *Commiphora myrrha*. Planta Med..

[13] Xu J., Guo Y., Zhao P., Xie C., Jin D., Hou W., Zhang T. (2011). Neuroprotective cadinane sesquiterpenes from the resinous exudates of *Commiphora myrrha*. Fitoterapia.

[14] Xu J., Guo Y., Li Y., Zhao P., Liu C., Ma Y., Gao J., Hou W., Zhang T. (2011). Sesquiterpenoids from the resinous exudates of *Commiphora myrrha* and their neuroprotective effects. Planta Med..

[15] Greve H.L., Kaiser M., Schmidt T.J. (2020). Investigation of antiplasmodial effects of terpenoid compounds isolated from myrrh. Planta Med..

[16] Shen T., Wan W.-Z., Wang X.-N., Yuan H.-Q., Ji M., Lou H.-X. (2009). A triterpenoid and sesquiterpenoids from the resinous exudates of *Commiphora myrrha*. Helv. Chim. Acta.

[17] Ayyad S.-E.N., Hoye T.R., Alarif W.M., Al Ahmadi S.M., Basaif S.A., Ghandourah M.A., Badria F.A. (2015). Differential cytotoxic activity of the petroleum ether extract and its furanosesquiterpenoid constituents from *Commiphora molmol* resin. Z. Naturforsch..

[18] Kuck K., Jürgenliemk G., Lipowicz B., Heilmann J. (2020). Sesquiterpenes from myrrh and their ICAM-1 inhibitory activity in vitro. Molecules.

[19] Ahmed F., Ali M., Singh O. (2006). New compounds from *Commiphora myrrha* (Nees) Engl. Pharmazie.

[20] Wang C.-C., Liang N.-Y., Xia H., Wang R.-Y., Zhang Y.-F., Huo H.-X., Zhao Y.-F., Song Y.-L., Zheng J., Tu P.-F. (2022). Cytotoxic sesquiterpenoid dimers from the resin of *Commiphora myrrha* Engl. Phytochemistry.

[21] Dong L., Qin D.-P., Di Q.-Q., Liu Y., Chen W.-L., Wang S.-M., Cheng Y.-X. (2019). Commiphorines A and B, unprecedented sesquiterpenoid dimers from *Resina Commiphora* with striking activities on anti-inflammation and lipogenesis inhibition. Org. Chem. Front..

[22] Liu J.-W., Liu Y., Yan Y.-M., Yang J., Lu X.-F., Cheng Y.-X. (2018). Commiphoratones A and B, two sesquiterpene dimers from *Resina Commiphora*. Org. Lett..

[23] Hu B.-Y., Qin D.-P., Wang S.-X., Qi J.-J., Cheng Y.-X. (2018). Novel terpenoids with potent cytotoxic activities from *Resina Commiphora*. Molecules.

[24] Zhu C.-Z., Hu B.-Y., Liu J.-W., Cai Y., Chen X.-C., Qin D.-P., Cheng Y.-X., Zhang Z.-D. (2019). Anti-*Mycobacterium tuberculosis* terpenoids from *Resina Commiphora*. Molecules.

[25] Liu J.-W., Zhang M.-Y., Yan Y.-M., Wei X.-Y., Dong L., Zhu Y.-X., Cheng Y.-X. (2018). Characterization of sesquiterpene dimers from *Resina Commiphora* that promote adipose-derived stem cell proliferation and differentiation. J. Org. Chem..

[26] Dong L., Jiang J.-B., Yan Y.-M., Wang S.-M., Cheng Y.-X. (2021). Commiphoroids G1—G3, H and I, Five Terpenoid Dimers as Extracellular Matrix Inhibitors from *Resina Commiphora*. Chin. J. Chem..

[27] Ma Y.-H., Dou X.-X., Tian X.-H. (2020). Natural disesquiterpenoids: An overview of their chemical structures, pharmacological activities, and biosynthetic pathways. Phytochem. Rev..

[28] Zhan Z.-J., Ying Y.-M., Ma L.-F., Shan W.-G. (2011). Natural disesquiterpenoids. Nat. Prod. Rep..

[29] Cao B., Wei X.-C., Xu X.-R., Zhang H.-Z., Luo C.-H., Feng B., Xu R.-C., Zhao S.-Y., Du X.-J., Han L. (2019). Seeing the unseen of the combination of two natural resins, frankincense and myrrh: Changes in chemical constituents and pharmacological activities. Molecules.

[30] Langhorst J., Varnhagen I., Schneider S.B., Albrecht U., Rueffer A., Stange R., Michalsen A., Dobos G.J. (2013). Randomised clinical trial: A herbal preparation of myrrh, chamomile and coffee charcoal compared with mesalazine in maintaining remission in ulcerative colitis—A double-blind, double-dummy study. Aliment. Pharmacol. Ther..

[31] Boffa L., Binello A., Boscaro V., Gallicchio M., Amisano G., Fornasero S., Cravotto G. (2016). *Commiphora myrrha* (Nees) Engl. extracts: Evaluation of antioxidant and antiproliferative activity and their ability to reduce microbial growth on fresh-cut salad. Int. J. Food Sci. Technol..

[32] Shoemaker M., Hamilton B., Dairkee S.H., Cohen I., Campbell M.J. (2005). In vitro anticancer activity of twelve chinese medicinal herbs. Phytother. Res..

[33] Tipton D.A., Lyle B., Babich H., Dabbous M.K. (2003). In vitro cytotoxic and anti-inflammatory effects of myrrh oil on human gingival fibroblasts and epithelial cells. Toxicol. In Vitro.

[34] Al-Harbi M.M., Qureshi S., Raza M., Ahmed M.M., Giangreco A.B., Shah A.H. (1994). Anticarcinogenic effect of *Commiphora molmol* on solid tumors induced by Ehrlich carcinoma cells in mice. Chemotherapy.

[35] Liaw C.-C., Chen Y.-C., Huang G.-J., Tsai Y.-C., Chien S.-C., Wu J.-H., Wang S.-Y., Chao L.K., Sung P.-J., Huang H.-C. (2013). Anti-inflammatory lanostanoids and lactone derivatives from *Antrodia camphorata*. J. Nat. Prod..

[36] Silva M.d.L.e., David J.P., Silva L.C.R.C., Santos R.A.F., David J.M., Lima L.S., Reis P.S., Fontana R. (2012). Bioactive oleanane, lupane and ursane triterpene acid derivatives. Molecules.

[37] Baltina L.A., Flekhter O.B., Nigmatullina L.R., Boreko E.I., Pavlova N.I., Nikolaeva S.N., Savinova O.V., Tolstikov G.A. (2003). Lupane triterpenes and derivatives with antiviral activity. Bioorg. Med. Chem. Lett..

[38] Ahmed Y., Sohrab M.H., Al-Reza S.M., Tareq F.S., Hasan C.M., Sattar M.A. (2010). Antimicrobial and cytotoxic constituents from leaves of *Sapium baccatum*. Food Chem. Toxicol..

[39] Mokoka T.A., McGaw L.J., Mdee L.K., Bagla V.P., Iwalewa E.O., Eloff J.N. (2013). Antimicrobial activity and cytotoxicity of triterpenes isolated from leaves of *Maytenus undata* (Celastraceae). BMC Complement. Altern. Med..

[40] Chudzik M., Korzonek-Szlacheta I., Król W. (2015). Triterpenes as potentially cytotoxic compounds. Molecules.

[41] Weber L., Kuck K., Jürgenliemk G., Heilmann J., Lipowicz B., Vissiennon C. (2020). Anti-inflammatory and barrier-stabilising effects of myrrh, coffee charcoal and chamomile flower extract in a co-culture cell model of the intestinal mucosa. Biomolecules.

[42] Provan G.J., Gray A.I., Waterman P.G. (1987). Sesquiterpenes from the myrrh-type resins of some kenyan *Commiphora* species. Flavour Fragr. J..

[43] Brieskorn C.H., Noble P. (1980). Drei neue Furanogermacrene aus Myrrhe. Tetrahedron Lett..

[44] Santoro E., Messina F., Marcotullio M.C., Superchi S. (2014). Absolute configuration of bioactive furanogermacrenones from *Commiphora erythraea* (Ehrenb) Engl. by computational analysis of their chiroptical properties. Tetrahedron.

[45] Brieskorn C.H., Noble P. (1983). Furanosesquiterpenes from the essential oil of myrrh. Phytochemistry.

[46] Lange G.L., Lee M. (1986). ^13^C NMR determination of the configuration of methyl-substituted double bonds in medium- and large-ring terpenoids. Magn. Reson. Chem..

[47] Hikino H., Konno C., Agatsuma K., Takemoto T., Horibe I., Tori K., Ueyama M., Takeda K. (1975). Sesquiterpenoids. Part XLVII. Structure, configuration, conformation, and thermal rearrangement of furanodienone, isofuranodienone, curzerenone, epicurzerenone, and pyrocurzerenone, sesquiterpenoids of *Curcuma zedoaria*. J. Chem. Soc. Perkin Trans..

[48] Stahl E., Datta S.N. (1972). Neue sesquiterpenoide Inhaltsstoffe der Gundelrebe (*Glechoma hederacea* L.). Justus Liebigs Ann. Chem..

[49] Shen T., Wan W.-Z., Wang X.-N., Sun L.-M., Yuan H.-Q., Wang X.-L., Ji M., Lou H.-X. (2008). Sesquiterpenoids from the resinous exudates of *Commiphora opobalsamum* (Burseraceae). Helv. Chim. Acta.

[50] Zhao N., Yang G.-C., Li D.-H., Li X.-Y., Li Z.-L., Bai J., Liu X.-Q., Hua H.-M. (2015). Two new sesquiterpenes from myrrh. Helv. Chim. Acta.

[51] Zhu N., Kikuzaki H., Sheng S., Sang S., Rafi M.M., Wang M., Nakatani N., DiPaola R.S., Rosen R.T., Ho C.-T. (2001). Furanosesquiterpenoids of *Commiphora myrrha*. J. Nat. Prod..

[52] Ulubelen A., Gören N., Jakupovic J. (1986). Germacrane derivatives from the fruits of *Smyrnium creticum*. Phytochemistry.

[53] Gao J.-F., Xie J.-H., Iitaka Y., Inayama S. (1989). The stereostructure of wenjine and related (1S,10S),(4S,5S)-germacrone-1(10),4-diepoxide isolated from *Curcuma wenyujin*. Chem. Pharm. Bull..

[54] Tada H., Minato H., Takeda K. (1971). Components of the root of *Lindera strychnifolia* Vill. Part XVIII. Neosericenyl acetate and dehydrolindestrenolide. J. Chem. Soc. C.

[55] Duh C.-Y., Chen K.-J., El-Gamal A.A.H., Dai C.-F. (2001). Sesquiterpenes from the formosan stolonifer *Tubipora musica*. J. Nat. Prod..

[56] Endo K., Taguchi T., Taguchi F., Hikino H., Yamahara J., Fujimura H. (1979). Antiinflammatory principles of *Atractylodes* rhizomes. Chem. Pharm. Bull..

[57] Friedrich D., Bohlmann F. (1988). Total synthesis of various elemanolides. Tetrahedron.

[58] Provan G.J., Waterman P.G. (1988). Major triterpenes from the resins of *Commiphora incisa* and *C. kua* and their potential chemotaxonomic significance. Phytochemistry.

[59] Provan G.J., Waterman P.G. (1986). The mansumbinanes: Octanordammaranes from the resin of *Commiphora incisa*. Phytochemistry.

[60] Rahman M.M., Garvey M., Piddock L.J.V., Gibbons S. (2008). Antibacterial terpenes from the oleo-resin of *Commiphora molmol* (Engl.). Phytother. Res..

[61] Shen T., Yuan H.-Q., Wan W.-Z., Wang X.-L., Wang X.-N., Ji M., Lou H.-X. (2008). Cycloartane-type triterpenoids from the resinous exudates of *Commiphora opobalsamum*. J. Nat. Prod..

[62] Shen T., Wan W., Yuan H., Kong F., Guo H., Fan P., Lou H. (2007). Secondary metabolites from *Commiphora opobalsamum* and their antiproliferative effect on human prostate cancer cells. Phytochemistry.

[63] Gao W., Su X., Dong X., Chen Y., Zhou C., Xin P., Yu C., Wei T. (2015). Cycloartan-24-ene-1α,2α,3β-triol, a cycloartane-type triterpenoid from the resinous exudates of *Commiphora myrrha*, induces apoptosis in human prostatic cancer PC-3 cells. Oncol. Rep..

[64] Mills J.S., Werner A.E.A. (1955). The chemistry of dammar resin. J. Chem. Soc..

[65] Asakawa J., Kasai R., Yamasaki K., Tanaka O. (1977). ^13^C NMR study of ginseng sapogenins and their related dammarane type triterpenes. Tetrahedron.

[66] Freischmidt A., Jürgenliemk G., Kraus B., Okpanyi S.N., Müller J., Kelber O., Weiser D., Heilmann J. (2012). Contribution of flavonoids and catechol to the reduction of ICAM-1 expression in endothelial cells by a standardised willow bark extract. Phytomedicine.

[67] Mosmann T. (1983). Rapid colorimetric assay for cellular growth and survival: Application to proliferation and cytotoxicity assays. J. Immunol. Methods.

[68] Monographie Myrrhe, Myrrha (2020). Europäisches Arzneibuch 10. Ausgabe, Grundwerk 2020: Amtliche Deutsche Ausgabe (Ph. Eur. 10.0).

[69] Alqahtani A.S., Nasr F.A., Noman O.M., Farooq M., Alhawassi T., Qamar W., El-Gamal A. (2020). Cytotoxic evaluation and anti-angiogenic effects of two furano-sesquiterpenoids from *Commiphora myrrh* resin. Molecules.

[70] Marston A., Borel C., Hostettmann K. (1988). Separation of natural products by centrifugal partition chromatography. J. Chromatogr. A.

