# Supplementary material for: Terpenoids from Myrrh and Their Cytotoxic Activity against HeLa Cells
Source: Molecules. 2023 Feb 8;28(4):1637. doi: 10.3390/molecules28041637 (PMC9964945; doi:10.3390/molecules28041637)
Supplement: Supplementary file 1 [file molecules-28-01637-s001.zip › molecules-2141463-supplementary.pdf]

molecules

Supplementary material

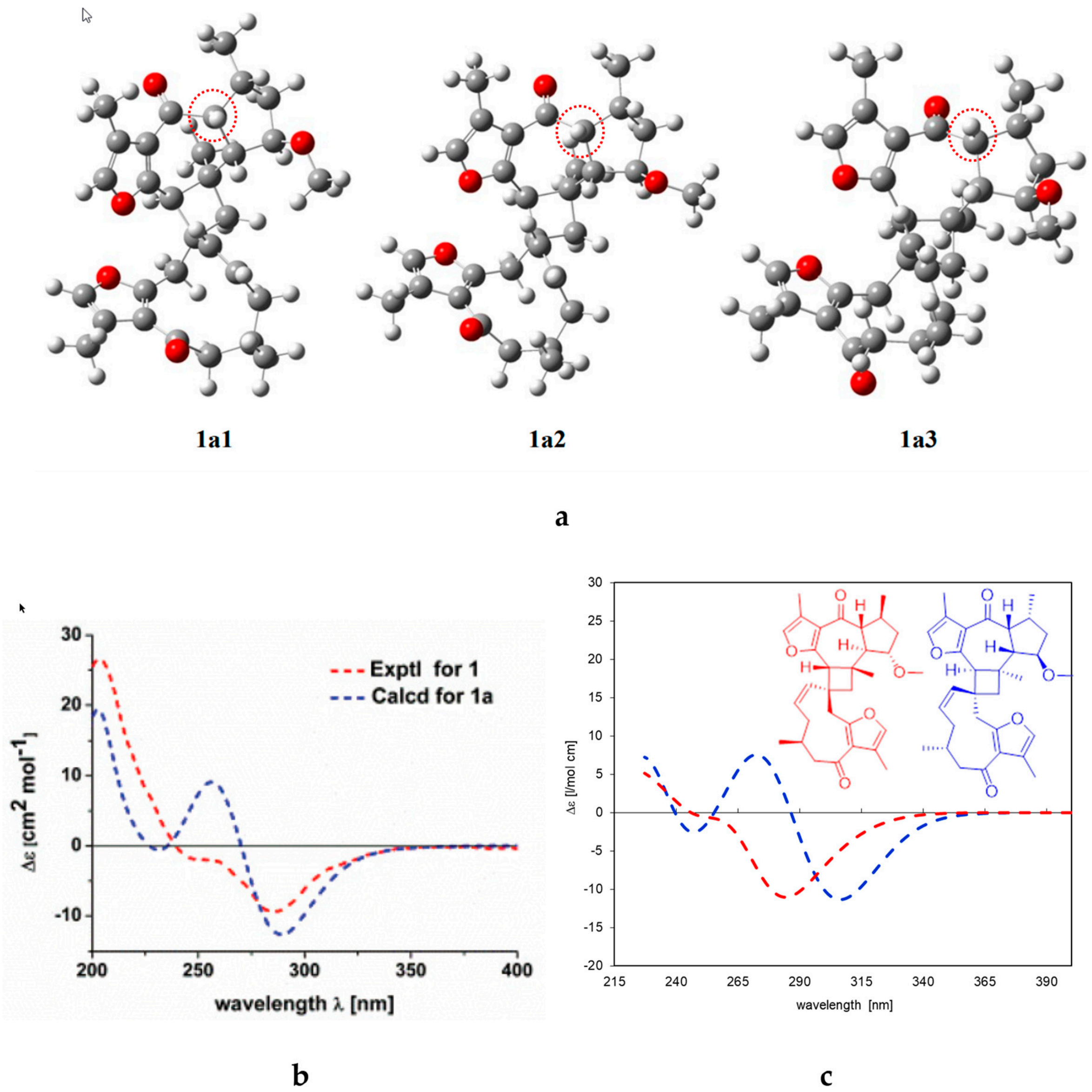

Figure S1. (**a**) Molecular structures of three conformers of commiphorine A underlying the ECD simulations published by previous authors [20]. All structures represent a compound with 1,5-*cis* ring fusion in the guaiane part (H-1 and H-5 are *cis* oriented; the latter is marked by a dashed circle). This arrangement does not correspond to the structure of commiphorine A with a 1,5-*trans*-guaiane moiety postulated in the same work [20]. (**b**) Experimental ECD spectrum of commiphorine A (red curve) and simulated ECD spectrum (blue curve) obtained with the structures shown in **a** according to [20]. (**c**) ECD spectrum simulated for commiphorine A with the erroneous 1,5-*cis* structure (blue curve) and with the correct 1,5-*trans* structure (red curve).

Table S1. ^{1}H and ^{13}C NMR data (400 and 100 MHz, respectively) for compounds **9**-**11** ($CDCl_3$, δ in ppm, J in Hz, s singlet, d doublet, br broad, m multiplet).

No.	9		10		11	
	δ_H	δ_C	δ_H	δ_C	δ_H	δ_C
1	4.85 (1H, brd, 13.7)	130.7	5.05 (1H, d, 9.7)	128.7	4.95 (1H, d, 9.8)	129.8
2	2.09[1] (1H, m)	25.6	5.53 (1H, ddd, 5.0, 10.5, 10.5)	71.4	5.54 (1H, ddd, 5.0, 10.4, 10.4)	70.8
	2.20[1] (1H, m)					
3	1.87[1] (1H, m)	38.5	2.03 (1H, dd, 12.0, 12.0)	44.9	2.02 (1H, dd, 11.1, 11.1)	44.2
	2.24[1] (1H, m)		2.57 (1H, dd, 5.0, 11.4)		2.55 (1H, dd, 5.0, 11.3)	
4		132.9		133.9		132.4
5	4.33 (1H, brd, 11.0)	123.8	4.99 (1H, dd, 4.1, 11.1)	125.8	4.60 (1H, brd, 11.1)	127.0
6	2.89 (1H, dd, 11.0, 14.6)	27.5	3.16 (1H, dd, 2.7, 17.9)	27.0	2.89 (1H, dd, 11.1, 15.0)	27.6
	3.41 (1H, brd, 14.6)		3.35 (1H, dd, 10.3, 18.2)		3.42 (1H, brd, 15.1)	
7		162.8		161.7		161.9
8	4.96 (1H, dd, 4.3, 11.5)	82.8	5.11 (1H, dd, 7.4, 7.4)	83.1	4.93[1] (1H, m)	82.6
9	2.10[1] (1H, m)	47.2	2.18 (1H, dd, 7.7, 14.1)	42.6	2.08 (1H, dd, 12.6, 12.6)	46.5
	3.10 (1H, brd, 13.0)		3.06 (1H, dd, 6.4, 14.1)		3.09 (1H, dd, 4.3, 12.6)	
10		132.4		135.3		134.4
11		126.0		126.1		126.5
12		173.8		173.4		173.5
13	1.87 (3H, s)	8.9	1.87 (3H, brs)	9.3	1.87 (3H, brs)	9.0
14	1.50 (3H, s)	16.5	1.45 (3H, s)	19.8	1.63 (3H, s)	17.4
15	1.62 (3H, s)	16.8	1.64 (3H, brs)	17.9	1.70 (3H, brs)	17.8
1′				170.6		170.6
2′			2.06 (3H, s)	21.3	2.05 (3H, s)	21.2

[1] overlapped signal

Table S2. ^{1}H and ^{13}C NMR data (600 and 150 MHz, respectively) for compounds **12**, **13** and (400 and 100 MHz, respectively) for **14** ($CDCl_3$, δ in ppm, *J* in Hz, s singlet, d doublet, br broad, m multiplet).

No.	12		13		14	
	δ_H	δ_C	δ_H	δ_C	δ_H	δ_C
1	5.20 (1H, d, 7.7)	134.0	5.28 (1H, t, 8.5)	123.9	5.96 (1H, d, 16.4)	131.1
2	4.04 (1H, ddd, 2.7, 7.4, 7.4)	75.4	2.20[1] (2H, m)	26.1	5.40 (1H, dd, 9.5, 16.4)	133.9
3	1.78 (1H, ddd, 2.9, 8.7, 14.7)<br>1.90 (1H, ddd, 4.3, 7.1, 14.3)	35.9	1.80[1] (1H, m)<br>2.27[1] (1H, m)	36.3	3.07 (1H, dd, 9.2, 9.2)	87.2
4	2.32 (1H, m)	25.3		140.8	2.61 (1H, m)	37.0
5	2.51 (1H, dd, 6.3, 17.3)<br>2.82[1] (1H, m)	47.3	5.86 (1H, s)	129.1	2.34 (1H, dd, 11.7, 15.05)<br>2.49 (1H, dd, 2.04, 15.1)	47.3
6		197.9		193.5		202.8
7		158.8		121.4		116.7
8	5.35 (1H, m)	81.1		161.5		150.3
9	2.10 (1H, dd, 4.9, 12.1)<br>2.82 (1H, dd, 12.1, 12.1)	45.8	3.06 (1H, d, 14.3)<br>3.59 (1H, d, 14.9)	32.8	3.37 (1H, d, 14.5)<br>3.75 (1H, d, 14.5)	32.7
10		133.7		134.1		141.4
11		129.8		122.9		127.5
12		172.8	7.08 (1H, brs)	138.5	6.98 (1H, brs)	136.8
13	2.01 (3H, brs)	10.3	2.19 (3H, brs)	9.5	1.98 (3H, s)	8.1
14	1.74 (3H, s)	17.4	1.66 (3H, s)	22.7	4.90 (1H, brs)<br>5.19 (1H, brs)	114.7
15	1.18 (3H, d, 7.2)	22.8	1.75 (3H, s)	19.2	1.15 (3H, d, 6.6)	17.9
1′	3.25 (3H, s)	56.0			3.23 (3H, s)	55.6

[1] overlapped signal

Table S3. ^{1}H and ^{13}C NMR data (400 and 100 MHz, respectively) for compound **15** and (600 and 150 MHz, respectively) for **16** and **17** ($CDCl_3$, δ in ppm, *J* in Hz, s singlet, d doublet, br broad, m multiplet).

No.	15		16		17	
	δ_H	δ_C	δ_H	δ_C	δ_H	δ_C
1	5.23 (1H, d, 9.4)	132.8	5.19 (1H, d, 8.8)	129.6	2.91 (1H, brd, 10.5)	61.4
2	4.13 (1H, m)	73.8	4.35 (1H, ddd, 4.0, 8.4, 12.0)	76.4	1.46 (1H, ddd, 5.8, 11.6, 14.0)<br>2.06 (1H, brdd, 5.8, 14.6)	22.8
3	1.90[1] (2H, m)	37.8	1.29[1] (1H, m)<br>1.94 (1H, ddd, 4.7, 12.9, 12.9)	39.2	1.28[1] (1H, ddd, 6.1, 13.2)<br>2.21 (1H, ddd, 2.5, 5.8, 13.7)	35.7
4	2.38 (1H, m)	30.6	2.15[1] (1H, m)	31.5		60.1
5	5.52 (1H, d, 8.4)	78.9	5.86 (1H, brs)	76.2	2.65 (1H, dd, 2.2, 11.0)	64.0
6		195.8		191.5	2.26 (1H dd, 11.0, 13.8)<br>2.86 (1H, brd, 13.8)	29.3
7		121.2		119.6		134.3
8		154.3		157.0		207.2
9	3.31 (1H, d, 16.8)<br>3.64 (1H, d, 16.8)	38.1	3.37 (1H, d, 14.9)<br>4.06 (1H, d, 14.9)	34.3	2.64 (1H, d, 11.0)<br>3.00 (1H, d, 11.0)	54.6
10		135.2		134.9		58.4
11		123.2		123.0		137.8
12	7.03 (1H, s)	138.0	7.12 (1H, s)	139.0	1.86 (3H, s)	20.8
13	1.92 (3H, s)	8.7	2.16 (3H, s)	10.4	1.79 (3H, s)	22.9
14	1.95 (3H, s)	18.8	1.86 (3H, s)	23.8	1.44 (3H, s)	17.4
15	1.08 (3H, d, 7.1)	17.3	0.91 (3H, d, 7.2)	14.1	1.14 (3H, s)	15.6
1′	3.25 (3H, s)	55.8	3.34 (3H, s)	56.8		
1′′		170.3		171.1		
2′′	2.04 (3H, s)	20.7	2.22 (3H, s)	20.7		

[1] overlapped signal

Table S4. ^{1}H and ^{13}C NMR data (600 and 150 MHz, respectively) for compounds **18**-**20** ($CDCl_3$, δ in ppm, *J* in Hz, s singlet, d doublet, t triplet, br broad, m multiplet).

No.	18		19		20	
	δ_H	δ_C	δ_H	δ_C	δ_H	δ_C
1	5.71 (1H, brd, 9.9)	135.5	5.48 (1H, d, 9.4)	130.8	1.32 (1H, ddd, 5.5, 12.9, 12.9)	40.9
					1.58[1] (1H, m)	
2	5.62 (1H, dt, 3.4, 9.6)	125.0	5.93 (1H, dd, 5.0, 9.4)	124.5	1.64[1] (2H, m)	22.3
3	2.83 (1H, brd, 20.4)	34.8	5.70 (1H, brd, 5.0)	117.8	1.98 (1H, ddd, 5.5, 12.9, 12.9)	36.3
	2.97 (1H, brd, 20.4)				2.38 (1H, dddd, 2.1, 2.1, 4.1, 13.2)	
4		144.7		139.5		148.5
5	2.66[1] (1H, m)	46.2	1.68[1] (1H, dd, 5.0, 12.1)	47.6	1.85[1] (1H, brd, 13.2)	50.0
6	2.62[1] (1H, m)	22.1	2.01 (1H, dd, 12.4, 14.0)	26.8	2.30[1] (1H, m)	25.7
	2.79 (1H, dd, 2.8, 16.0)		2.79 (1H, dd, 5.0, 13.8)		2.72 (1H, dd, 3.9, 13.8)	
7		148.4		160.8		162.5
8		148.2	4.64 (1H, dd, 5.8, 11.8)	78.7	4.83 (1H, dd, 6.1, 11.6)	78.0
9	5.76 (1H, s)	117.5	1.18 (1H, dd, 12.1, 12.1)	45.5	1.13 (1H, dd, 11.8, 11.8)	47.5
			2.41 (1H, dd, 6.1, 12.7)		2.30[1] (1H, m)	
10		39.7		36.4		37.0
11		120.9		119.0		120.2
12		171.2		175.1		174.8
13	1.92 (3H, s)	8.5	1.82 (3H, brs)	8.2	1.82 (3H, brs)	8.2
14	0.99 (3H, s)	23.4	0.96 (3H, s)	24.8	0.89 (3H, s)	16.4
15	4.81 (1H, s)	108.3	1.87 (3H, brs)	22.3	4.60 (1H, brs)	106.9
	5.04 (1H, s)				4.87 (1H, brs)	

[1] overlapped signal

Table S5. ^{1}H and ^{13}C NMR data (600 and 150 MHz, respectively) for compounds **21** and **22** ($CDCl_3$, δ in ppm, J in Hz, s singlet, d doublet, br broad, m multiplet).

No.	**21**		**22**	
	δ_H	δ_C	δ_H	δ_C
1	5.89 (1H, dd, 11.0, 17.6)	146.3	2.75[1] (1H, m)	50.4
2	5.18 (1H, d, 11.0)	113.2	4.00 (1H, ddd, 6.2, 6.2, 6.2)	82.1
	5.21 (1H, d, 17.6)			
3	4.68 (1H, brs)	113.9	1.61 (1H, ddd, 6.5, 6.5, 12.8)	38.1
	4.82 (1H, brs)		1.83[1] (1H, m)	
4		147.1	2.78[1] (1H, m)	30.1
5	2.58 (1H, dd, 1.7, 7.2)	49.4	2.26[1] (1H, m)	59.6
6	2.65 (1H, dd, 7.1, 14.9)	28.6		195.6
	2.70 (1H, dd, 1.4, 14.6)			
7		161.9		120.9
8	4.86 (1H, dd, 6.3, 11.8)	78.1		158.7
9	1.67 (1H, dd, 12.4, 12.4)	39.2	6.33 (1H, brs)	116.9
	2.31 (1H, dd, 6.3, 12.9)			
10		40.7		147.7
11		120.8		122.4
12		174.7	7.04 (1H, brs)	138.1
13	1.80 (3H, brs)	8.2	2.24 (3H, brs)	10.2
14	1.00 (3H, s)	27.9	2.14 (3H, brs)	22.1
15	1.71 (3H, brs)	25.3	1.08 (3H, d, 7.2)	22.0
1′			3.30 (3H, s)	56.4

[1] overlapped signal

Table S6. ^{1}H and ^{13}C NMR data (600 and 150 MHz, respectively) for compound **25** and (400 and 100 MHz, respectively) for **26** and **27** ($CDCl_3$, *δ* in ppm, *J* in Hz, s singlet, d doublet, br broad, m multiplet).

No.	25		26		27	
	δH	*δC*	*δH*	*δC*	*δH*	*δC*
1	1.47[1] (1H, m)<br>1.94[1] (1H, m)	39.5	0.98 (1H, ddd, 4.4)<br>1.69[1] (1H, m)	39.0	1.62[1] (2H, m)	34.3
2	2.46 (1H, ddd, 4.4, 7.7, 16.0)<br>2.51 (1H, ddd, 7.4, 9.1, 15.7)	34.0	1.59[1] (2H, m)	27.4	2.21 (1H, ddd, 7.3, 10.5, 15.2)<br>2.41[1] (1H, m)	28.2
3		217.5	3.20 (1H, dd, 5.0, 11.2)	78.9		179.8
4		47.4		39.0		147.5
5	1.40 (1H, dd, 3.0, 11.2)	55.2	0.76 (1H, dd, 3.2, 11.8)	56.0	2.00 (1H, dd, 3.8, 11.9)	51.0
6	1.55[1] (2H, m)	19.6	1.51[1] (2H, m)	18.3	1.39[1] (1H, m)<br>1.87 (1H, ddd, 3.3, 12.9, 25.9)	24.7
7	1.27 (1H, m)<br>1.58 (1H, m)	34.7	1.29[1] (1H, m)<br>1.57[1] (1H, m)	35.5	1.25 (1H, ddd, 3.2, 3.2, 12.9)<br>1.62[1] (1H, m)	34.1
8		39.5		39.9		39.6
9	1.56[1] (1H, m)	49.8	1.46[1] (1H, m)	50.9	1.64[1] (1H, m)	41.4
10		36.9		37.4		39.3
11	1.42[1] (1H, m)<br>1.65[1] (1H, m)	21.8	1.27[1] (1H, m)<br>1.59[1] (1H, m)	21.9	1.34[1] (1H, m)<br>1.49[1] (1H, m)	22.4
12	1.54[1] (1H, m)<br>1.90[1] (1H, m)	26.7	1.43[1] (1H, m)<br>1.72[1] (1H, m)	23.9	1.45[1] (1H, m)<br>1.74[1] (1H, m)	23.8
13	2.06 (1H, ddd, 3.3, 12.1, 12.1)	41.7	2.72 (1H, m)	47.7	2.72 (1H, m)	47.7
14		45.5		53.0		53.4
15	1.96 (1H, d, 15.4)<br>2.20 (1H, d, 16.4)	49.4	1.69[1] (1H, m)<br>2.35 (1H, brd, 15.9)	39.9	1.70[1] (1H, m)<br>2.35[1] (1H, brd)	40.0
16		223.9	5.65 (1H, m)	130.0	5.65 (1H, m)	130.0
17	2.23 (1H, d, 12.1)	57.2	5.56 (1H, m)	134.1	5.56 (1H, m)	134.0
18	1.09 (3H, s)	15.6	1.01 (3H, s)	18.3	1.07 (3H, s)	17.9
19	0.97 (3H, s)	15.9	0.85 (3H, s)	16.1	0.87 (3H, s)	20.0
20		74.4				
21	1.11 (3H, s)	24.9				
22	1.45[1] (2H, m)	41.1				
23	1.96[1] (1H, m)<br>2.15[1] (1H, m)	21.9				
24	5.06 (1H, dd, 6.9, 6.9)	124.2				
25		131.6				
26	1.61 (3H, s)	17.8				
27	1.68 (3H, s)	25.8				
28	0.98 (3H, s)	16.8	0.78 (3H, s)	15.3	4.68 (1H, d, 1.5)	113.5
29	1.05 (3H, s)	21.0	0.98 (3H, s)	28.0	4.86 (3H, brs)	23.2
30	1.09 (3H, s)	26.7	0.99 (3H, s)	17.1	1.74 (3H, s)	17.0

[1] overlapped signal

Table S7. ^{1}H and ^{13}C NMR data (600 and 150 MHz, respectively) for compounds **28**, **29** and (400 and 100 MHz, respectively) for **30** ($CDCl_3$, δ in ppm, *J* in Hz, s singlet, d doublet, br broad, m multiplet).

No.	**28**		**29**		**30**	
	δ_H	δ_C	δ_H	δ_C	δ_H	δ_C
1	3.58 (1H, dd, 2.9)	73.7	3.54 (1H, d, 3.1)	75.3	4.63 (1H, dd, 2.8, 2.8)	76.3
2	1.74 (1H, ddd, 2.9, 12.7, 12.7)	36.6	3.63 (1H, brd, 9.8)	72.6	1.75 (1H, ddd, 2.8, 12.2, 14.0)	34.2
	1.90[1] (1H, m)				2.01 (1H, ddd, 3.0, 4.4, 14.0)	
3	3.74 (1H, dd, 4.4, 12.1)	73.8	3.48 (1H, d, 9.0)	78.3	3.57 (1H, dd, 4.4, 12.1)	74.2
4		40.5		40.1		40.2
5	1.89[1] (1H, m)	39.5	1.92[1] (1H, m)	39.3	1.86[1] (1H, m)	40.7
6	0.83 (1H, dd, 2.4, 12.4)	20.8	0.82[1] (1H, m)	20.3	0.87[1] (1H, m)	20.5
	1.66[1] (1H, m)		1.63[1] (1H, m)		1.65[1] (1H, m)	
7	1.09[1] (1H, m)	25.7	1.14[1] (1H, m, 2.4, 12.5)	25.6	1.15[1] (1H, m)	25.1
	1.33[1] (1H, m)		1.34[1] (1H, m)		1.37[1] (1H, m)	
8	1.49 (1H, dd, 4.5, 12.5)	48.0	1.52 (1H, dd, 4.6, 12.3)	47.9	1.58[1] (1H, m)	46.6
9		20.8		20.6		21.1
10		30.3		29.1		28.2
11	1.30[1] (1H, m)	26.1	1.26[1] (1H, m)	26.2	1.21[1] (1H, m)	26.0
	2.19 (1H, ddd, 8.5, 8.7, 14.6)		2.28 (1H, ddd, 8.6, 8.6, 15.0)		1.86[1] (1H, m)	
12	1.67[1] (2H, m)	32.9	1.67[1] (2H, m)	32.8	1.54[1] (2H, m)	32.7
13		45.1		45.2		45.1
14		48.8		48.8		48.8
15	1.29[1] (2H, m)	35.7	1.29[1] (2H, m)	35.7	1.28[1] (2H, m)	35.5
16	1.291 (1H, m)	28.1	1.29[1] (1H, m)	28.1	1.29[1] (1H, m)	28.0
	1.90[1] (1H, m)		1.91[1] (1H, m)		190[1] (1H, m)	
17	1.59[1] (1H, m)	52.3	1.59[1] (1H, m)	52.3	1.57[1] (1H, m)	52.2
18	0.95[1] (3H, s)	18.2	0.95 (3H, s)	18.1	0.93 (3H, s)	17.8
19	0.46 (1H, d, 4.6)	30.0	0.48 (1H, d, 4.4)	29.4	0.44 (1H, d, 4.4)	28.3
	0.71 (1H, d, 4.6)				0.74 (1H, d, 4.4)	
20	1.38[1] (1H, m)	35.9	0.72 (1H, d, 4.4)	35.9	1.38[1] (1H, m)	35.9
21	0.88 (3H, d, 6.4)	18.2	1.38[1] (1H, m)	18.2	0.87 (3H, d, 6.6)	18.2
22	1.05[1] (1H, m)	36.3	0.89 (3H, d, 6.4)	36.3	1.04[1] (1H, m)	36.3
	1.44[1] (1H, m)				1.44[1] (1H, m)	
23	1.86[1] (1H, m)	25.0	1.04[1] (1H, m)	25.0	1.86[1] (1H, m)	24.9
	2.04[1] (1H, m)		1.43[1] (1H, m)		2.04[1] (1H, m)	
24	5.10 (1H, dd, 7.0, 7.0)	125.2	1.86[1] (1H, m)	125.2	5.10 (1H, dd, 7.2, 7.2)	125.2
			2.04 (1H, m)			
25		131.0	5.10 (1H, dd, 7.2, 7.2)	130.9		130.9
26	1.60 (3H, s)	17.7		17.7	1.60 (3H, s)	17.6
27	1.68 (3H, s)	25.8	1.60 (3H, s)	25.7	1.68 (3H, s)	25.7
28	0.92 (3H, s)	19.5	1.69 (3H, s)	19.4	0.91 (3H, s)	19.1
29	1.00 (3H, s)	25.2	0.95 (3H, s)	25.6	1.01 (3H, s)	25.0
30	0.79 (3H, s)	13.0	1.00 (3H, s)	14.3	0.80 (3H, s)	13.0
1′						170.3
2′					2.06 (3H, s)	21.4

[1] overlapped signal

Table S8. ^{1}H and ^{13}C NMR data (400 and 100 MHz, respectively) for compounds **31** and **32** and (600 and 150 MHz, respectively) for **33** ($CDCl_3$, *δ* in ppm, *J* in Hz, s singlet, d doublet, br broad, m multiplet).

No.	31		32		33	
	δ_H	δ_C	δ_H	δ_C	δ_H	δ_C
1	4.87 (1H, d, 3.5)	77.9	3.52 (1H, d, 3.3)	75.8	3.94 (1H, d, 2.9)	74.9
2	3.84 (1H, dd, 3.5, 10.0)	71.9	3.75 (1H, dd, 3.3, 10.5)	71.7	3.62 (1H, dd, 2.9, 9.4)	73.8
3	3.43 (1H, d, 10.0)	77.6	4.92 (1H, d, 10.5)	80.4	3.32 (1H, dd, 9.4, 9.4)	77.1
4		39.9		40.0	1.47 (1H, m)	36.5
5	1.89[1] (1H, m)	40.6	2.04 (1H, dd, 4.7, 12.4)	38.9	1.50 (1H, m)	39.9
6	0.86[1] (1H, m)	20.4	0.83[1] (1H, m)	20.6	1.32 (1H, m)	20.0
	1.63[1] (1H, m)		1.63[1] (1H, m)		1.79 (1H, m)	
7	1.14[1] (1H, m)	25.1	1.17[1] (1H, m)	25.6	2.17 (2H, m)	21.5
	1.37[1] (1H, m)		1.35[1] (1H, m)			
8	1.59[1] (1H, m)	46.7	1.52 (1H, dd, 4.4, 12.7)	47.9		139.0
9		20.8		20.3		130.0
10		28.0		28.8		42.3
11	1.22[1] (1H, m)	26.4	1.25[1] (1H, m)	26.1	2.06 (2H, m)	25.7
	1.85[1] (1H, m)		2.29[1] (1H, m)			
12	1.60[1] (2H, m)	32.7	1.68[1] (2H, m)	32.7	1.78 (2H, m)	30.9
13		45.1		45.2		44.6
14		48.9		48.8		50.2
15	1.28[1] (2H, m)	35.5	1.31[1] (2H, m)	35.7	1.19 (1H, m)	30.8
					1.59 (1H, m)	
16	1.29[1] (1H, m)	28.0	1.30[1] (1H, m)	28.1	1.32 (1H, m)	28.0
	1.89[1] (1H, m)		1.92[1] (1H, m)		1.93 (1H, m)	
17	1.57[1] (1H, m)	52.2	1.61[1] (1H, m)	52.3	1.51 (1H, m)	50.3
18	0.92 (3H, s)	17.9	0.96 (3H, s)	18.1	0.72 (3H, s)	15.7
19	0.53 (1H, d, 5.0)	28.2	0.51 (1H, d, 4.4)	29.3	0.99 (3H, s)	18.6
	0.81 (1H, d, 5.0)		0.72 (1H, d, 4.4)			
20	1.36[1] (1H, m)	35.9	1.39[1] (1H, m)	35.9	1.39 (1H, m)	36.3
21	0.86 (3H, d, 6.6)	18.2	0.89[1] (3H, m)	18.2	0.92 (3H, d)	18.7
22	1.04[1] (1H, m)	36.3	1.05[1] (1H, m)	36.3	1.04 (1H, m)	36.3
	1.42[1] (1H, m)		1.44[1] (1H, m)		not detected[1]	
23	1.85[1] (1H, m)	25.0	1.86[1] (1H, m)	24.9	1.86 (1H, m)	24.9
	2.03[1] (1H, m)		2.04[1] (1H, m)		2.03 (1H, m)	
24	5.09 (1H, dd, 7.1, 7.1)	125.2	5.10 (1H, dd, 7.2, 7.2)	125.2	5.10 (1H, dd, 7.0, 7.0)	125.2
25		131.0		130.9		131.0
26	1.60 (3H, s)	17.6	1.60 (3H, s)	17.6	1.60 (3H, s)	17.5
27	1.68 (3H, s)	25.7	1.69 (3H, s)	25.7	1.69 (3H, s)	25.8
28	0.91 (3H, s)	19.0	0.96 (3H, s)	19.4	0.92 (3H, s)	25.1
29	1.04 (3H, s)	25.6	0.88 (3H, s)	25.4	1.04 (3H, d, 5.9)	14.9
30	0.85 (3H, s)	14.4	0.89 (3H, s)	15.4		
1′	2.12 (3H, s)	172.0		175.0		
2′	0.85 (3H, s)	21.2	2.29 (2H, d, 7.2)	43.8		
3′			2.15 (1H, m)	25.7		
4′			1.00 (3H, d, 6.8)	22.4		
5′			1.00 (3H, d, 6.8)	22.5		

[1] overlapped signal.

Table S9. ^{1}H and ^{13}C NMR data (600 and 150 MHz, respectively) for compound **34** ($CDCl_3$, *δ* in ppm, *J* in Hz, s singlet, d doublet, br broad, m multiplet).

No.	**34**	
	δ_H	*δ_C*
1	1.45[1] (1H, m)	39.9
	1.92 (1H, ddd, 4.7, 8.0, 12.9)	
2	2.43 (1H, ddd, 4.5, 8.0, 15.8)	34.1
	2.50 (1H, ddd, 7.7, 9.6, 15.7)	
3		218.1
4		47.4
5	1.37[1] (1H, m)	55.3
6	1.47[1] (1H, m)	19.6
	1.56[1] (1H, m)	
7	1.32 (1H, m)	34.5
	1.56[1] (1H, m)	
8		40.3
9	1.42[1] (1H, m)	50.0
10		36.8
11	1.31[1] (1H, m)	22.0
	1.50[1] (1H, m)	
12	1.28[1] (1H, m)	27.5
	1.85 (1H, m)	
13	1.66[1] (1H, m)	42.4
14		50.3
15	1.09[1] (1H, m)	31.1
	1.47[1] (1H, m)	
16	1.49[1] (1H, m)	24.8
	1.75[1] (1H, m)	
17	1.75[1] (1H, m)	49.8
18	1.00 (3H, s)	15.2
19	0.95 (3H, s)	16.0
20		75.4
21	1.15[1] (3H, s)	25.5
22	1.48[1] (2H, m)	40.4
23	2.06 (2H, m)	22.6
24	5.13 (1H, t, 7.2)	124.7
25		131.7
26	1.63 (3H, s)	17.7
27	1.69 (3H, s)	25.7
28	0.89 (3H, s)	16.3
29	1.04 (3H, s)	21.0
30	1.08 (3H, s)	26.7

[1] overlapped signal.

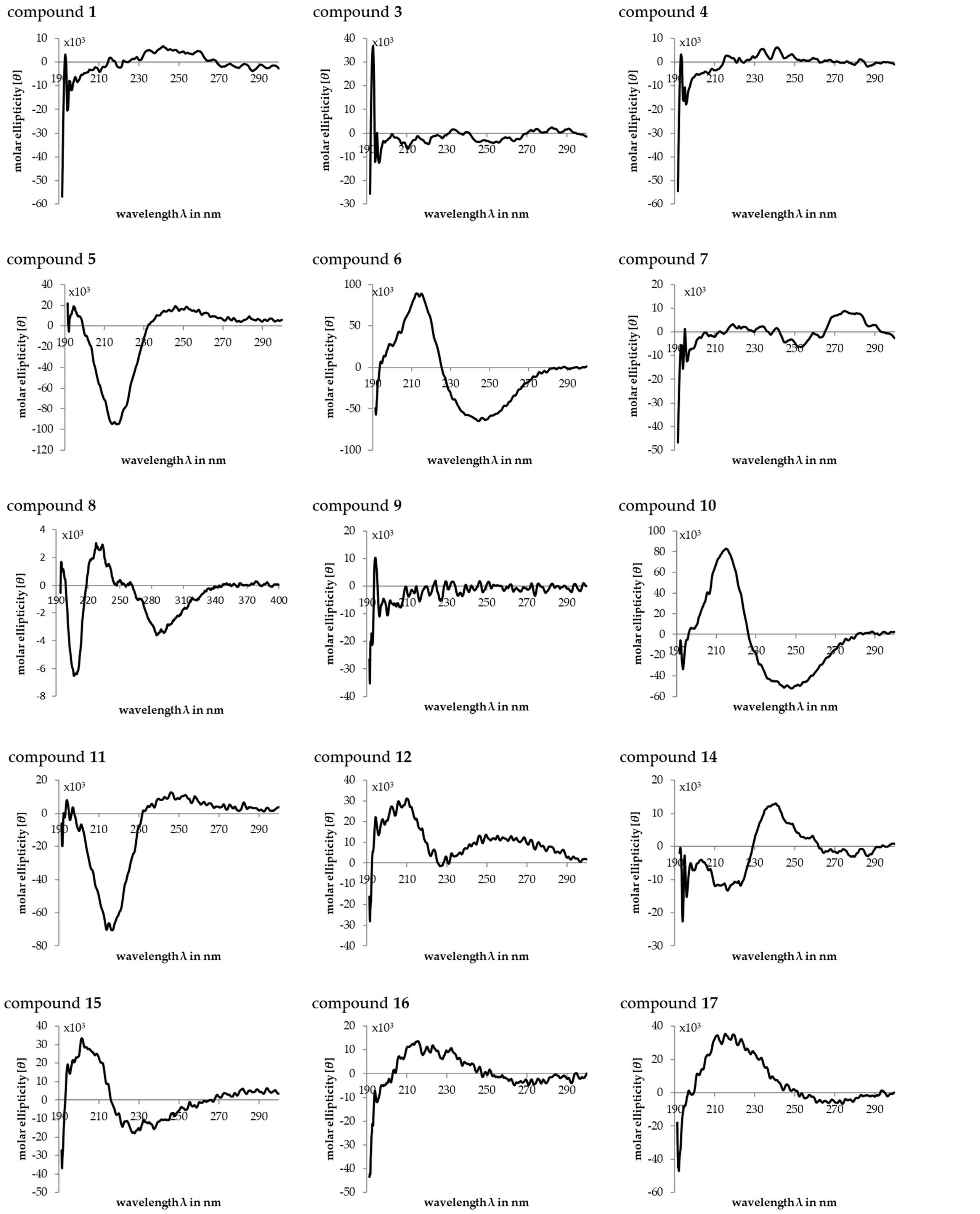

Figure S2. CD spectra of compounds **1**, **3-12** and **14-17** in methanol. [θ] in [(°cm^2) x dmol^{-1}].

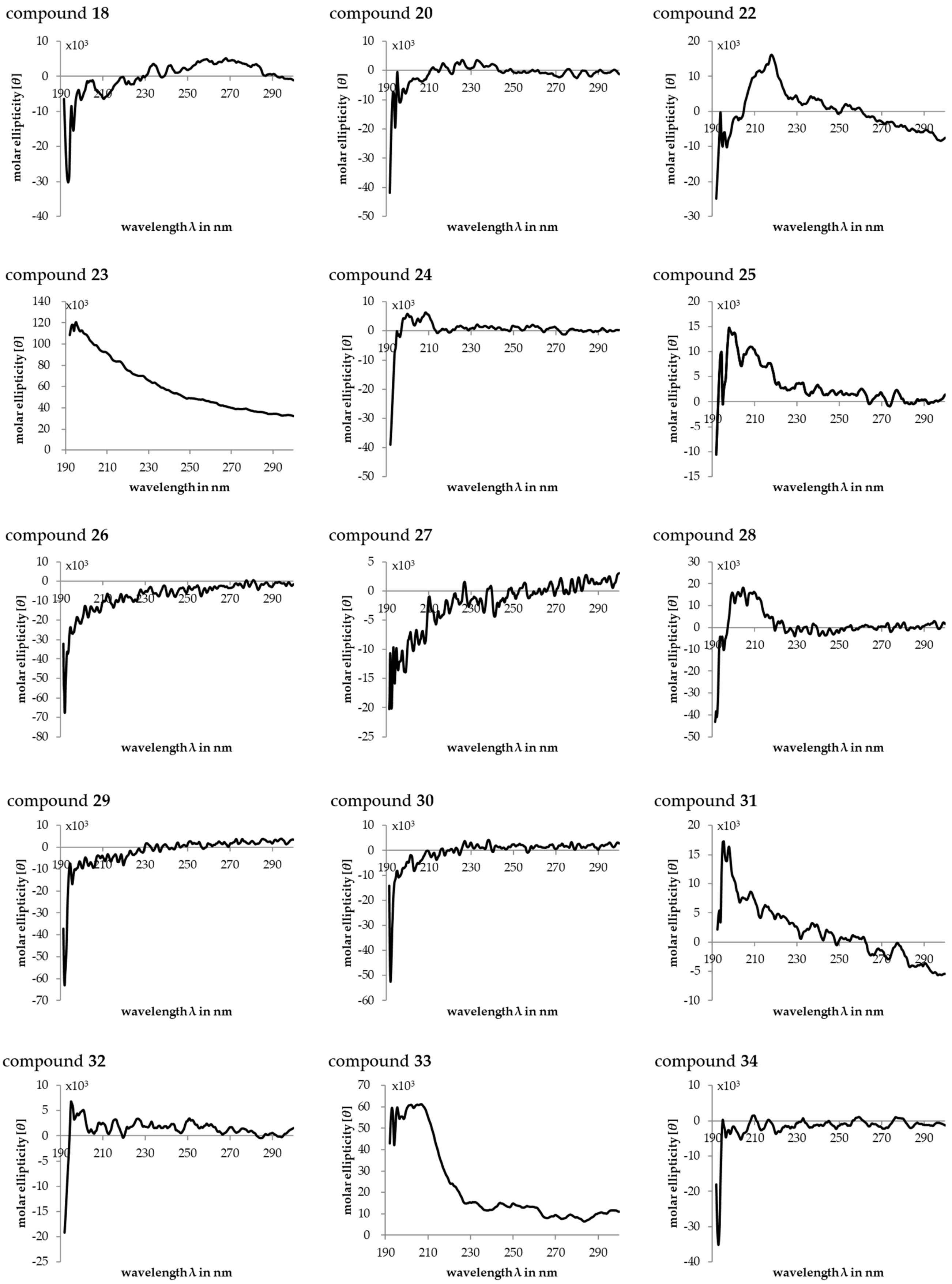

Figure S3. CD spectra of compounds **18**, **20** and **22**-**34** in methanol. [θ] in [(°cm^2) x dmol^{-1}].

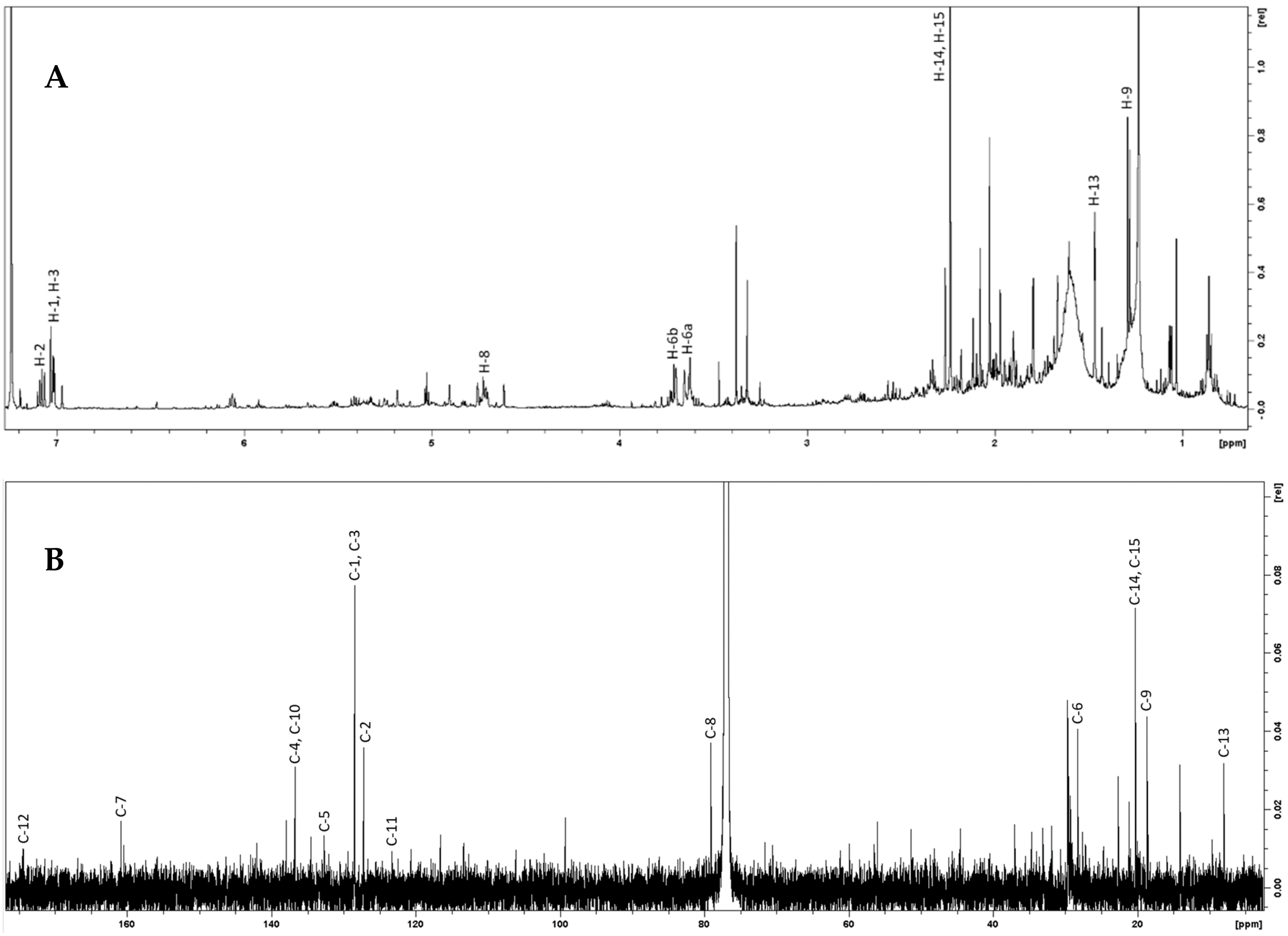

Figure S4. ^{1}H (600 MHz, **A**) and ^{13}C NMR spectra (150 MHz, **B**) of **1** (in $CDCl_3$).

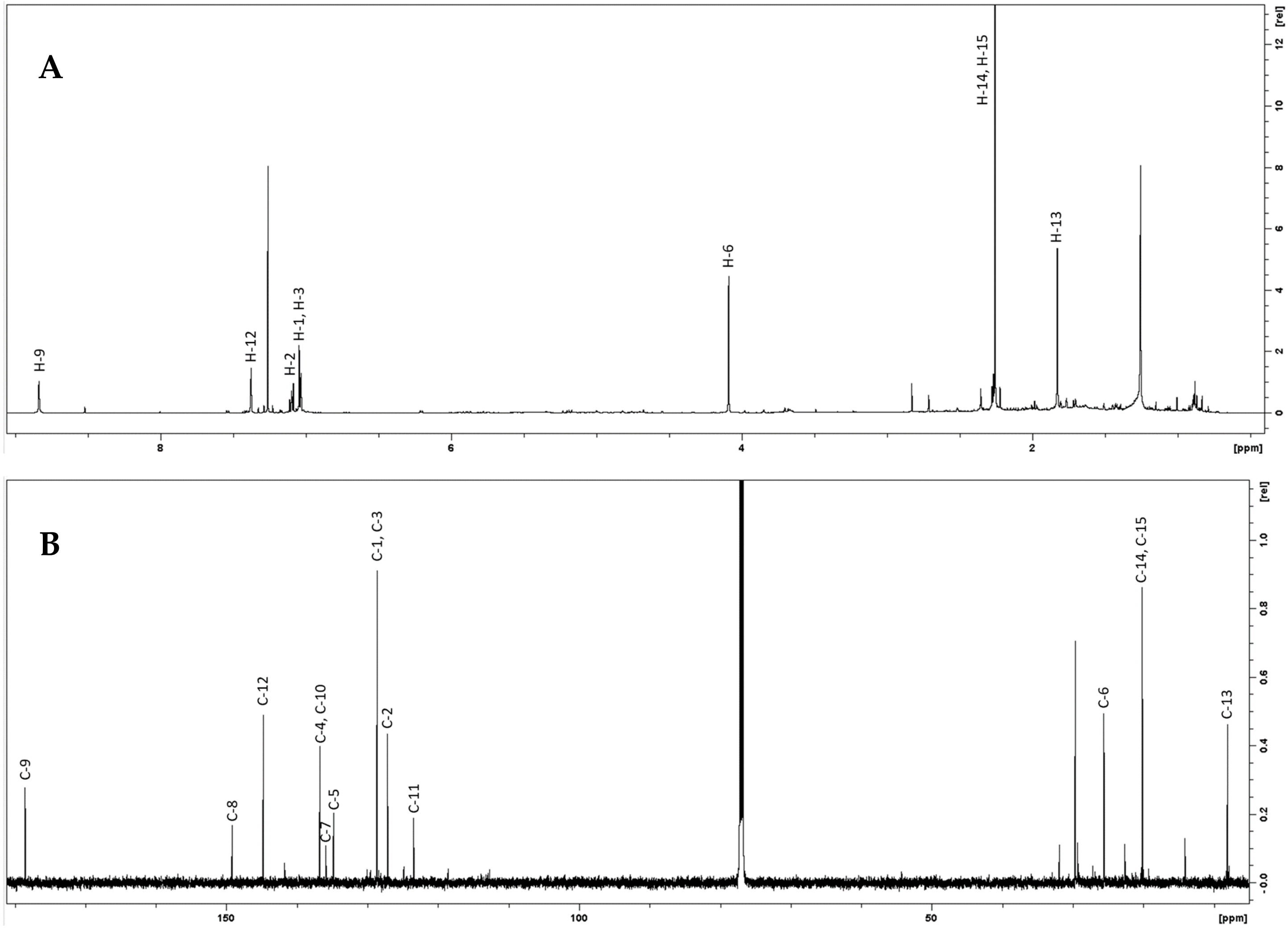

Figure S5. ^{1}H (600 MHz, **A**) and ^{13}C NMR spectra (150 MHz, **B**) of **2** (in $CDCl_3$).

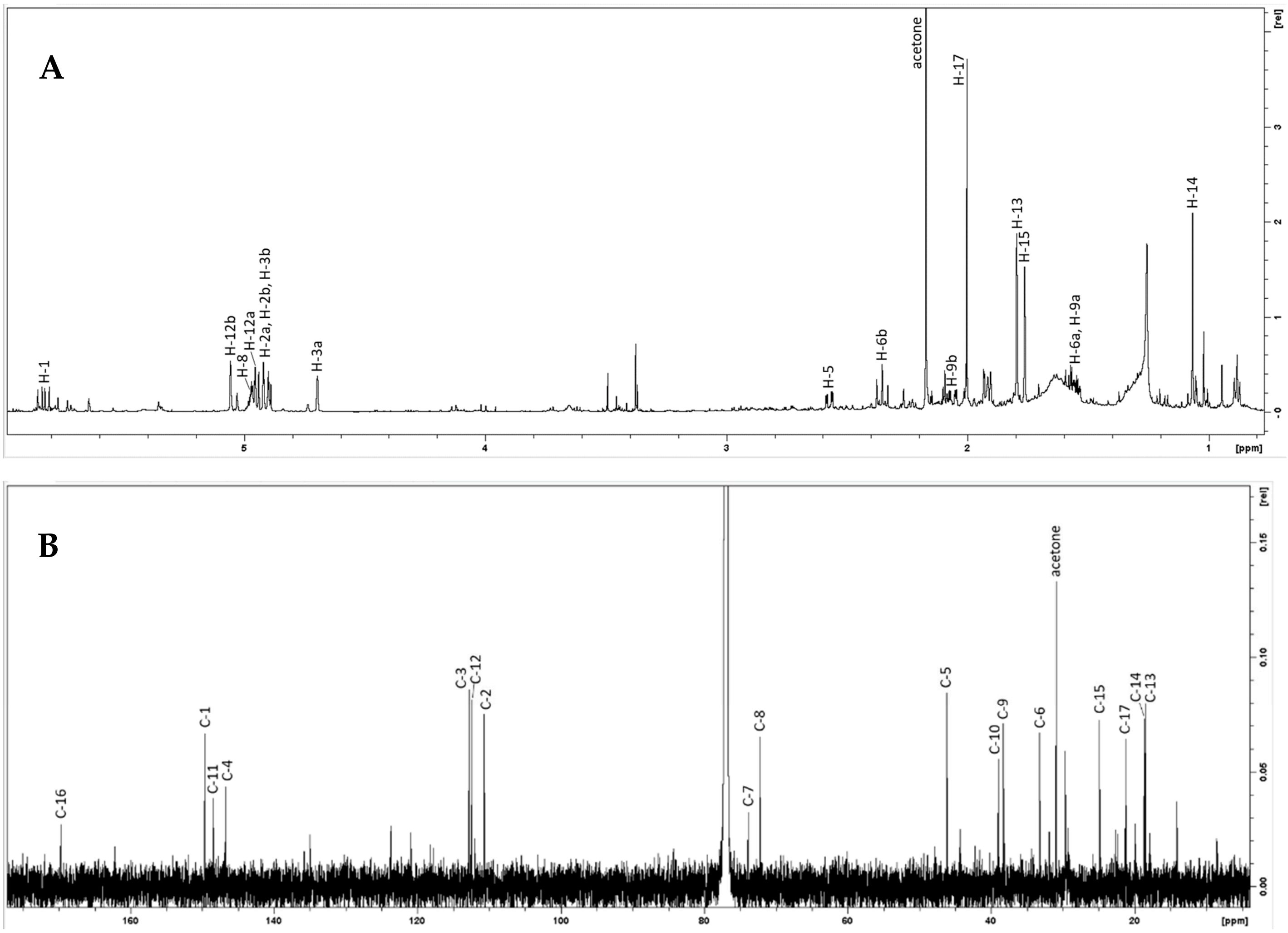

Figure S6. ^{1}H (600 MHz, **A**) and ^{13}C NMR spectra (150 MHz, **B**) of **3** (in $CDCl_3$).

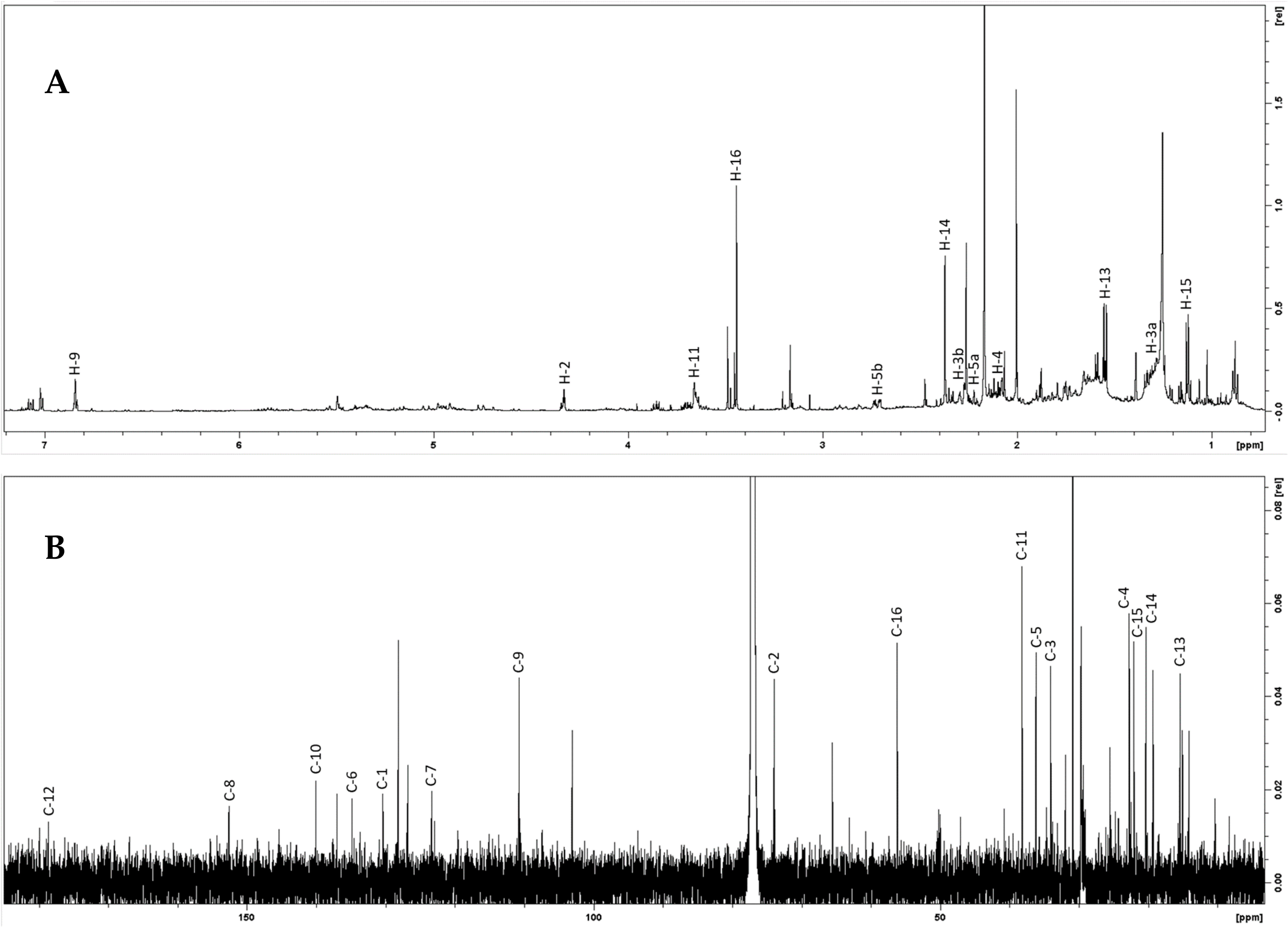

Figure S7. ^{1}H (600 MHz, **A**) and ^{13}C NMR spectra (150 MHz, **B**) of **4** (in $CDCl_3$).

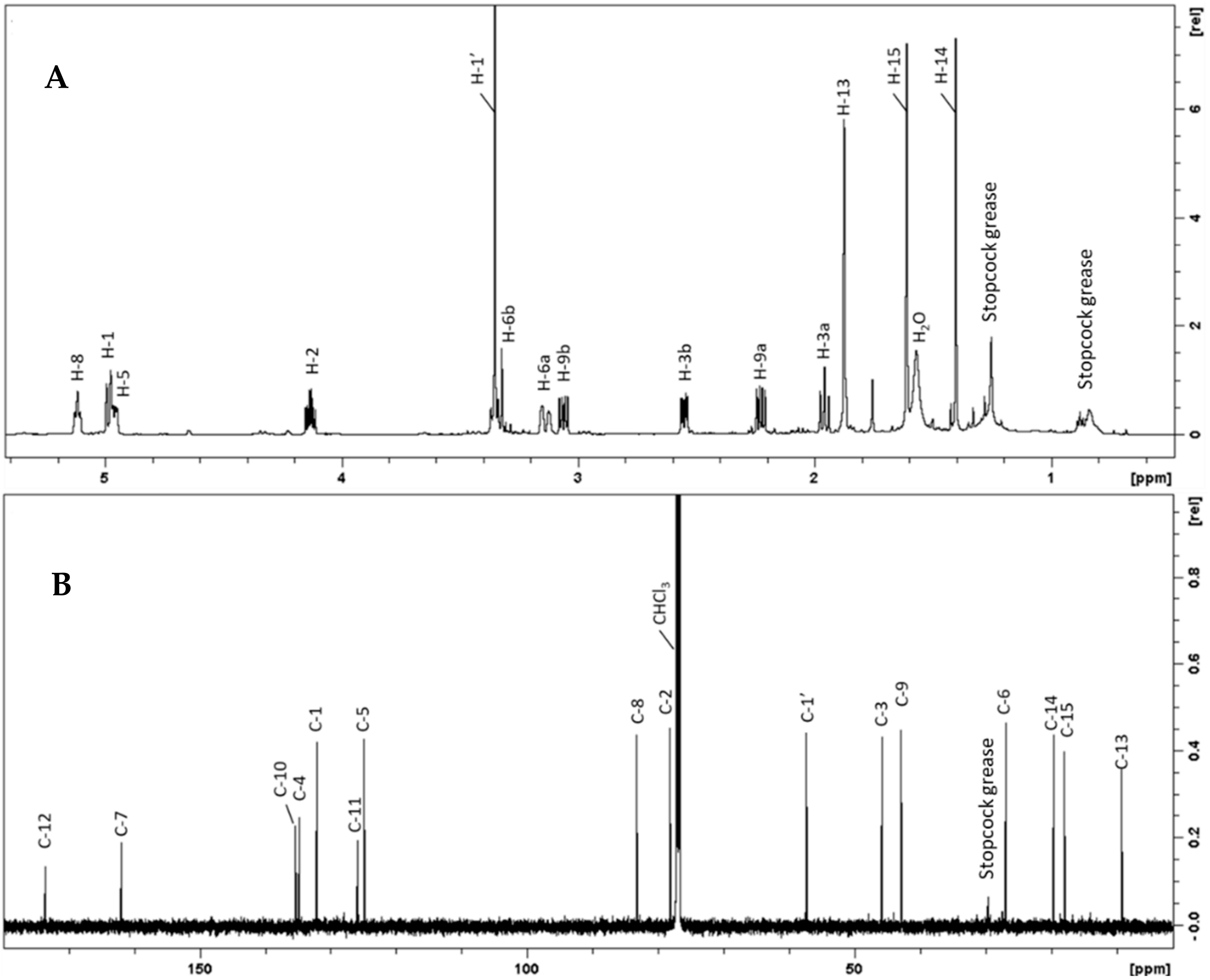

Figure S8. ^{1}H (600 MHz, **A**) and ^{13}C NMR spectra (150 MHz, **B**) of **5** (in $CDCl_3$).

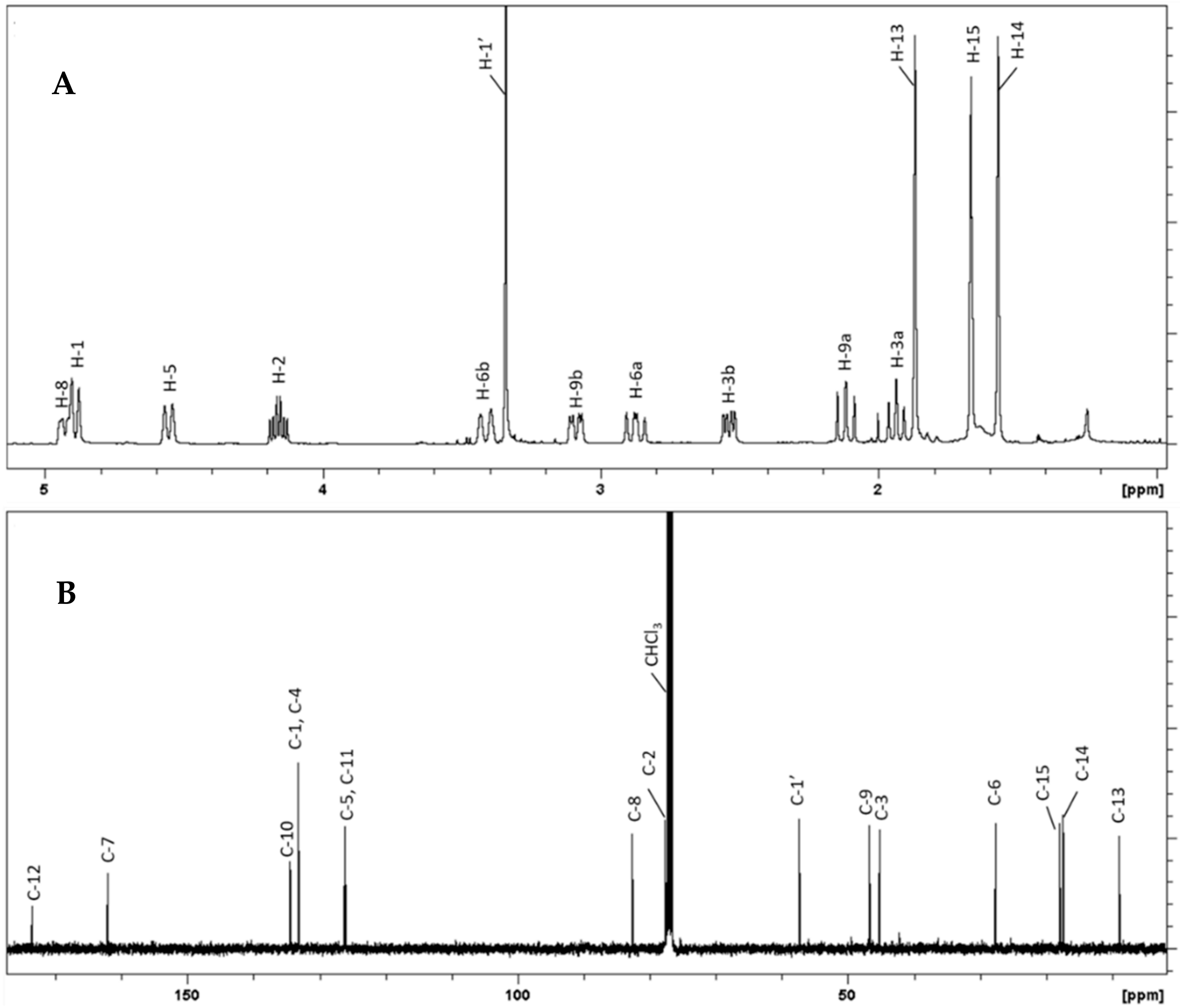

Figure S9. ^{1}H (400 MHz, **A**) and ^{13}C NMR spectra (100 MHz, B) of **6** (in $CDCl_3$).

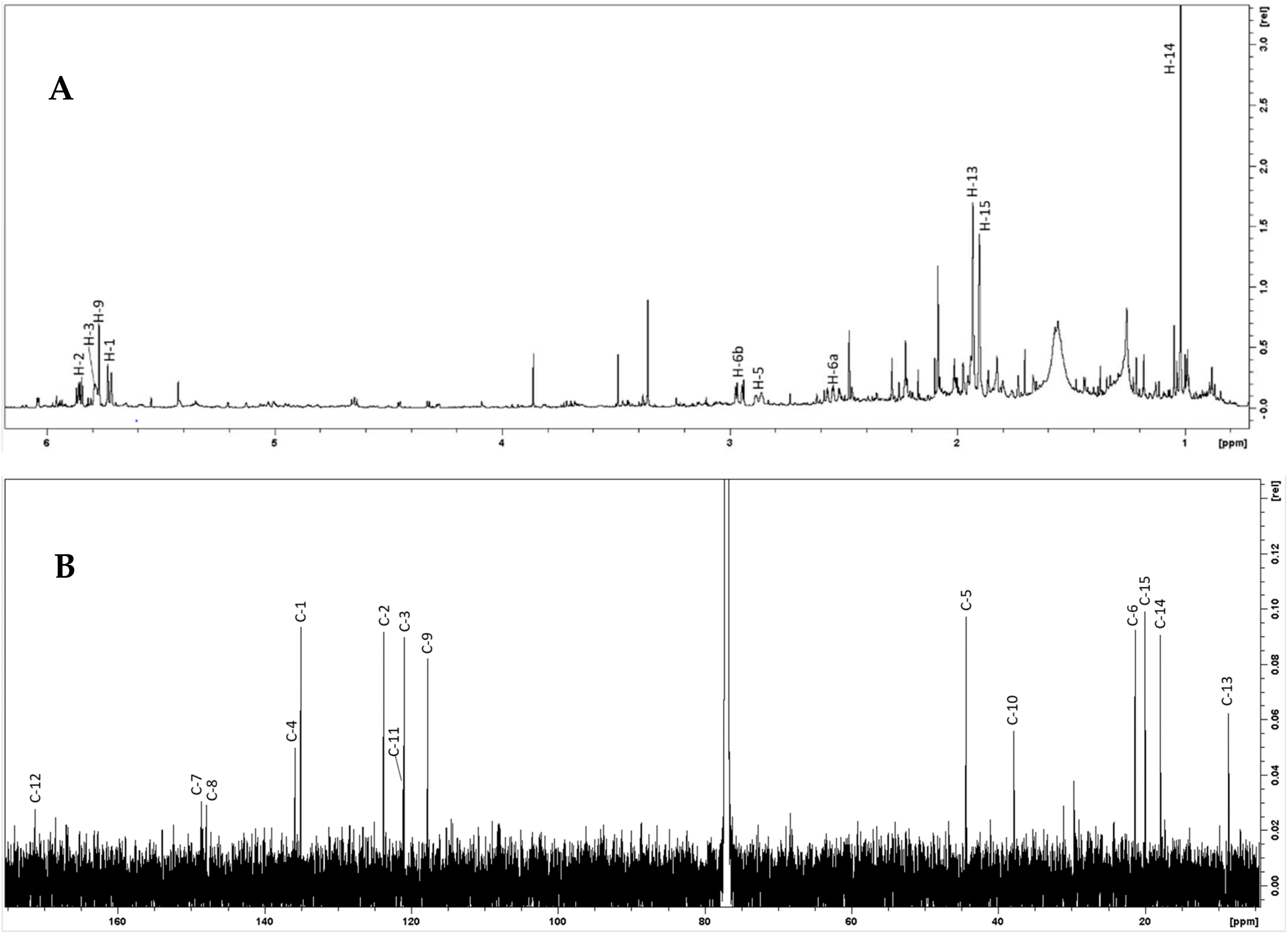

Figure S10. ^{1}H (600 MHz, **A**) and ^{13}C NMR spectra (150 MHz, **B**) of **7** (in $CDCl_3$).

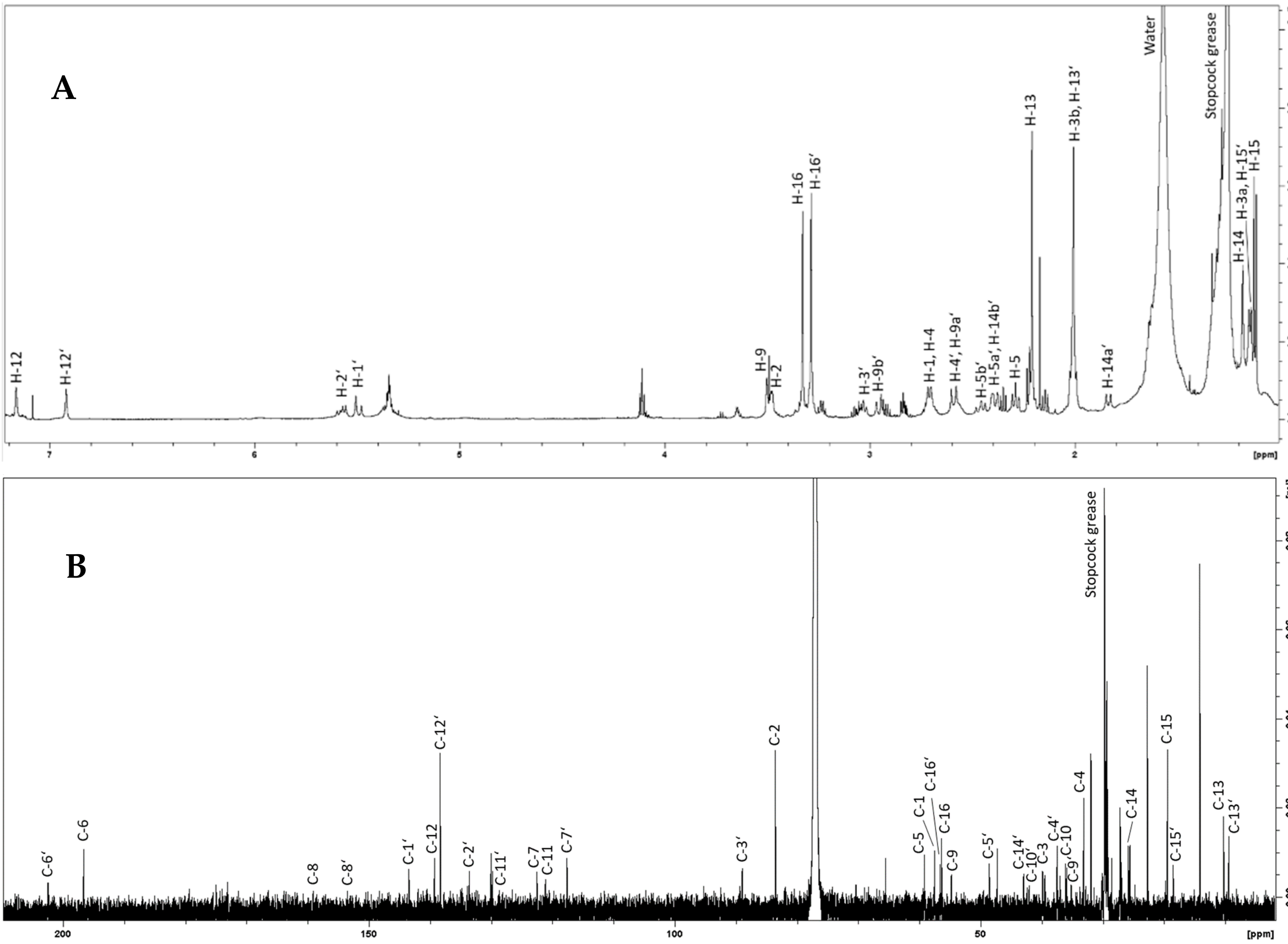

Figure S11. ^{1}H (600 MHz, **A**) and ^{13}C NMR spectra (150 MHz, **B**) of **8** (in $CDCl_3$).

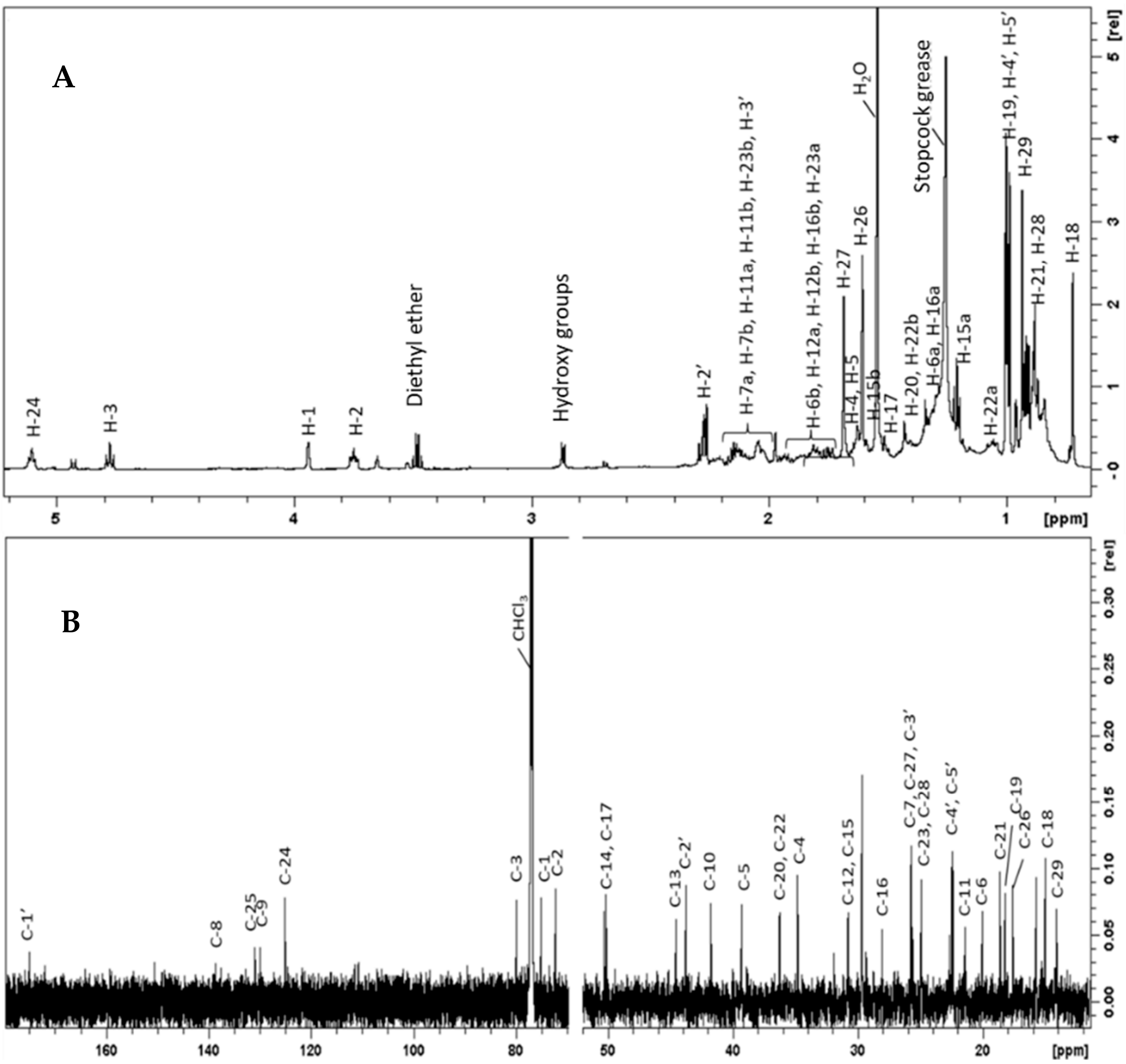

Figure S12. ^{1}H (600 MHz, **A**) and ^{13}C NMR spectra (150 MHz, **B**) of **23** (in $CDCl_3$).

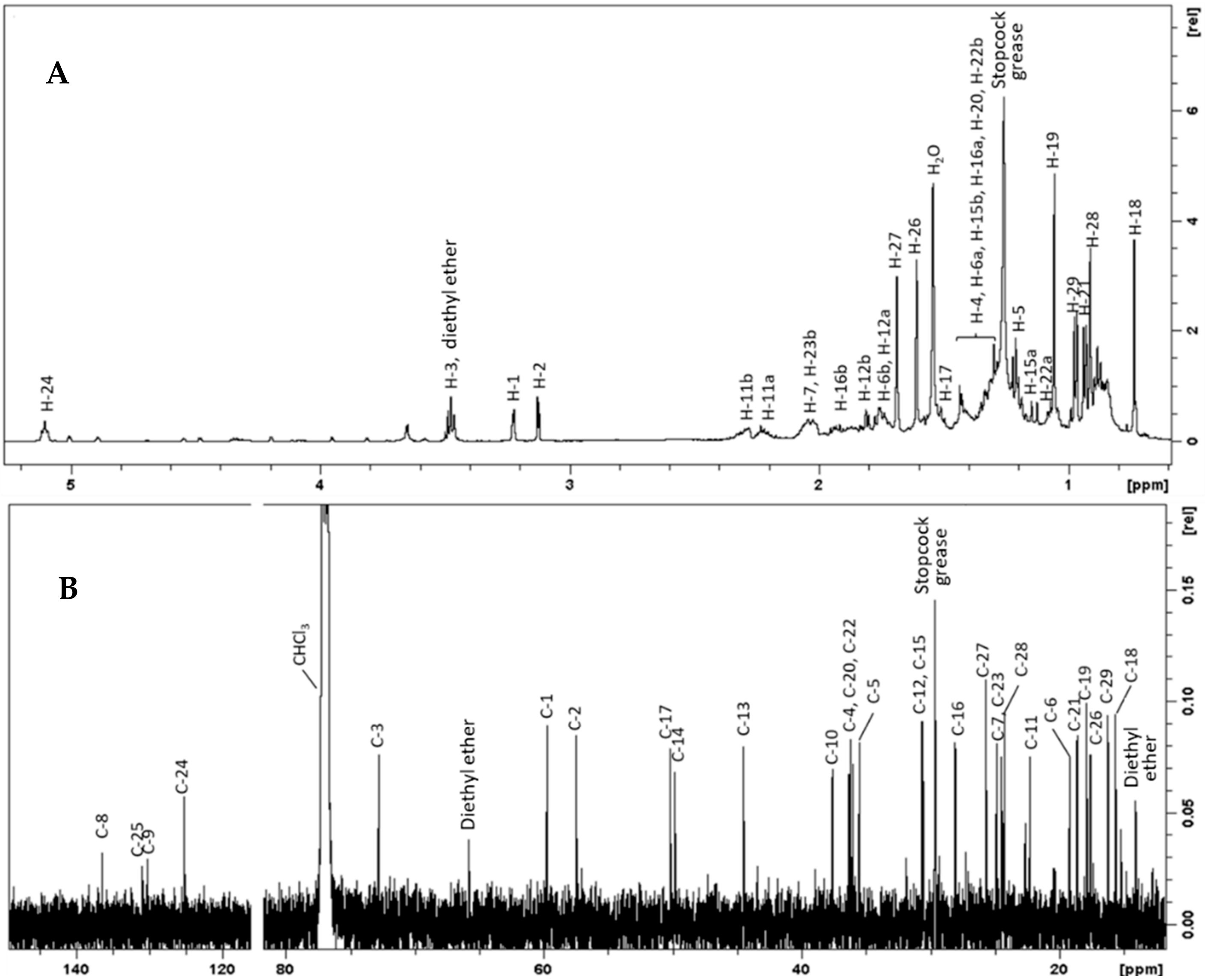

Figure S13. ^{1}H (600 MHz, **A**) and ^{13}C NMR spectra (150 MHz, **B**) of **24** (in $CDCl_3$).

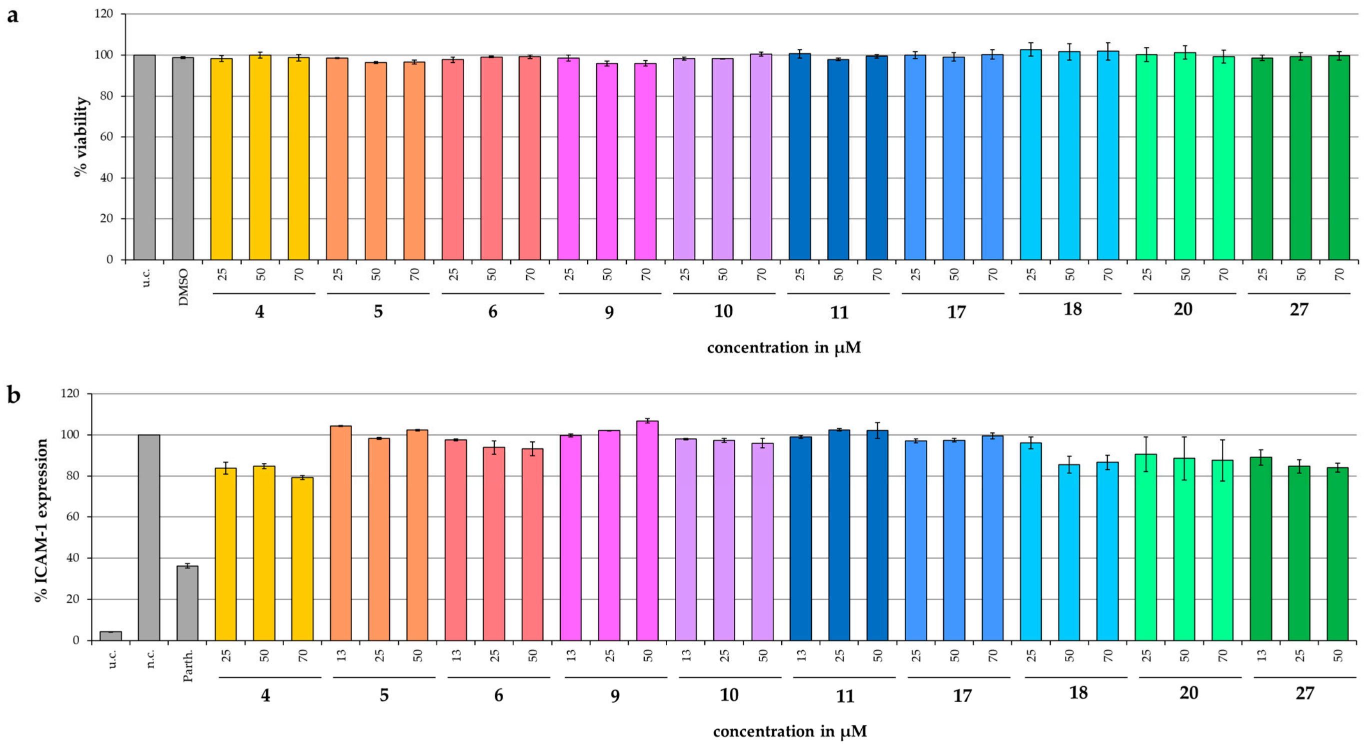

Figure S14. Influence of compounds **4**-**6**, **9**-**11**, **17**, **18**, **20** and **27** on the viability of HMEC-1 cells in the MTT assay (**a**) and on their TNFα induced ICAM-1 expression (**b**). The test was performed with medium containing DMSO (0.15%, v/v) without stimulation (u.c.), with TNFα (10 ng/mL, n.c.), and with both, TNFα and parthenolide (5 µM, Parth.) as positive control. Substances were applied in concentrations between 13-70 µM. Data are presented as mean ± SEM (n=3).
